# Is altitude a determinant of the health benefits of nature exposure? A systematic review and meta-analysis

**DOI:** 10.3389/fpubh.2022.1021618

**Published:** 2022-11-25

**Authors:** Eunsoo Kim, Sujin Park, Soojin Kim, Yeji Choi, Jae Hyoung Cho, Geonwoo Kim

**Affiliations:** Forest Human Service Division, Future Forest Strategy Department, National Institute of Forest Science, Seoul, South Korea

**Keywords:** nature-based intervention, forest therapy, psychological restoration, physiological relaxation, meta-regression

## Abstract

**Introduction:**

Nature exposure is a widely accepted option for promoting public health owing to the recent surge of scientific evidence. However, the actual settings to facilitate this initiative is yet to be extensively reviewed. In this systematic review, we have aimed to provide an up-to-date summary of interventional studies investigating the psycho-physiological effects of forests and urban forests, including details on their physical settings, and investigate an effect-modifying role of altitude and summarize data on the magnitude and shape of the association.

**Methods:**

A keyword search using five electronic academic databases (PubMed, Embase, PsycINFO, Web of Science, and Scopus) was conducted to identify relevant articles published in English from the inception year to the end of February 2022. The methodological quality was evaluated using the ROBINS-I or ROB2 tool, depending on the study design. Meta-regression and random effects model were jointly used to examine the relationship between altitude and health outcomes.

**Results:**

We included 27 eligible studies and 31 cases extracted from 19 studies were used for the meta-analysis. In the meta-regression, we observed a non-linear association between altitude and psycho-physiological effects. Altitude had a positive quadratic association with anxiety (*p* < 0.000, adjusted *R*^2^ = 96.79%), depression (*p* < 0.000, adjusted *R*^2^ = 98.78%), and fatigue (*p* < 0.000, adjusted *R*^2^ = 64.74%) alleviating effects. Conversely, altitude demonstrated a negative non-linear association with the blood pressure-lowering effect (*p* = 0.009, adjusted *R*^2^ = 32.83%). Additionally, the thermal index (THI) and illuminance (lx) levels were significantly associated with effect sizes of psychological restoration.

**Discussion:**

This review provides moderate-certainty evidence for an effect-modifying role of altitude. The meta-regression results suggested the optimal and minimal altitude ranges for psychological restoration and physiological relaxation, respectively. Despite some limitations, the study findings provide a significant basis for utilizing altitude, which is easily accessible and simple, to promote the health benefits of nature-based initiatives.

**Systematic review registration:**

https://www.crd.york.ac.uk/prospero/display_record.php?ID=CRD42022310894, identifier: CRD42022310894.

## Introduction

Historically, nature has empirically been used as a medium for psycho-physiological restoration. The commencement of earnest theoretical discussions in the 1980s have led to an active accumulation of scientific evidence on the restorative effects of nature (1, 2). Demands of daily life and stress may deplete psycho-physiological resources and result in heightened negative emotions, cognitive fatigue, and excessive physiological arousal (3–5). Persistent depletion can impair individuals' mental and physical health; hence, consistent restoration of psycho-physiological resources, analogous to the process of stress, is needed to remain healthy (6, 7). Nature exposure, which is rich in elements that can boost attention and restore cognitive resources without mental exertion, is a viable restoration process (8, 9). Furthermore, natural stimuli evoke moderate levels of interest and pleasure, which in turn elevate positive emotions, block negative thoughts, and enable a return to a moderate level of physiological arousal (10–12). Thus, nature exposure is linked to improved health *via* cognitive recovery, emotional restoration, stress reduction, and physiological relaxation.

A recent surge in scientific and clinical trials on nature and health has led to the social acceptance of nature-based interventions (13–16). Mounting evidence confirms the significant association between nature and health (1, 17–19); therefore, the World Health Organization and other health entities have emphasized nature as a health-promoting resource. Moreover, the recognition of nature as a non-pharmacological therapy in preventive and complementary medicine has penetrated the mainstream consciousness (20–22). Consequently, several countries have endeavored to quantitatively expand nature exposure (1, 23–26), and incorporated it into public health promotion practices (27–30). Notably, since the late 2000s, numerous forest-based initiatives have actively been implemented across East Asia, Europe, and North America to promote public health (28, 29, 31). Japan has introduced a forest certification system to maintain the quality of forest therapy, and emphasizes medical and scientific evidence (32). Korea has a license system for forest therapy, created healing forests, improved institutional framework, and trained therapists (33). Germany has promoted the use of forests for health promotion initiatives, including *klimatherapie, terinkur*, and *kneipp*; in 2019, a German state introduced legislation to ensure that forest therapy is covered by health insurance [(28), p. 321–336]. Additionally, forest-based interventions are officially employed across Europe and North America through green prescriptions, green exercise, and health tourism (34–37).

Several clinical trials concur that since all natural settings do not provide the same health benefits, the focus needs to shift from quantity to quality of nature (17, 38). Understanding the detailed characteristics of natural settings that determine the degree of health benefits is necessary to strengthen the evidence and systematize nature-based interventions (16, 21, 39, 40). Therefore, recent research is shifting from a simple dichotomous contrast between the natural and built environments to examining the variation in health effects according to the characteristics of natural settings. Previous studies have evaluated differences in health effects from an environmental psychological perspective using varied parameters, including the perceived amount of greenery (41–43), enclosure and openness of vegetation (44–48), and visual perception (49–59). Moreover, several studies have investigated the psychophysiological effects of different types of landscapes (60–69), and compared the health impacts of natural environments with varied ecological characteristics (64, 70–73) or silvicultural practices (2, 70–78). While recent research has predominantly focused on visual experience or ecological aspects, physical environments or non-visual experiences remain largely unexplored.

In recent studies, physical variables including altitude, temperature, humidity, and illuminance, are increasingly reported for comparing environmental conditions between study sites. Although these are readily available and crucial components of outdoor experiences, their impacts on outcomes have rarely been investigated. Few studies have investigated the relationship between the physical variables and outcomes of nature-based interventions (70, 79). An et al. (70) reported that changes in temperature, humidity, and light spectrum of forest settings can modify physiological outcomes of forest bathing. Similarly, Park et al. (79) indicated that physical variables of in-forest settings were responsible for psychological restoration. Thus, the physical factors may be crucial for nature-based interventions. However, to the best of our knowledge, comprehensive reviews or quantitatively synthesized evidence to investigate the effect-modifying role of physical variables remains limited. Previous reviews often focused on the association between vegetation levels and heat-related mortality (80, 81), which provided limited implications for selecting appropriate natural settings for nature-based interventions. Therefore, we have conducted a systematic review with meta-analysis to summarize the evidence across interventional studies investigating the psycho-physiological effects of nature exposure along with the descriptions of the physical variables.

Forests are a representative environment for nature-based interventions, and environmental changes according to altitude are particularly distinct and dramatic. Hence, we have focused on interventional studies conducted in forests or urban forests comprising “all woodlands, groups of trees, and individual trees located in urban and peri-urban areas” (82). Moreover, existing literature implies the link between forest-based intervention and physical factors of the forest. In several countries, definitions pertaining to the therapeutic use of forests frequently refer to the use of the atmospheric and topographic properties of forests. For example, *shinrin-yoku*—Japanese forest use for therapeutic purposes—is defined as “taking in the forest atmosphere or forest bathing.” (83). In Germany, *kilmatherpie* is refers to the use of microclimatic elements to deal with disease, and *terrainkur* is defined as an exercise method utilizing the terrain properties of forest trails [(29), p. 31]. In Korea, the government enacted a legislation defining forest healing as immune-strengthening and health-promoting activities utilizing the various forest elements (84). There are six distinctive forest healing practices; namely, climate, plant, water, diet, psycho-, and exercise therapies (85). The Korea Forest Service identified thermal comfort, scenery, and aromatic substances as essential properties for forest therapy (86). Furthermore, Shin et al. (33) highlighted the health advantages of forest-based interventions derived from experiencing the physical conditions of forest environments. Therefore, we have assumed that altitude and relevant physical variables are effect-modifiers that cause differences in the health effects of forest-based interventions.

Herein, we have provided an up-to-date summary of interventional studies examining the psycho-physiological effects of forest-based interventions, including descriptions of the physical variables of forests. In addition, using meta-analysis, we have statistically investigated whether altitude could modify the health benefits of forest exposures and the shape and magnitude of the associations by pooling the psychological and physiological outcomes with corresponding physical conditions. The research question conformed to the PICOS (Population, Intervention, Comparison, Outcomes, and Study) framework (39, 87): “In general populations, what is the effect of altitude of forest-based interventions on psycho-physiological effect—emotional restoration, cognitive restoration, stress reduction, physiological relaxation—from interventional studies?”

## Methods

The Preferred Reporting Items for Systematic Reviews and Meta-Analysis (PRISMA) 2020 (88) and Cochrane Handbook for Systematic Reviews of Interventions (89) guidelines were followed. The PRISMA checklist is presented in Supplemental Table 1. This systematic review and meta-analysis were registered on PROSPERO (CRD42022310894) and OSF database (doi: https://doi.org/10.17605/OSF.IO/SG7TD) prior to commencement.

### PICOS and eligibility criteria

Our research question was framed and refined using PICOS to address a clearly formulated review question: “In general populations, what is the effect of altitude of forest-based interventions on psycho-physiological aspects—emotional restoration, cognitive restoration, stress reduction, physiological relaxation—in interventional studies?” (87, 89). In addition, the eligibility criteria following the PICOS framework, is presented in Table 1.

**Table 1:** Eligibility criteria for study selection.

**PICOS element**	**Inclusion criteria**	**Exclusion criteria**
Population	General population	Studies not including human participants
Intervention	(1) Structured programs or activities in forests or urban forests with specific health promotion purposes (2) Include description of forest settings in which the intervention was performed in terms of altitude or relevant variables (longitude and latitude, temperature, humidity, dew point, atmospheric pressure)	Studies not match with the defined intervention Studies not reporting altitude or relevant variables
Comparison(s)	Waitlist group, urban exposure group, normal daily routines, other comparative intervention with little or no nature exposure	Studies not including comparators with little or no nature exposure
Outcome	Studies reporting quantitative outcomes to derive effect estimates related to at least one of follows: (1) Emotional restoration measured by using POMS, PANAS, STAI, BDI, SVS, and other relevant self-reporting measurements (2) Cognitive restoration measured by using PRS, ROS, and other relevant self-reporting measurement (3) Stress reduction: (saliva) cortisol, (saliva) amylase, adrenaline, noradrenaline, serotonin, melatonin, and other relevant stress markers (4) Physiological relaxation: change in blood pressure, heart rate, pulse rate, heart rate variability, skin conductance, brainwave, prefrontal activity, SpO2, EEG, and other relevant biomarkers	Studies not reporting quantitative outcomes related to emotional restoration, cognitive restoration, stress reduction, or physiological relaxation.
Study design	Interventional studies such as randomized controlled trials, randomized cross-over, and non-randomized controlled studies	Non-interventional studies such as review, historical cohort, case-control, cross-sectional study

### Search strategy

A literature search using five electronic academic databases—PubMed, Embase, PsycINFO, Web of Science, and Scopus—was performed. Published articles in English, from the inception year to the end of February 2022, were searched using a combination of search terms related to environmental setting (21 terms), intervention (48 terms), altitude or location (71 terms), health outcomes (69 terms), and study designs (16 terms). Details on the search terms are presented in Supplemental Table 2 and are publicly available (DOI: https://www.crd.york.ac.uk/PROSPEROFILES/310894_STRATEGY_20220215.pdf).

#### Study selection process

The search results were exported to the EndNote Citation Manager software (Endnote 20.3, Clarivate Analytics, London, UK). After de-duplication, two investigators (EK and SK) independently screened the titles and abstracts to exclude explicitly irrelevant cases, and subsequently, conducted a full-text review based on the eligibility criteria. In case of discrepancies, both investigators conducted a second full-text review and consensus-based discussion to determine eligibility for inclusion. In case of conflicting views, two other investigators were consulted to resolve the discrepancies (GK and SP).

#### Data extraction

The data from the included studies were independently extracted by two investigators (GK and EK), using the same data extraction form, and were cross-checked. The extracted data included (a) study information (author, year of publication, country, city, study design, conducted date, and time of measurement); (b) sample (sample size, gender, and age); (c) forest variables [altitude, location, longitude, latitude, dominant tree species, height (m), diameter at breast height (cm), stand density (trees/ha), canopy density (%)]; (d) environmental variables [temperature (°C), relative humidity (%), radiant heat (°C), wind speed (m/s), illuminance (lx), and noise level (dB)]; (e) intervention (activities, activity duration, and frequency); (f) outcome measurement [measurement indices, pre-measurement (M ± SD), post-measurement (M ± SD), change in measurement (M ± SD), and inter-trial correlation]. In studies where only locations were reported, coordinates were used to estimate altitudes. In studies where altitudes were mentioned as ranges, median or midpoint value was chosen for each forest exposure, depending on data availability. In studies reporting both temperature and humidity, the temperature humidity index (THI) was calculated as an indicator of bioclimatic conditions reflecting heat and cold stress (90, 91).

#### Methodological quality

The latest version of the Risk of Bias 2 (RoB2) tool was used to evaluate the methodological quality of randomized parallel-group trials and randomized crossover trials (92). For non-randomized trials, the Risk of Bias in Non-randomized Studies Interventions (ROBINS-I) tool (93) was used. The risk of bias that may have occurred in the randomization process, trial design, dropouts and missing data, and outcome measurement were evaluated *via* the RoB2 tool. The risk of bias that may have arisen due to confounding factors, participant selection, classification of intervention, dropout and missing data, outcome measurement, and reporting were assessed *via* the ROBINS-I tool. The risk of bias was independently assessed by two investigators (EK and GK) based on the answers for the signaling questions in five, six, and domains for randomized parallel-group trials, randomized crossover trials, and non-randomized trials, respectively.

#### Quantitative synthesis

Statistical analysis was performed using R 4.20 and R Studio with “meta,” “metafor,” and other R packages (94, 95). First, the effect size of the individual studies and the overall effect size were calculated. Subsequently, a series of meta-regressions were performed to verify whether altitude and related physical variables influenced the effect size of studies and the magnitude and shape of the association were investigated. Finally, sensitivity and publication bias analyses were conducted to check the robustness of our results.

#### Estimating overall effect size

Standardized mean difference (SMD) was calculated using a random-effects model. The SMD is a representative measure of efficacy computed using the mean, standard deviation, and number of samples of both interventional and control groups. An SMD of zero indicates that there is no difference in effect between the intervention and control. If improvement is related to higher scores on outcome measures, an SMD > 0 reflects the extent to which the intervention is more effective than the control. Conversely, if improvement is related to lower scores on the outcome measure, an SMD <0 reflects the extent to which intervention is less effective than the control. According to Cohen's (96) guidelines, the result of SMD 0.20–0.49, 0.50–0.79, and ≥0.80 as “small,” “medium,” and “large” effect sizes, respectively.

Since the effect of forest exposure was hypothesized to vary by altitude and relevant physical variables, a random-effects model—which assumed that the true effect size varies by study and is distributed around the overall mean— was used to estimate effect size. A restricted maximum-likelihood estimation, recommended in a recent simulation study (97), was employed to estimate the between-study variance in the random-effects model. The overall effect size was weighted by the inverse variance method. Cochran's *Q*-test (*p* < 0.10 for statistical significance) and the *I*^2^ (*I*^2^ > 50% used as a threshold for significant heterogeneity) was used to investigate heterogeneity in the effect sizes. Cochran's Q test is a statistical test to determine whether interventions have an identical effect. *I*^2^ is an index reflecting variance across studies attributable to heterogeneity, with 25–50, 50–75, and 75–100% indicating low, medium, and large heterogeneity, respectively (98).

#### Meta-regression

Meta-regression, a sophisticated tool for exploring heterogeneity, aims to identify whether a significant association exists between an outcome measure and one or more study-level variables. In our study, we assumed that altitude and physical variables were effect modifiers and conducted a meta-regression to further explore heterogeneity. A series of meta-regression were performed using both linear and non-linear models; non-linear models reportedly reflect phenomena better than linear models (99–101). Log-likelihood and Akaike's Information Criterion (AIC) were used for model comparison and a suitable association model was selected. Data on the magnitude of the association was summarized using the following test statistics: QM (omnibus test statistics of model coefficients used in moderator analysis); *R*^2^ (the amount of heterogeneity accounted for); model outcomes (regression coefficients, standard error, and confidence limits); and difference between total heterogeneity and regression heterogeneity. The effect size trends by altitude are graphically represented using predicted curves and 95% confidence intervals (CI).

#### Sensitivity analysis and publication bias

Individual studies' influence on the effect estimation was checked through rstudent, diffits, Cook's D, covratio, τ2, Qresid, hat, and dfbetas values. The “leave-one-out” method was used for sensitivity analysis. Publication bias was graphically and quantitatively assessed using a funnel plot and Egger's regression test, respectively (102).

### Certainty of evidence

The overall degree of certainty of evidence was evaluated using the GRADE method (103). Based on our research question, the certainty of altitudinal influence on psycho-physiological restoration provided by the meta-regression results was assessed. GRADEPro GDT (https://gradepro.org) was used to evaluate the certainty of evidence and create a Summary of Findings table (104).

## Results

### Study selection

**Figure 1:**
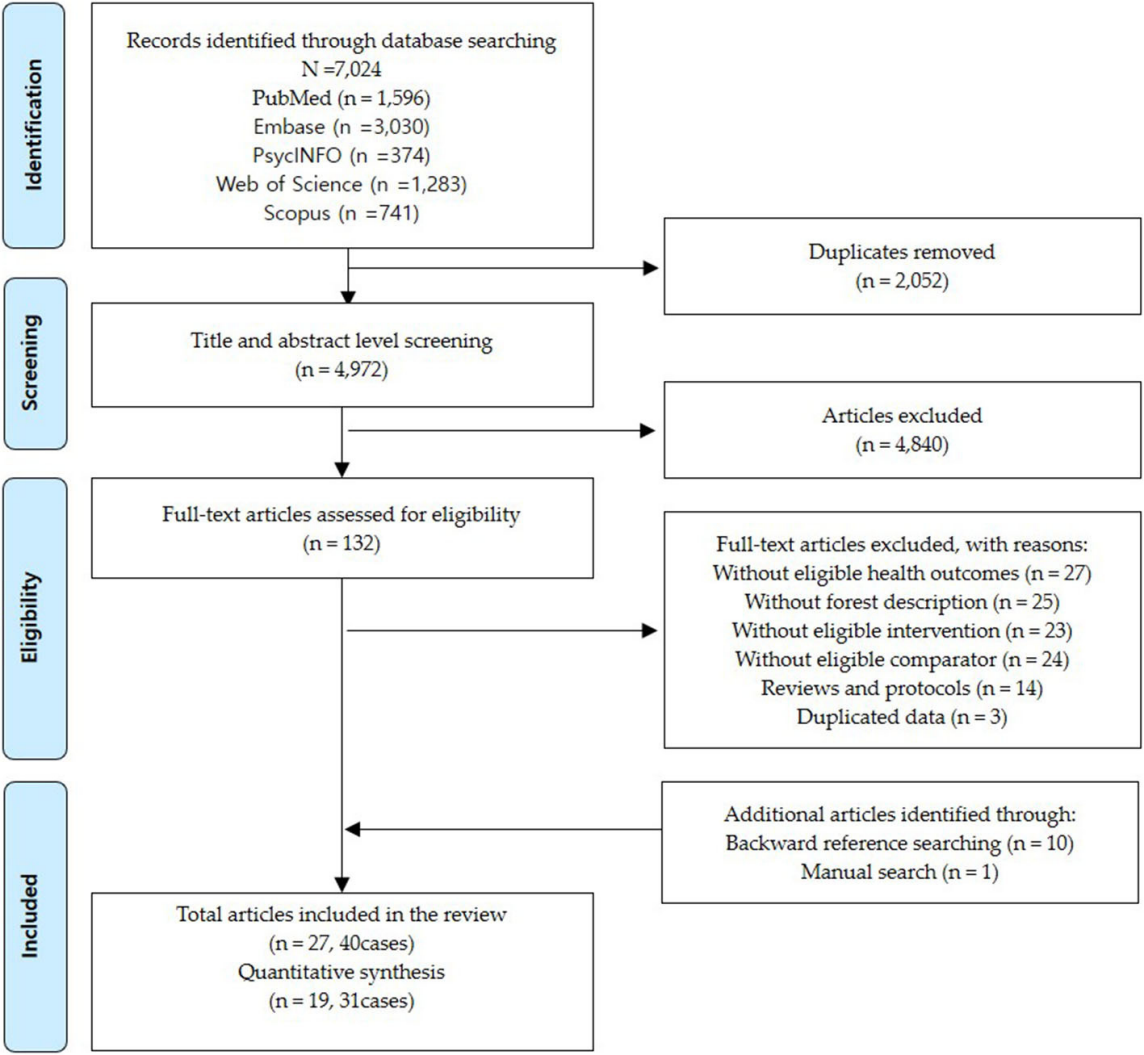
Flow diagram illustrating the selection process.

The database search identified 7,024 studies, from which 2,052 duplicates and 4,840 studies were excluded after title and abstract screening. After a full-text assessment of the remaining 132 studies, 114 were excluded for the following reasons: without eligible health outcome (*n* = 27), without forest description (*n* = 25), without eligible intervention (*n* = 23), without eligible comparator (*n* = 24), reviews (*n* = 9), protocols (*n* = 5), and duplicated publication (*n* = 3). Ten studies and one study were added through backward citation and manual searches, respectively. Finally, 27 studies complied with the eligibility criteria, and 31 cases extracted from 19 studies were selected for the quantitative synthesis. The PRISMA flow diagram of the study selection is presented in Figure 1.

### Study characteristics

Study characteristics of the 27 included studies are summarized in Table 2. The included studies performed nature-based interventions aimed at promoting health in urban forests and forests ranging in altitude from 11 to 1,324 m. The studies were published between 1995 and 2021; most were from East Asia (*N* = 16), followed by Northern Europe (*N* = 5), Central and Eastern Europe (*N* = 3), North America (*N* = 2), and Oceania (*N* = 1). The majority of studies (N=26) were conducted at latitudes between 28 and 60° N; the climate was typically temperate to humid continental, except one study (114) that was conducted at 19° S in semi-arid conditions. All the 26 study regions have distinct four-season patterns. The majority of studies conducted interventions between July and September [*N* = 11, temp 19.1–25.5°C, relative humidity (RH) 61.1–94.3%], followed by October–December (*N* = 5 temp 8–18°C, RH 52.3–79.0%), April–June (*N* = 4 temp 26.3–27.8°C, RH 39.9–44.3%), and January–March (*N* = 1, temp −0.5°C, RH 100.0%). Five studies did not indicate the precise period of the intervention, and one study carried out interventions throughout the year.

**Table 2:** Characteristics of the included studies ordered by first author's name and publication year.

**References**	**Population**	**Intervention**			**Comparator**	**Outcome**	**Study design**
		**Altitude**	**Location**	**Activity**			
Bielinis et al. (105)	Healthy female university students Age: 20.97 ± 0.65 Sample size (M/F): 32 (0/32)	130 m	Forest trail in Olsztyn named Las Gronicki , Poland (53°45′2"N 20°26′2"E)	Standing and viewing for 15 min	Urban street in Olsztyn city, Poland	PANAS; POMS; ROS; SVS	Randomized parallel-group trial
Bratman et al. (106)	Adults from the San Francisco Bay Area with no current or past diagnosis of neurologic or psychiatric disorder Age: 22.9 Sample size (M/F): 60 (27/33)	50 ~ 130 m	Park near Stanford University known as “The Dish,” USA	Walking and observing for 50 min	A busy street located on main thoroughfare through Palo Alto, USA	ANT; BDS; OSPAN; PANAS; RRQ; STAI	Randomized parallel-group trial
Chun et al. (107)	Chronic stroke patients aged from 36 to 79 (31 cerebral infarcts, 28 intracerebral hemorrhages) Age: 60.8 ± 9.1 Sample size (M/F): 59 (40/19)	450 ~ 600 m	Recreational forests in Gyenggi-do, Republic of Korea (37°46′1"N 127°20′2"E)	Attending the recreational forest site program for 3 nights 4 days	Staying and walking in a hotel in Gyeonggi city, Republic of Korea	BDI; HAM-D17; STAI	Randomized parallel-group trial
de Brito et al. (108)	Healthy middle-aged adults aged from 35 to 59 Age: 49.3 ± 6.7 Sample size (M/F): 24 (4/20)	295 ~ 315 m	Wood Duck Trail of the Minnesota Landscape Arboretum (MLA), USA (44°51′40"N 93°37′15"W)	Walking for 50 min	Paved sidewalks of medium traffic roads located in a medium-density residential area	HRV; SBP; DBP	Non-randomized controlled trial
Djernis et al. (109)	University students experiencing moderate to high levels of stress with no known psychiatric diagnosis Age: 30.60 ± 7.91 Sample size (M/F): 60 (8/52)	44 m	Therapy garden of University of Copenhagen, Denmark (55°52′01"N 12°30′28"E)	Attending mindfulness program for 5 nights 6 days	Indoor settings with views across a suburban area with office buildings and a car park	BCT; PSS	Randomized parallel-group trial
Dolling et al. (110)	Adults with high stress levels aged from 18 to 65 Age: 48 ± 12 Sample size (M/F): 56 (13/33)	89 ~ 92 m	Forest located in the boreal zone near lake Bäcksjön, Sweden (63°58′ N, 20°21′ E)	Walks, relaxation, woodcutting, gathering twigs, and branches for 2 h (twice per week for 12 weeks)	Room in a basement in Umeå, with a gray concrete floor and primrose walls	CIS ; PSQ; SMBQ; SF-36	Randomized parallel-group trial
Grazuleviciene et al. (111)	Coronary artery disease patients aged from 45 to 75 Age: 62.3 ± 12.6 Sample size (M/F): 20 (13/7)	55 ~ 69 m	Pine tree park located within a 5 min walk of the Cardiology Clinic, accessed through clinic park (54°55′04"N 23°54′54"E)	30 min walk on 7 consecutive days	Busy urban traffic road behind the Cardiology Clinic, Lithuania	SBP; DBP; HR	Randomized parallel-group trial
Han et al. (112)	Individuals with widespread chronic pain aged 25–49 Age: 31.6 ± 6.5 (exp) 37.5 ± 8.4 (con) Sample size (M/F): 61 (26/35)	280 ~ 400 m	Saneum Natural Recreation Forest in Yangpyeong county of Gyeonggi Province, Republic of Korea	Two-day forest therapy program	Normal daily routine	BDI; EQ-VAS; HRV; SDNN; TP; HR	Non-randomized controlled trial
Han (113)	University students without physical injuries, asthma, allergic reactions to sunlight, air, or plants Age: 20.85 ± 1.14 Sample size (M/F): 116 (52/64)	110 m	Forest park in National Chin-Yi University of Technology, Taiwan (24°08′56"N 120°43′47"E)	Walking or jogging for 15 min	Built road with buildings lined on all sides	POMS-SF; WMS-III	Randomized parallel-group trial
Harte and Eifert (114)	Trained runners aged from 18 to 37 Age: 27.1 Sample size (M/F): 10 (10/0)	30 m	Outdoor route around James Cook University campus with trees, Australia (19°19′32"S 146°45′22"E)	Running for 45 min	Motorized treadmill running in laboratory with brick walls and high-set windows Sitting quietly in the laboratory with a selection of sports magazines to read	POMS; attention checklist; cortisol; adrenaline; noradrenaline; SBP; DBP	Non-randomized controlled trial
Janeczko et al. (115)	Healthy young adults aged from 19 to 24 Sample size (M/F): 75	101 m	Green suburbs with trees (52°09′44"N 21°02′59"E)	Short program of walks for 30 min (2.0 km course)	Urban apartment suburbs	PANAS; POMS; ROS; SVS; SBP; DBP; pulse frequency	Non-randomized controlled trial
		100 m	Coniferous forest named Kabaty Forest, Poland (52°07′02"N 21°05′10"E)				
		118 m	Deciduous forest named Sobieski Forest, Poland (52°14′27"N 21°10′43"E)				
Lanki et al. (116)	Healthy adults aged from 30 to 60 without cardiopulmonary disease Age: 46 ± 8.7 Sample size (M/F): 36 (0/36)	11 m	Urban park named Alppipuisto, Finland (60°11′25"N 24°56′15"E)	Sedentary viewing for 15 min and walking defined road for 30 min	Built-up city center in Mannerheimintie, Finland	SBP; DBP; HRV; HF; LAeq; SDNN; RMSSD	Randomized cross-over trial
			Urban forest named Keskuspuisto, Finland (60°13′27"N 24°55′06"E)				
Lee and Lee (117)	Healthy elderly adults aged from 60 to 80 Age: 70.19 ± 4.66 (exp); 71.11 ± 5.80 (con) Sample size (M/F): 62 (0/62)	150 m	Chamaecyparis obtuse forest in Janghung, Republic of Korea	Walking at owns usual pace for 60 min	Urban area in Mokpo City, Republic of Korea	SBP; DBP	Randomized parallel-group trial
Li et al. (118)	Middle-aged male aged from 40 to 74, with high-normal or hypertension, and not taking any antihypertensive drugs Age: 51.2 ± 8.8 Sample size (M/F): 19 (19/0)	1,130 ~ 1,170 m	Forest park named Akasawa Shizen Kyuyourin in Agematsu, Nagano Prefecture, Japan (35°43′39"N 137°37′23"E)	Day trips for 80 min (twice)	Urban area of Nagano Prefecture, Japan	SBP; DBP; PR; POMS; urinary adrenaline; noradrenaline; dopamine	Non-randomized controlled trial
Liu et al. (119)	Healthy young university students aged from 22 to 28 Sample size (M/F): 30	171 m	Mixed forest, Changping, China (40°15′09"N 116°16′37"E)	Sitting for 30 min and walking for 30 min	City square in the center of the downtown area with a large amount of people and vehicles	POMS; ROS; SVS; WEMWBS; SBP; DBP; HR	Randomized cross-over trial
		203 m	Deciduous forest, Changping, China (40°15′23"N 116°16′39"E)				
		223 m	Coniferous forest, Changping, China (40°15′35"N 116°16′43"E)				
Mao et al. (120)	Elderly patients with essential hypertension aged from 60 to 75 (with no other disease) Age: 66.79 ± 3.54 (exp) 67.67 ± 4.23 (con) Sample size (M/F): 24	1,324 m	Broad-leaved evergreen forest named Zhejiang Baimashan Forest Park in Suichang County, China (28°37′09"N 119°08′52"E)	Unhurried paced walking for 90 min twice a day (7 nights trip)	Downtown area of Hangzhou, China	POMS; SBP; DBP; PP; HR	Randomized parallel-group trial
Mao Gen et al. (121)	Healthy male university students without physiological or psychiatric disorders histories Age: 20.79 ± 0.54 Sample size (M/F): 20 (20/0)	392 m	Wuchao Mountain Forest in Hangzhou, China (30°11′16"N 120°00′45"E)	Unhurried paced walking for 90 min twice a day (2 nights trip)	Downtown area of Hangzhou, China	POMS; cortisol; testosterone	Randomized parallel-group trial
Mao et al. (122)	Chronic Heart Failure patients aged from 65 to 80, without other diseases Age: 72.86 ± 5.85 Sample size (M/F): 33 (19/14)	522 m	Forest Park named Huangtan located in Pan'an County, Zhejiang Province, China (28°59′45"N 120°26′44"E)	Unhurried paced walking for 90 min twice a day (3 nights trip)	Downtown area of Hangzhou, China	POMS	Randomized parallel-group trial
Meyer et al. (123)	University students and faculty without heart problems aged from 19 to 69 Sample size (M/F): 18 (18/0)	431 m	Forest trail located in Göttingen, Germany (51°32′05"N 10°03′09"E)	Unhurried paced walking for 90 min twice a day (7 nights trip)	Traffic road located in Göttingen, Germany	POMS; HRV (HF, LF/HF); EDA	Randomized cross-over trial
Morita et al. (124)	Healthy male and female volunteers aged 20 or more Age: 56.2 ± 10.6 Sample size (M/F): 498 (244/254)	90 ~ 220 m	University of Tokyo Chiba Forest, Japan	Walking for 140 min	Exercise or take part in their favorite activities, except visiting forest	MMS-SF; STAI	Non-randomized controlled trial
Song et al. (125)	Healthy young male adults Age: 21.2 ± 1.7 Sample size (M/F): 17 (17/0)	20 ~ 30 m	Urban park named Kashiwa-no-ha Park, Chiba Prefecture, Japan (start point: 35°53′34"N 139°56′34"E End point: 35°53′44"N 139°56′25"E)	Walking for 15 min	Built road nearby residential area	POMS; STAI; HRV	Non-randomized controlled trial
Song et al. (126)	Healthy young male university students Age: 22.3 ± 1.2 Sample size (M/F): 23 (23/0)	20 ~ 30 m	Urban park named Kashiwa-no-ha Park, Chiba Prefecture, Japan (start point: 35°53′34"N 139°56′34"E End point: 35°53′44"N 139°56′25"E)	Walking for 15 min	Built road nearby residential area	POMS; STAI; HRV [HF, ln (LF/HF)]	Non-randomized controlled trial
Song et al. (127)	Middle-aged hypertensive male without taking medication for chronic conditions Age: 58.0 ± 10.6 Sample size (M/F): 20 (20/0)	1,120 ~ 1,168 m	Akasawa natural recreation forest located in Agematsu town of Nagano Prefecture, Japan (35°43′39"N 137°37′23"E)	Walking for 17 min	Urban site located in a City of Nagano Prefecture, Japan	POMS; HRV [HF, ln (LF/HF)]	Randomized cross-over trial
Sung et al. (128)	Patients with stage 1 hypertension Age: 66 ± 7 (exp) 63 ± 11 (con) Sample size (M/F): 56 (22/34)	280 ~ 400 m; 850 ~ 1,000 m	Two recreation forest sites, Hoengseong and Saneum, in Kangwon-do, Republic of Korea	Guided activity 3 day-program in the forest	Self-monitoring of BP for 8 weeks without participating in the program	SBP; DBP; salivary cortisol	Non-randomized controlled trial
Tyrväinen et al. (65)	Healthy, non-smoking adults whose place of work was in the Helsinki Metropolitan Area Age: 47.64 ± 8.68 Sample size (M/F): 77 (6/71)	11 m	Urban park named Alppipuisto, Finland (60°11′25"N 24°56′15"E)	Viewing for 15 and 30 min walk led by a researcher	Built-up city center next to the main street with few single urban trees	PANAS; PRS; ROS; SVS; TFOAS; salivary cortisol	Randomized cross-over trial
		14 ~ 49 m	Urban forest named Keskuspuisto, Finland (60°13′32"N 24°55′00"E)				
Wang et al. (129)	Chinese undergraduate students aged from 18 to 21 Age: 19.1 ± 0.7 Sample size (M/F): 77 (32/45)	15 m	Crescent Lake Park, Qinhuai District, Nanjing, China (32° 02′ 01.3′′N, 118° 49′ 40.5′′E)	1.6 km walk	The gym setting which was located on the second floor of the sports center of the university	BFS; SBP; DBP; HR	Randomized parallel-group trial
Zeng et al. (130)	Healthy university students without physiological or psychiatric disorders in personal histories Age: 21.46 ± 0.39 Sample size (M/F): 120 (60/60)	634 m	Bamboo forest located near the city of Ya′an (28°28′22"N, 105°0′19"E)	Viewing for 15 min Walking for 15 min (2 nights 3 days program)	Urban settings located in Chengdu, China	SBP; DBP; HR; SpO2	Randomized parallel-group trial
		754 m	Bamboo forest located near the city of Dujiangyan (31°44′54"N, 103°25′42"E)				
		892 m	Bamboo forest located near the city of Yibin (28°28′22"N, 105°0′19"E)				

ANT, attention network test; BCT, breath-counting test; BDI, beck depression inventory; BDS, backward digit span; BFS, Befindlichkeitsskalen mood states scale; CIS, The Checklist Individual Strength; DBP, diastolic blood pressure; EDA, electrodermal activity; EQ-VAS, EuroQol visual analog scale on health-related quality of life; HAM-D17, 17-item version of the Hamilton depression rating scale; HR, heart rate; HRV, heart rate variability; LAeq, a-weighted equivalent continuous sound pressure level; ln(LF/HF), the natural logarithm of LF/HF; MMS-SF, multiple mood scale-short form; OSPAN, operation span task; PANAS, positive and negative affect schedule; POMS, profile of mood states; POMS-SF, profile of mood state short form; PR, pulse rate; PSQ, perceived stress questionnaire; PSS, perceived stress scale; RMSSD, square root of the mean of the sum of the squares of differences between adjacent normal-to-normal intervals; ROS, restorative outcomes scale; RRQ, rumination-reflection questionnaire; SBP, systolic blood pressure; SDNN, standard deviation of normal to normal intervals; SF-36, short form 36 survey; SMBQ, Shirom-Melamed burnout questionnaire; SpO2, oxygen saturation; STAI, Spielberger state-trait anxiety inventory; SVS, subjective vitality scale; TFOAS, focus of attention scale; TP, total power; WEMWBS, Warwick-Edinburgh mental wellbeing scale; WMS-III, Wechsler memory scale, third edition; atm, atmospheric pressure.

Most of the included studies were randomized trials that employed either a randomized parallel-group design (*N* = 13) or a randomized cross-over design (*N* = 5), whereas the others were non-randomized controlled trials (*N* = 9). For studies with double or triple arms (65, 115, 116, 119), each arm was included as one independent case in comparison with the control. For studies reporting results separately by type or time of activity performed in both forest and control, each activity-specific case was included (116, 118, 119). Consequently, the studies will appear multiple times in the graphics and tables. The included studies cover a total of 1,668 participants, all of whom were adults. Participants totaled 436 below 100 m, 783 between 100 and 200 m, 153 between 200 and 500 m, 268 between 500 and 1,000 m, and 63 over 1,000 m. Most were healthy adults with no current or past diagnoses (*N* = 19). Hypertensive adults without medications for other conditions were recruited for four studies (118, 120, 127, 128). In addition, four included studies recruited patients with chronic heart failure (122), chronic stroke (107), coronary artery disease (111), and widespread chronic pain (112).

The included studies reported quantitative outcomes for emotional restoration (*N* = 19), physiological relaxation (*N* = 19), cognitive restoration (*N* = 7), and stress reduction (*N* = 7). Quantitative synthesis was performed on the results identified in more than ten cases. Mood states (including anxiety, depression, confusion, fatigue, hostility, and vitality) and blood pressure were investigated as an outcome in 20 and 12 studies, respectively. Cognitive restoration experience and heart rate or pulse rate were investigated in four and seven studies, respectively, although these reported in more than 10 cases. Affective state valance (65, 105, 106, 115), cognitive task score (65, 106, 113, 114), perceived stress level (109, 110) stress hormones (65, 114, 118, 121, 128), oxygen saturation (130), and time-domain measures or frequency-domain measures in heart rate variability (108, 112, 116, 123, 125–127) could not be analyzed owing to insufficient observations.

Prior to the quantitative synthesis, the investigators (EK, GK, SP, SK, and YC) reviewed several study-level variables to rule out possible confounding factors; namely, participant characteristics, geographical coordinates, climate classification, species compositions, physical environment of forest settings, time of measurement, duration, frequency, and activity intensity of interventions. Consequently, 31 cases from 19 studies were meta-analyzed. Seven studies were excluded because of unattainable data types (121, 125–127), rarely observed outcome (109), different species composition (130), and different climatic conditions (114).

### Methodological quality

Results of the methodological quality assessment are presented in Tables 3, 4 for randomized trials and non-randomized trials, respectively. More than half of the included studies rated the risk of bias as “moderate” or “some concern.” Among the randomized trials, one study was deemed as “low” risk (129), 10 as being of “some concern” (38, 105, 106, 109, 111, 116, 117, 119, 120, 130), and seven as “high” risk of bias (65, 107, 110, 113, 121, 122, 127). Among the non-randomized trials, one study was deemed as “low” risk (108), five as “moderate” (112, 114, 118, 124, 128), and three as “serious” risk of bias (115, 125, 126).

**Table 3:** Methodological quality assessment of randomized studies using RoB2 tool.

**Study**		**D1**	**DS**	**D2**	**D3**	**D4**	**D5**	**Overall risk of bias**
**First author**	**Year**	**Randomization process**	**Period and carryover effects**	**Deviations from the intended interventions**	**Missing outcome data**	**Measurement of the outcome**	**Selection of the reported result**	
**Randomized parallel-group trial**								
Bielinis et al. (105)	2019	Some concern	–	Low risk	Low risk	Low risk	Low risk	Some concern
Bratman et al. (106)	2015	Some concern	–	Some concern	Low risk	Low risk	Some concern	Some concern
Chun et al. (107)	2017	Low risk	**–**	Some concern	Low risk	High risk	Some concern	High risk
Djernis et al. (109)	2021	Low risk	**–**	Low risk	Low risk	Some concern	Low risk	Some concern
Dolling et al. (110)	2017	Some concern	–	Some concern	Some concern	High risk	Some concern	High risk
Grazuleviciene et al. (111)	2015	Some concern	–	Low risk	Low risk	Low risk	Some concern	Some concern
Han (113)	2017	Some concern	–	Low risk	Low risk	High risk	High risk	High risk
Lee and Lee (117)	2014	Low risk	–	Some concern	Some concern	Low risk	Low risk	Some concern
Mao et al. (120)	2012	Some concern	–	Some concern	Some concern	Some concern	Some concern	Some concern
Mao Gen et al. (121)	2012	Some concern	–	Some concern	Some concern	Some concern	High risk	High risk
Mao et al. (122)	2016	Some concern	–	Low risk	Some concern	High risk	Low risk	High risk
Wang et al. (129)	2021	Low risk	–	Low risk	Low risk	Low risk	Low risk	Low risk
Zeng et al. (130)	2020	Some concern	–	Low risk	Low risk	Low risk	Some concern	Some concern
**Randomized cross-over trial**								
Lanki et al. (116)	2017	Some concern	Some concern	Some concern	Low risk	Low risk	Low risk	Some concern
Liu et al. (119)	2021	Some concern	Some concern	Low risk	Low risk	Low risk	Low risk	Some concern
Meyer et al. (123)	2016	Some concern	Some concern	Low risk	Low risk	Some concern	Some concern	Some concern
Song et al. (127)	2015	High risk	Some concern	Some concern	Low risk	Some concern	High risk	High risk
Tyrväine et al. (65)	2014	Some concern	Low risk	Some concern	Some concern	High risk	Some concern	High risk

**Table 4:** Methodological quality assessment of non-randomized studies using ROBINS-I tool.

**Study**		**Pre-intervention**		**At intervention**	**Post-intervention**				**Overall risk of bias**
**First author**	**Year**	**Bias due to confounding**	**Bias in selection of participants into the study**	**Bias in classification of interventions**	**Bias due to deviations from intended interventions**	**Bias due to missing data**	**Bias in measurement of outcome**	**Bias in selection of the reported result**	
de Brito et al. (108)	2020	Low	Low	Low	Low	Low	Low	Low	Low
Han et al. (112)	2016	Moderate	Low	Low	Low	Low	Moderate	Low	Moderate
Harte and Eifert (114)	1995	Moderate	Low	Low	Low	Low	Low	Moderate	Moderate
Janeczko et al. (115)	2020	Serious	Low	Low	No information	No information	Serious	Moderate	Serious
Li et al. (118)	2016	Moderate	Low	Low	Low	Low	Moderate	Moderate	Moderate
Morita et al. (124)	2007	Moderate	Low	Low	Low	Low	Serious	Low	Moderate
Song et al. (125)	2014	Moderate	Low	Low	Low	Low	Moderate	Serious	Serious
Song et al. (126)	2015	Moderate	Low	Low	Low	Low	Moderate	Serious	Serious
Sung et al. (128)	2012	Moderate	Low	Low	Low	Low	Low	Low	Moderate

In randomized trials, the randomization process (D1), measurement of the outcome (D4), and selection of reported result (D5) were principally responsible for raising overall risk of bias. Only four studies described a detailed randomization process and allocation concealment (107, 109, 117, 129); however, the remaining studies were not sufficiently detailed. The majority of studies identified baseline differences for assignments, with one exception (127). Since 10 studies used self-reported measures without adequate participant blinding, the risk of bias in measurement was rated as “some concern” or “high risk” (65, 107, 109, 110, 113, 120–123, 127). Regarding reporting, 15 studies fully disclosed the results of multiple outcome measures and multiple analyses; seven studies were rated as having “low” risk of bias as they had pre-specified plans (105, 109, 116, 117, 119, 122, 129), whereas eight were rated as having “some concerns” owing to the lack of evidence to justify their analytical methods (65, 106, 107, 110, 111, 120, 123, 130).

In non-randomized trials, confounding bias and bias in the measurement of outcomes and selection of reported results were generally responsible for increasing overall risk of bias. Most studies were adequately measured or controlled for significant confounding factors; however, these were not comparable to well-performed randomizations, and therefore, were rated as “moderate” risk of bias. Eight studies using self-reported measures without adequate participant blinding were rated as “moderate” to “high” risk of bias, because knowledge of the intervention could lead to errors in measurement (112, 114, 115, 118, 124–126, 128). With the exception of two studies (125, 126), the majority provided complete disclosure of the measures and analyses (114, 115, 118), and four studies even offered pre-specified plans supporting their analytical methods (108, 112, 124, 128).

### Quantitative synthesis

#### Overall effects of forest exposure

Compared with non-nature exposure, forest exposure revealed significant alleviating effects on symptoms of anxiety, depression, confusion, fatigue, and hostility with effect size (SMD) as follows: SMD = −1.20 (95% CI: −1.50–−0.89, 18 cases); SMD = −1.01 (95% CI: −1.34–−0.67, 17 cases); SMD = −1.05 (95% CI: −1.34–−0.75, 14 cases), −0.77(95% CI: −1.05–−0.49, 17 cases); and SMD = −0.77 (95% CI: −1.05–−0.49, 14 cases), respectively. Similarly, significant lowering in diastolic blood pressure (SMD = −0.32, 95% CI: −0.55–−0.10, 21 cases), systolic blood pressure (SMD = −0.50, 95% CI: −0.77–−0.23, 21 cases), and heart rate (SMD = −0.80, 95% CI: −1.09–−0.51, 23 cases) were observed. Additionally, Significant improvements in vitality (SMD = 1.04, 95% CI: 0.58–1.50, 14 cases) and restorative experience (SMD = 1.38, 95% CI: 0.95–1.80, 14 cases) were observed. Overall effect size and heterogeneity are reported in Table 5. Forest plots are reported in the Supplemental Figures 1–10.

**Table 5:** Overall effect size and test of heterogeneity (random effects model).

	**Altitude range**	**Cases combined**	**Total effect size**				**Total heterogeneity**			
			**SMD**	**95% CI**	***z***	***p*** **-value**	***I^2^ %***	**95% CI**	**Cochran Q**	***p*** **-value**
Anxiety***	90–1,324 m	17	−1.1956	[−1.5044–0.8869]	−7.59	< 0.000	82.3	[72.8% 88.5%]	90.61	< 0.000
Depression***	100–1,324 m	17	−1.0054	[−1.3418–0.6690]	−5.86	< 0.000	86.3	[79.6% 90.8%]	117.11	<0.000
Confusion***	100–1,324 m	14	−1.0463	[−1.3425–0.7502]	−6.92	<0.000	66.6	[41.5% 81.0%]	38.97	0.0002
Fatigue***	90–1,324 m	16	−0.7717	[−1.1460–0.3973]	−4.04	<0.000	82.1	[72.0% 88.5%]	83.66	<0.000
Hostility***	100–1,324 m	14	−0.7688	[−1.0521–0.4854]	−5.32	<0.000	76.9	[61.5% 86.2%]	56.34	<0.000
Vitality***	11–1,150 m	14	1.0390	[0.5804 1.4975]	4.44	<0.000	89.3	[83.8% 92.9%]	121.34	<0.000
ROS***	11–223 m	14	1.3773	[0.9549 1.7996]	6.39	<0.000	89.7	[84.5% 93.2%]	126.12	<0.000
DBP**	11–1,324 m	21	−0.3242	[−0.5515–0.0970]	−2.80	0.0052	71.2	[55.2% 81.4%]	69.38	<0.000
SBP***	11–1,324 m	21	−0.4982	[−0.7678–0.2287]	−3.62	0.0003	79.0	[68.6% 86.0%]	95.32	<0.000
HR***	11–1,324 m	23	−0.8010	[−1.0935–0.5086]	−5.37	<0.000	82.0	[73.9% 87.5%]	121.92	<0.000

Significance codes: 0 “^***^”; 0.001 “^**^”; 0.10“ ”.

#### Meta-regression

A series of meta-regressions detected a non-linear association between altitude studies and effect sizes. The model comparison results are presented in Table 6. In the log-likelihood ratio test, the quadratic model offered a significantly better fit for anxiety (Chi^2^ = 5.753, *p* = 0.016), depression (Chi^2^ = 9.040, *p* = 0.003), and confusion (Chi^2^ = 4.180, *p* = 0.041). Similarly, in AIC model selection, quadratic models for anxiety, depression, and confusion carried 62, 83, and 47%, respectively, of predictive power provided by the full set of models. Regarding physiological relaxation, a cubic association was noted for diastolic blood pressure (Chi^2^ = 6.447, *p* = 0.011, AICc weight = 44%), systolic blood pressure (Chi^2^ = 3.731, *p* = 0.005, AICc weight = 17%), and heart rate (Chi^2^ = 7.239, *p* = 0.007, AICc weight = 65%). Fitted meta-regression plots and test statistics are presented in Table 7.

**Table 6:** Comparison of the models in terms of log likelihoods and information criteria.

	**logLik**			**Chi^2^**	***p*** **-value**	**AICc (weight)**		
	**Linear**	**Quadratic**	**Cubic**			**Linear**	**Quadratic**	**Cubic**
Anxiety	−17.237	−14.360*	−13.511	5.7528	0.0164	42.32 (20%)	**40.05 (62%)**	42.48 (18%)
Depression	−18.800	−14.280**	−14.143	9.0403	0.0026	45.45 (5%)	**39.89 (83%)**	43.74 (12%)
Confusion	−10.849	−8.760*	−7.978	4.1795	0.0409	30.10 (44%)	**29.96 (47%)**	33.46 (8%)
Fatigue	−18.520	−18.162	−17.607	n. s.	n. s.	45.04 (78%)	47.96 (18%)	51.21 (4%)
Hostility	−3.4316	−3.3634	−3.1629	n. s.	n. s.	15.26 (87%)	19.17 (12%)	23.83 (1%)
Vitality	−18.122	−17.655	−15.980	n. s.	n. s.	44.64 (77%)	47.75 (16%)	49.46 (7%)
ROS	−13.924	−11.436*	−7.755**	7.3618	0.0067	33.01 (13%)	**35.32 (21%)**	**33.01 (66%)**
DBP	−20.042	−20.014	−16.790*	6.4470	0.0111	**47.50 (46%)**	50.53 (10%)	**47.58 (44%)**
SBP	−22.255	−22.243	**−20.377** ^**•**^	3.7312	0.0534	**51.92 (69%)**	54.99 (15%)	54.75 (17%)
HR	−29.715	−29.256	−25.636**	7.2394	0.0071	66.69 (25%)	68.73 (9%)	**64.80 (65%)**

logLik, log-likelihood of the model evaluated at the estimated coefficients; AICc, corrected Akaike information criterion for small sample sizes; A higher logLik indicates a better fit of the model; A lower information criterion indicates a better balance between increased fit and increased model complexity; significance codes: 0.001 “^**^”; 0.01 “^*^”; 0.05 “•”; 0.10 “ ”. Statistically significant results are in bold.

**Table 7:** Fitted meta-regression plots for three different models.

	**Anxiety**				**Depression**				**Confusion**				**Fatigue**				**Hostility**			
Linear model (unit: 10 m)	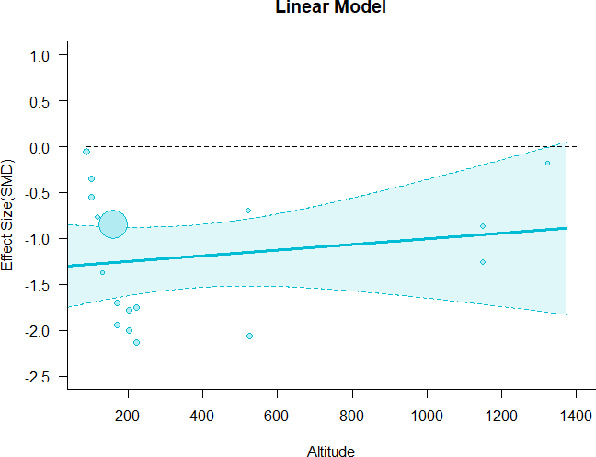 ***I${}_{T}^{2}$*** = 82.3%, ***I${}_{R}^{2}$*** = 85.8%, ***R^**2**^*** = 0.00% Q_**M**_ = 0.477 (***p*** = 0.490), Q_**E**_ = 91.848 (***p*** < 0.000)				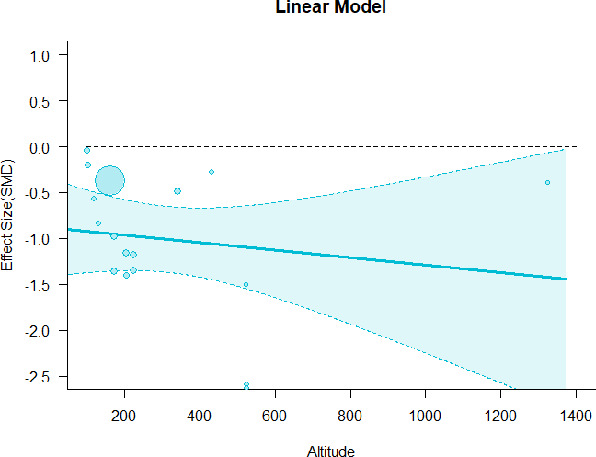 ***I${}_{T}^{2}$*** = 86.3%, ***I${}_{R}^{2}$*** = 88.0%, ***R^**2**^*** = 0.00% Q_**M**_ = 0.384 (***p*** = 0.535), Q_**E**_ = 105.251 (***p*** < 0.000)				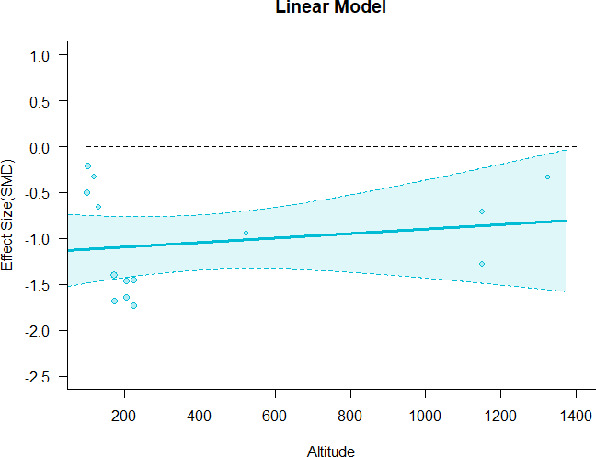 ***I${}_{T}^{2}$*** = 66.6%, ***I${}_{R}^{2}$*** = 68.4%, ***R^**2**^*** = 0.00% Q_**M**_ = 0.445 (***p*** = 0.505), Q_**E**_ = 38.107 (***p*** < 0.000)				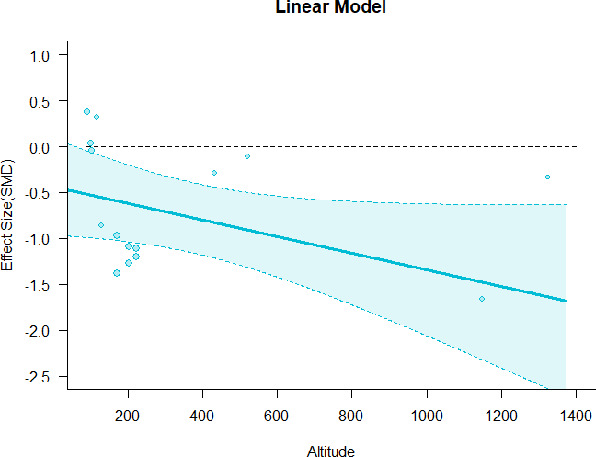 ***I${}_{T}^{2}$*** = 82.1%, ***I${}_{R}^{2}$*** = 83.3%, ***R^**2**^*** = 14.77% Q_**M**_ = 3.322 (***p*** = 0.068), Q_**E**_ = 74.420 (***p*** < 0.000)				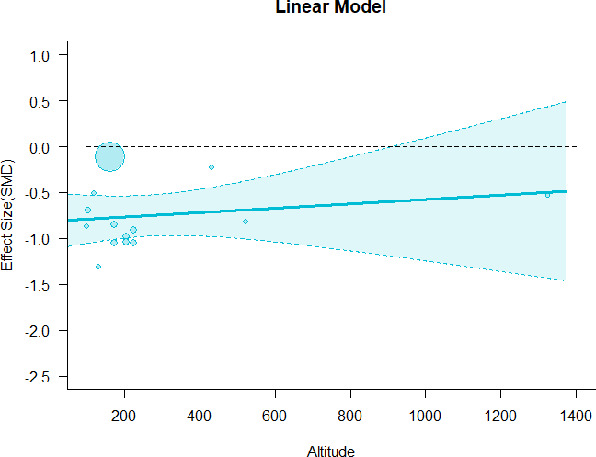 ***I${}_{T}^{2}$*** = 76.9%, ***I${}_{R}^{2}$*** = 62.03%, ***R^**2**^*** = 0.00% Q_**M**_ = 0.310 (***p*** = 0.578), Q_**E**_ = 55.934 (***p*** < 0.000)			
		**β**	**SE**	**95% CI**		**β**	**SE**	**95% CI**		**β**	**SE**	**95% CI**		**β**	**SE**	**95% CI**		**β**	**SE**	**95% CI**
	Int***	−1.3120	0.2407	[−1.7839 −0.8402]	Int**	−0.8826	0.2740	[−1.4196 −0.3456]	Int***	−1.1433	0.2114	[−1.5576 −0.7290]	Int	−0.4379	0.2691	[−0.9654 0.0896]	Int***	−8.148	0.1602	[−1.1288 −0.5008]
	Alt	0.0031	0.0045	[−0.0057 0.0118]	Alt	−0.0041	0.0066	[−0.0170 0.0088]	Alt	0.0025	0.0037	[−0.0048 0.0097]	Alt^•^	−0.0091	0.0050	[−0.0188 0.0007]	Alt	0.0024	0.0044	[−0.0061 0.0110]
Quadratic model (unit: 10 m)	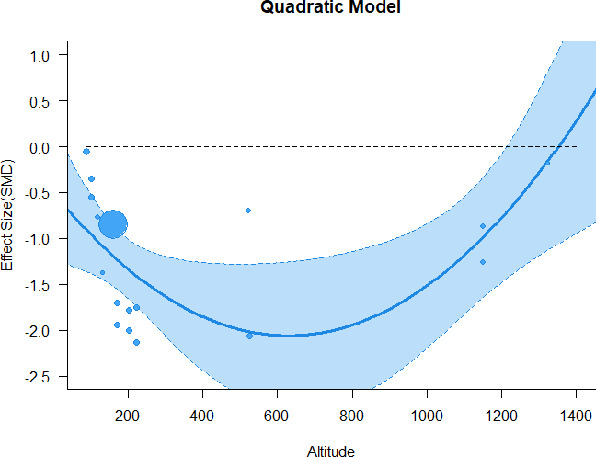 ***I${}_{T}^{2}$*** = 82.3%, ***I${}_{R}^{2}$*** = 80.1%, ***R^**2**^*** = 31.41% Q${}_{\text{M}}^{•}$= 6.851 (***p*** = 0.033), Q_**E**_ = 60.167 (***p*** < 0.000)				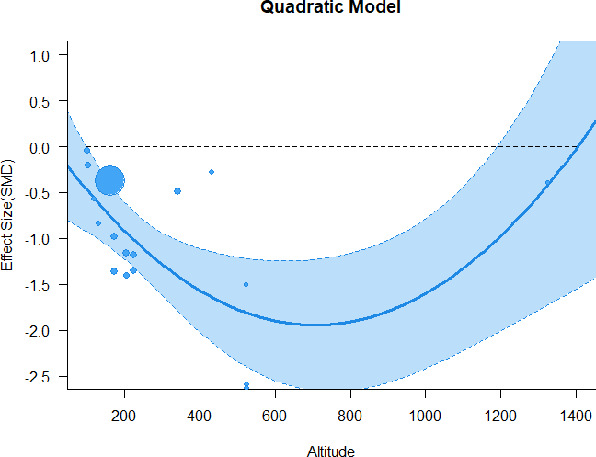 ***I${}_{T}^{2}$*** = 86.3%, ***I${}_{R}^{2}$*** = 81.2%, ***R^**2**^*** = 39.41% Q${}_{\text{M}}^{*}$= 9.732 (***p*** = 0.008), Q_**E**_ = 66.369 (***p*** < 0.000)				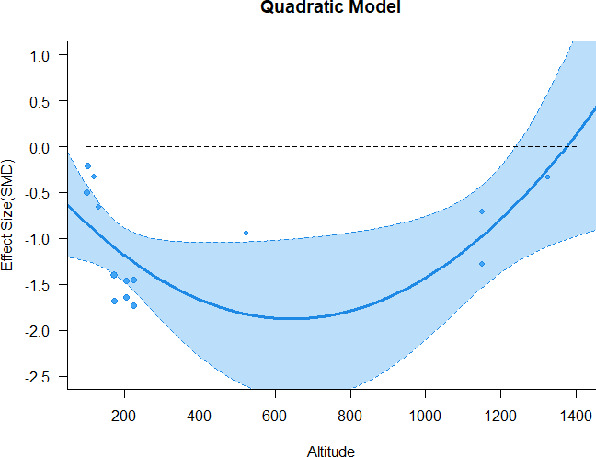 ***I${}_{T}^{2}$*** = 66.6%, ***I${}_{R}^{2}$*** = 58.8%, ***R^**2**^*** = 31.10% Q_**M**_ = 4.925 (***p*** = 0.085), Q_**E**_ = 26.315 (***p*** = 0.006)				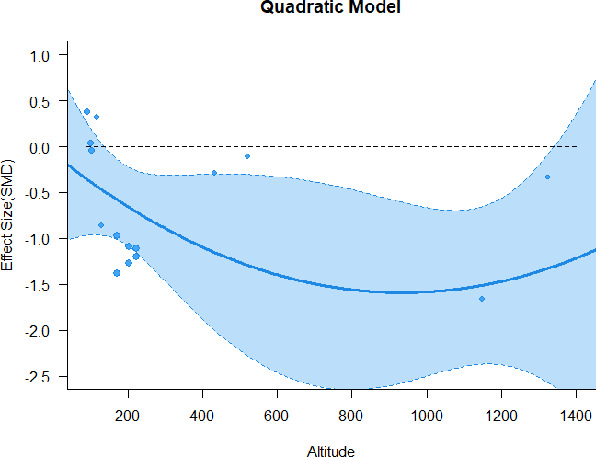 ***I${}_{T}^{2}$*** = 82.1%, ***I${}_{R}^{2}$*** = 83.9%, ***R^**2**^*** = 11.95% Q_**M**_ = 3.888 (***p*** = 0.143), Q_**E**_ = 69.994 (***p*** < 0.000)				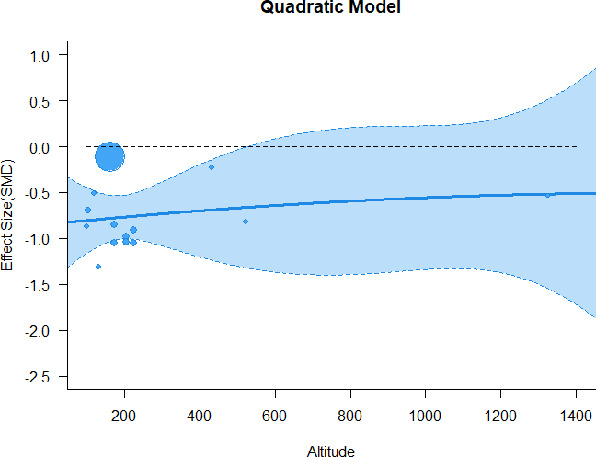 ***I${}_{T}^{2}$*** = 76.9%, ***I${}_{R}^{2}$*** = 64.34%, ***R^**2**^*** = 0.00% Q_**M**_ = 0.325 (***p*** = 0.850), Q_**E**_ = 54.559 (***p*** < 0.000)			
		**β**	**SE**	**95% CI**		**β**	**SE**	**95% CI**		**β**	**SE**	**95% CI**		**β**	**SE**	**95% CI**		**β**	**SE**	**95% CI**
	Int	−0.5073	0.3782	[−1.2485 0.2340]	Int	0.0464	0.3759	[−0.6904 0.7832]	Int	−0.4298	0.3902	[−1.1945 0.3349]	Int	−0.0886	0.5142	[−1.0964 0.9191]	Int**	−0.8530	0.3287	[−1.4973 −0.2088]
	Alt*	−0.0493	0.0212	[−0.0908 −0.0077]	Alt**	−0.0564	0.0181	[−0.0918 −0.0209]	Alt^**•**^	−0.0449	0.0229	[−0.0898 0.0000]	Alt	−0.0316	0.0286	[−0.0877 0.0245]	Alt	0.0047	0.0185	[−0.0316 0.0409]
	Alt^2*^	0.0004	0.0002	[0.0001 0.0007]	Alt^2**^	0.0004	0.0001	[0.0001 0.0007]	ALT^2*^	0.0003	0.0002	[0.0000 0.0007]	Alt^2^	0.0002	0.0002	[−0.0002 0.0006]	Alt^2^	−0.0000	0.0001	[−0.0003 0.0002]
Cubic model (unit: 10 m)	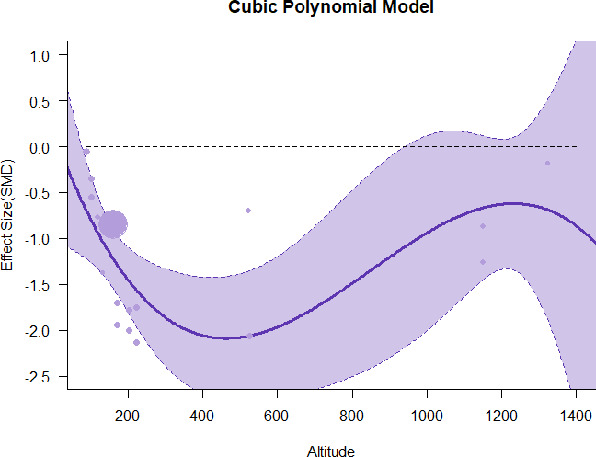 ***I${}_{T}^{2}$*** = 82.3%, ***I${}_{R}^{2}$*** = 78.9%, ***R^**2**^*** = 37.79% Q${}_{\text{M}}^{*}$ = 9.232 (***p*** = 0.023), Q_**E**_ = 53.774 (***p*** < 0.000)				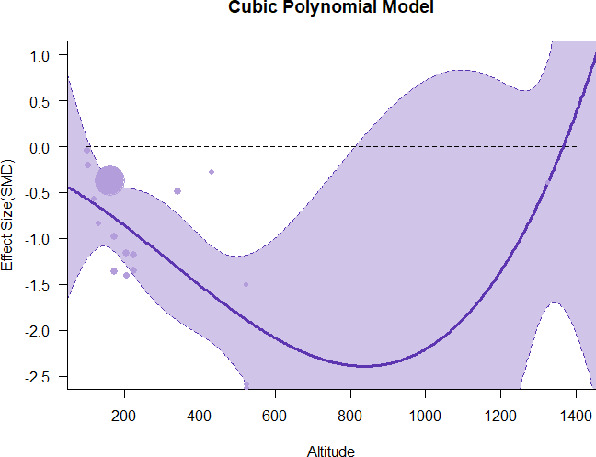 ***I${}_{T}^{2}$*** = 86.3%, ***I${}_{R}^{2}$*** = 82.6%, ***R^**2**^*** = 34.30% Q${}_{\text{M}}^{*}$ = 9.392 (***p*** = 0.025), Q_**E**_ = 65.631 (***p*** < 0.000)				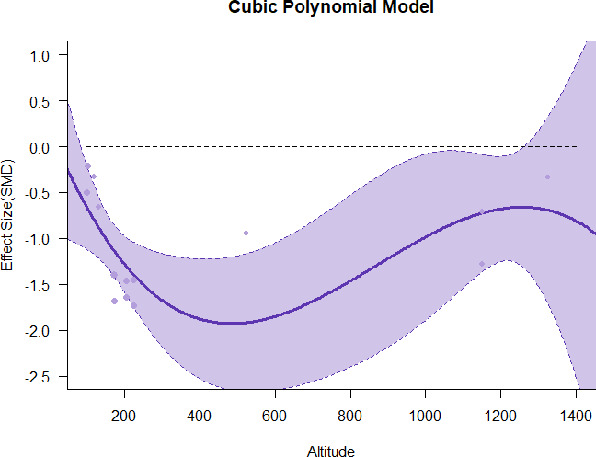 ***I${}_{T}^{2}$*** = 66.6%, ***I${}_{R}^{2}$*** = 54.0%, ***R^**2**^*** = 44.39% Q_**M**_ = 7.410 (***p*** = 0.0599), Q_**E**_ = 21.539 (***p*** < 0.000)				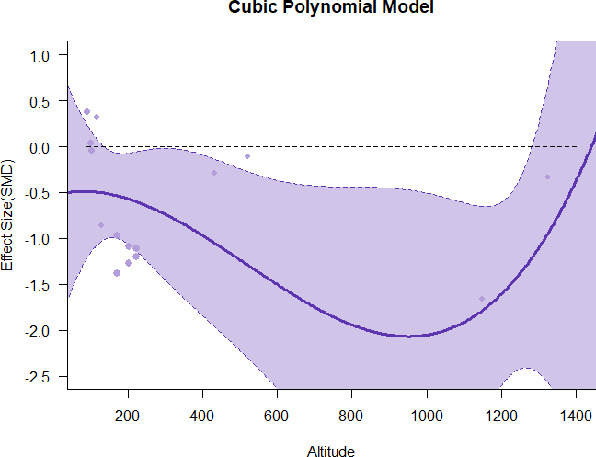 ***I${}_{T}^{2}$*** = 82.1%, ***I${}_{R}^{2}$*** = 85.1%, ***R^**2**^*** = 5.60% Q_**M**_ = 3.888 (***p*** = 3.888), Q_**E**_ = 69.994 (***p*** <0.000)				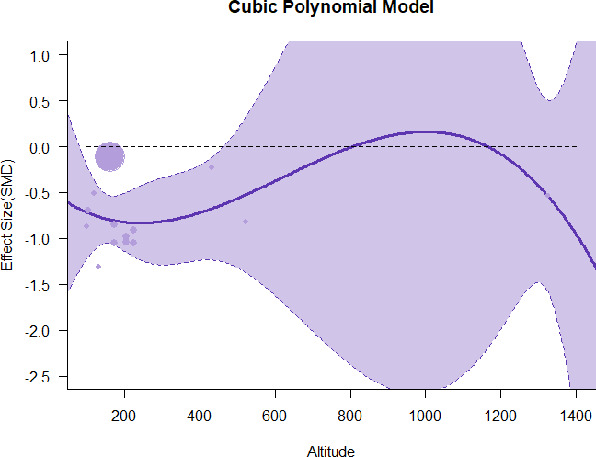 ***I${}_{T}^{2}$*** = 76.9%, ***I${}_{R}^{2}$*** = 66.65%, ***R^**2**^*** = 0.00% Q_**M**_ = 0.562 (***p*** = 0.905), Q_**E**_ = 54.419 (***p*** < 0.000)			
		**β**	**SE**	**95% CI**		**β**	**SE**	**95% CI**		**β**	**SE**	**95% CI**		**β**	**SE**	**95% CI**		**β**	**SE**	**95% CI**
	Int	0.1476	0.6061	[−1.0403 1.3356]	Int	−0.3636	1.0063	[−2.3360 1.6088]	Int	0.1896	0.5859	[−0.9599 1.3380]	Int	−0.5558	0.8185	[−2.1600 1.0485]	Int	−0.4889	0.8283	[−2.1124 1.1346]
	Alt*	−0.1109	0.0501	[−0.2091 −0.0126]	Alt	−0.0176	0.0899	[−0.1938 0.1586]	Alt*	−0.1006	0.0463	[−0.1913 −0.0099]	Alt	0.0129	0.0663	[−0.1171 0.1430]	Alt	−0.0311	0.0762	[−0.1805 0.1182]
	Alt^2•^	0.0017	0.0010	[−0.0002 0.0035]	Alt^2^	−0.0005	0.0019	[−0.0042 0.0033]	Alt^2•^	0.0014	0.0008	[−0.0002 0.0031]	Alt^2^	−0.0008	0.0013	[−0.0033 0.0017]	Alt^2^	0.0008	0.0017	[−0.0025 0.0042]
	Alt^3^	−0.0000	0.0000	[−0.0000 0.0000]	Alt^3^	0.0000	0.0000	[−0.0000 0.0000]	Alt^3^	−0.0000	0.0000	[−0.0000 0.0000]	Alt^3^	0.0000	0.0000	[−0.0000 −0.0000]	Alt^3^	−0.0000	0.0000	[−0.0000 0.0000]
	**Vitality**				**ROS**				**DBP**				**SBP**				**HR**			
Linear model (unit: 10 m)	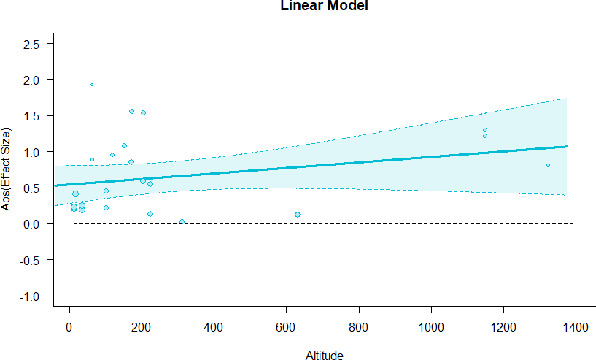 ***I${}_{T}^{2}$*** = 91.5%, ***I${}_{R}^{2}$*** =93.8%, ***R^**2**^*** = 0.00% Q_**M**_ = 0.681(***p*** = 0.409), Q_**E**_ = 149.885 (***p*** < 0.000)				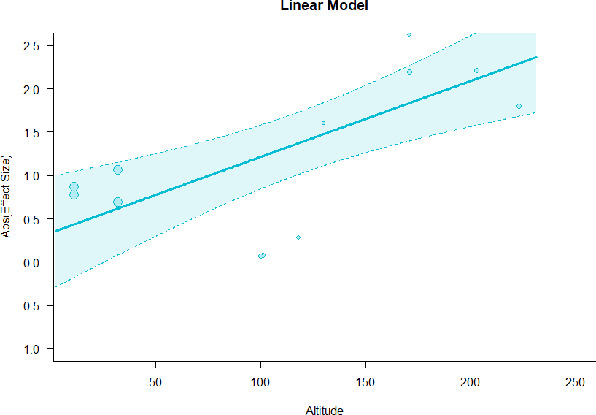 ***I${}_{T}^{2}$*** = 89.7%, ***I${}_{R}^{2}$*** =85.9%, ***R^**2**^*** = 54.01% Q${}_{\text{M}}^{***}$= 13.231 (***p*** = 0.000), Q_**E**_ = 62.708 (***p*** < 0.000)				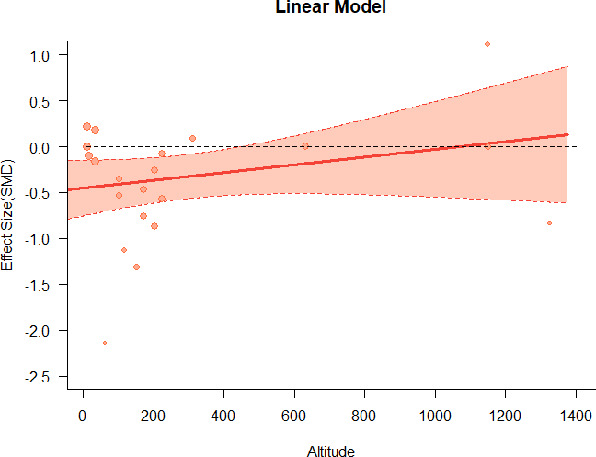 ***I${}_{T}^{2}$*** = 71.2%, ***I${}_{R}^{2}$*** = 74.8%, ***R^**2**^*** = 2.92% Q_**M**_ = 1.6453 (***p*** = 0.1996), Q_**E**_ = 71.242 (***p*** < 0.000)				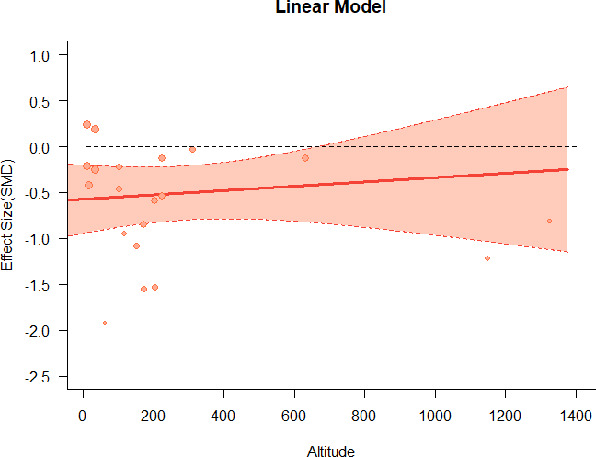 ***I${}_{T}^{2}$*** = 79.0%, ***I${}_{R}^{2}$*** = 82.9%, ***R^**2**^*** = 0.00% Q_**M**_ = 0.3503 (***p*** = 0.5540), Q_**E**_ = 96.5741 (***p*** < 0.000)				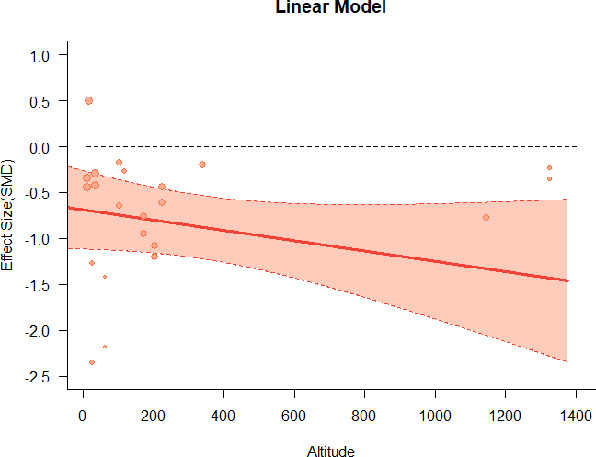 ***I${}_{T}^{2}$*** = 82.0%, ***I${}_{R}^{2}$*** = 86.9%, ***R^**2**^*** = 7.59% Q_**M**_ = 2.6495 (***p*** = 0.1036), Q_**E**_ = 114.6336 (***p*** < 0.000)			
		**β**	**SE**	**95% CI**		**β**	**SE**	**95% CI**		**β**	**SE**	**95% CI**		**β**	**SE**	**95% CI**		**β**	**SE**	**95% CI**
	Int**	1.0150	0.3894	[0.2518 1.7781]	Int	0.3501	0.3354	[−0.3073 1.0076]	Int**	−0.4512	0.1546	[−0.7543 −0.1482]	Int**	−0.5726	0.1896	[−0.9442 −0.2011]	Int**	−0.6143	0.2150	[−1.0357 −0.1928]
	Alt	0.0071	0.0087	[−0.0098 0.0241]	Alt***	0.0875	0.0241	[0.0404 0.1347]	Alt	0.0042	0.0033	[−0.0022 0.0107]	Alt	0.0024	0.0040	[−0.0055 0.0102]	Alt	−0.0063	0.0039	[−0.0139 0.0013]
Quadratic model (unit: 10 m)	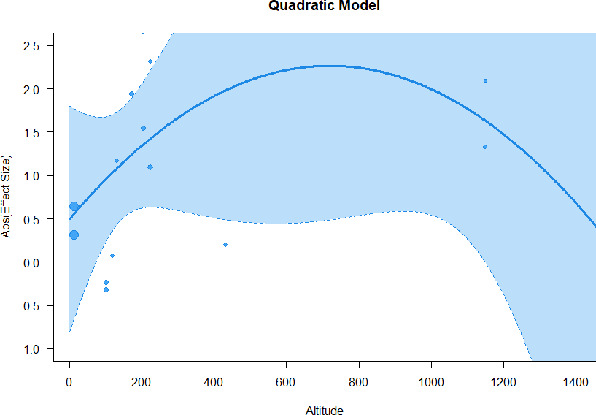 ***I${}_{T}^{2}$*** = 91.5%, ***I${}_{R}^{2}$*** = 93.6%, ***R^**2**^*** = 0.00% Q_**M**_ = 1.548 (***p*** = 0.461), Q_**E**_ = 133.329 (***p*** < 0.000)				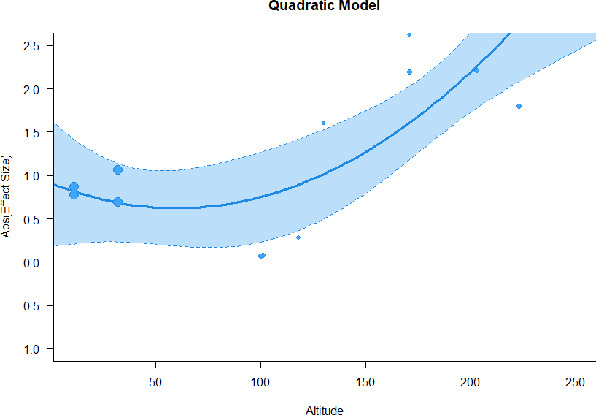 ***I${}_{T}^{2}$*** = 89.7%, ***I${}_{R}^{2}$*** = 77.9%, ***R^**2**^*** = 70.15% Q_**M**_ = 23.644 (***p*** < 0.000), Q_**E**_ = 41.425 (***p*** < 0.000)				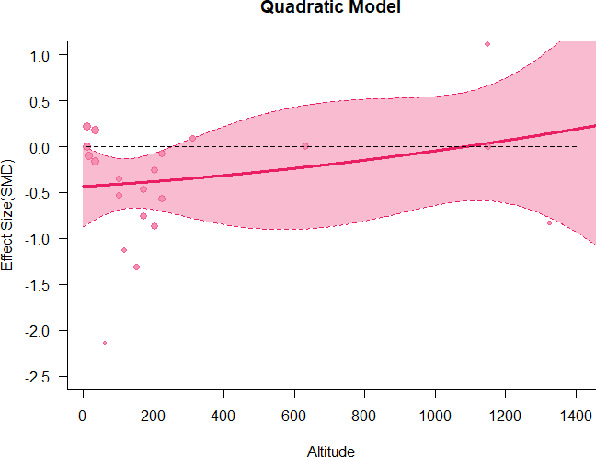 ***I${}_{T}^{2}$*** = 71.2%, ***I${}_{R}^{2}$*** = 76.2%, ***R^**2**^*** = 0.00% Q_**M**_ = 1.5909 (***p*** = 0.45141), Q_**E**_ = 65.148 (***p*** < 0.000)				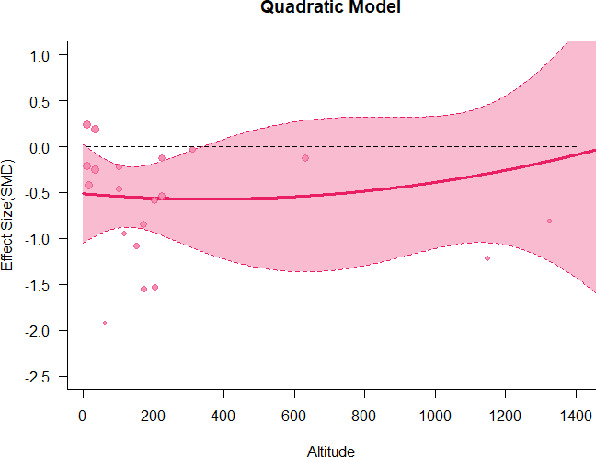 ***I${}_{T}^{2}$*** = 79.0%, ***I${}_{R}^{2}$*** = 83.6%, ***R^**2**^*** = 0.00% Q_**M**_ = 0.444 (***p*** = 0.801), Q_**E**_ = 93.202 (***p*** < 0.000)				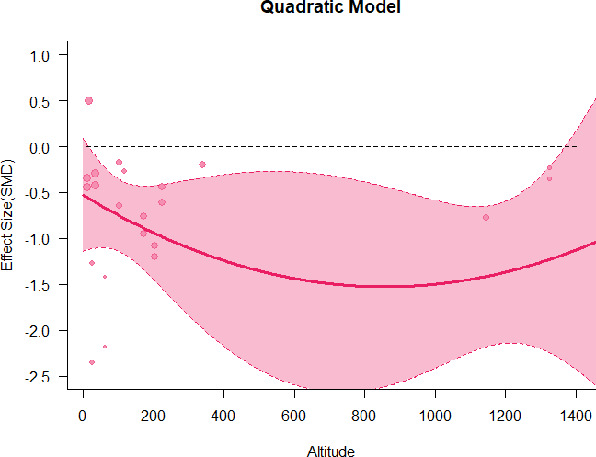 ***I${}_{T}^{2}$*** = 82.0%, ***I${}_{R}^{2}$*** = 87.1%, ***R^**2**^*** = 5.18% Q_**M**_ = 3.398 (***p*** = 0.1829), Q_**E**_ = 110.1955 (***p*** < 0.000)			
		**β**	**SE**	**95% CI**		**β**	**SE**	**95% CI**		**β**	**SE**	**95% CI**		**β**	**SE**	**95% CI**		**β**	**SE**	**95% CI**
	Int	0.5117	0.6656	[−0.7929 1.8163]	Int*	0.9230	0.3647	[0.2082 1.6377]	Int	−0.4320	0.2246	[−0.8721 0.0082]	Int^•^	−0.5116	0.2741	[−1.0489 −0.0257]	Int^•^	−0.4197	0.3085	[−1.0243 0.1848]
	Alt	0.0484	0.0449	[−0.0397 0.1364]	Alt	−0.1044	0.0845	[−0.2701 0.0613]	Alt	0.0022	0.0151	[−0.0274 0.0318]	Alt	−0.0034	0.0184	[−0.0395 0.0326]	Alt	−0.0272	0.0237	[−0.0736 0.0193]
	Alt^2^	−0.0003	0.0004	[−0.0010 0.0004]	Alt^2*^	0.0085	0.0037	[0.0013 0.0157]	Alt^2^	0.0000	0.0001	[−0.0002 0.0002]	Alt^2^	0.0000	0.0001	[−0.0002 0.0003]	Alt^2^	0.0002	0.0002	[−0.0002 0.0005]
Cubic model (unit: 10 m)	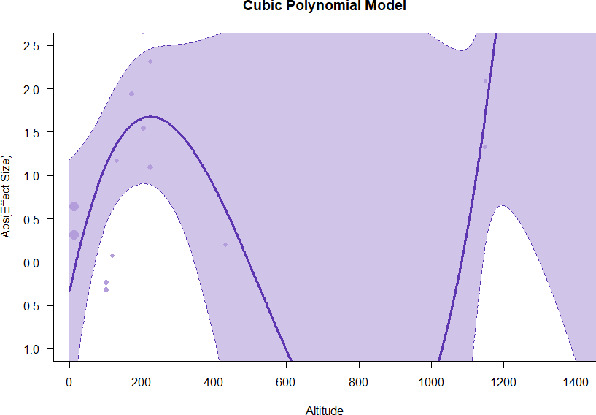 ***I${}_{T}^{2}$*** = 91.5%, ***I${}_{R}^{2}$*** = 92.6%, ***R^**2**^*** = 12.43% Q_**M**_ = 4.8583 (***p*** = 0.183), Q_**E**_ = 112.958 (***p*** < 0.000)				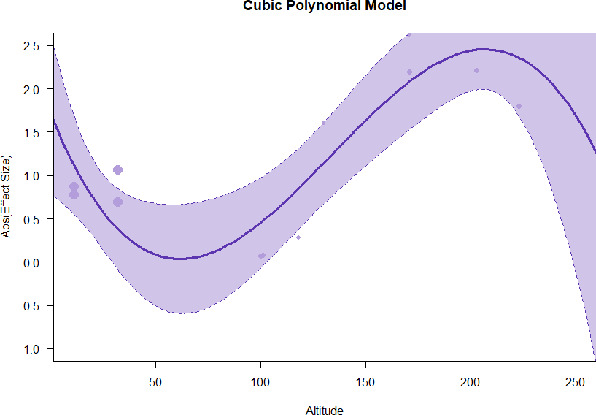 ***I${}_{T}^{2}$*** = 89.7%, ***I${}_{R}^{2}$*** = 72.7%, ***R^**2**^*** = 79.12% Q_**M**_ = 35.300 (***p*** < 0.000), Q_**E**_ = 35.425 (***p*** < 0.000)				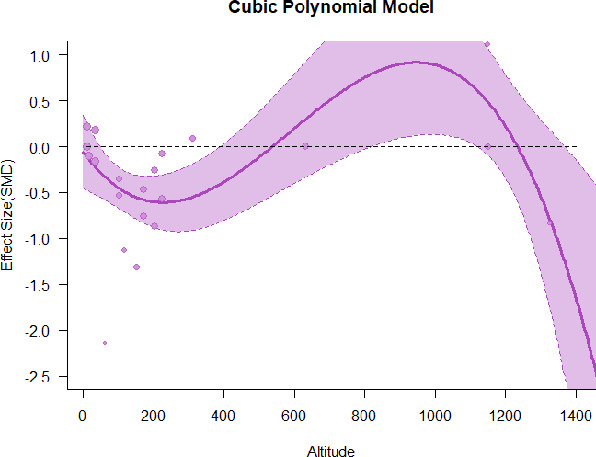 ***I${}_{T}^{2}$*** = 71.2%, ***I${}_{R}^{2}$*** = 61.0%, ***R^**2**^*** = 48.90% Q${}_{\text{M}}^{*}$=12.2503 (***p*** = 0.0066), Q_**E**_ = 43.3160 (***p*** = 0.0004)				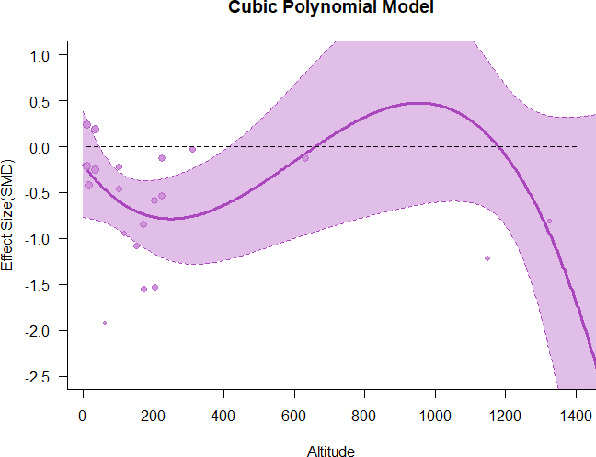 ***I${}_{T}^{2}$*** = 79.0%, ***I${}_{R}^{2}$*** = 80.46%, ***R^**2**^*** = 10.92% Q_**M**_ = 4.578 (***p*** = 0.2055), Q_**E**_ = 74.051 (***p*** < 0.000)				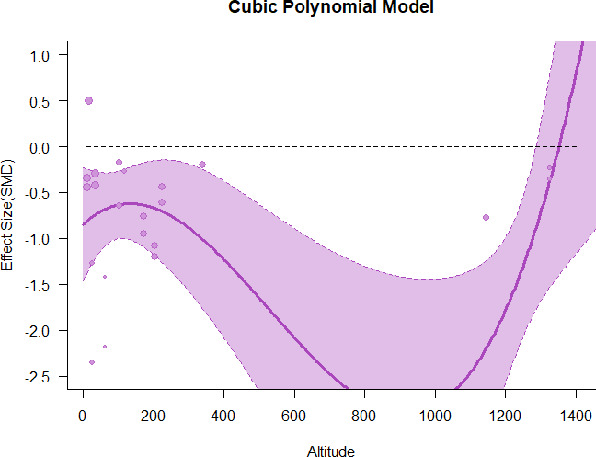 ***I${}_{T}^{2}$*** = 82.0%, ***I${}_{R}^{2}$*** = 84.7%, ***R^**2**^*** = 23.58% Q${}_{\text{M}}^{*}$= 9.7152 (***p*** = 0.0211), Q_**E**_ = 100.6783 (***p*** < 0.000)			
		**β**	**SE**	**95% CI**		**β**	**SE**	**95% CI**		**β**	**SE**	**95% CI**		**β**	**SE**	**95% CI**		**β**	**SE**	**95% CI**
	Int	−0.2981	0.7695	[−1.8063 1.2102]	Int***	1.6733	0.4527	[0.7860 2.5607]	Int	−0.0534	0.20225	[−0.4496 0.3428]	Int	−0.1923	0.2932	[−0.7668 0.3823]	Int*	−0.7381	0.3110	[−1.3477 −0.1286]
	Alt*	0.1938	0.0931	[0.0113 0.3763]	Alt**	−0.6083	0.2336	[−1.0662 −0.1504]	Alt**	−0.0534	0.0205	[−0.0936 −0.0132]	Alt^•^	−0.0526	0.0295	[−0.1104 0.0051]	Alt	0.0300	0.0320	[−0.0327 0.0927]
	Alt^2•^	−0.0055	0.0030	[−0.0113 0.0003]	Alt^2**^	0.0646	0.0249	[0.0158 0.1134]	Alt^2**^	0.0015	0.0005	[0.0006 0.0024]	Alt^2*^	0.0013	0.0007	[0.0001 0.0026]	Alt^2*^	−0.0014	0.0007	[−0.0028 −0.0001]
	Alt^3•^	0.0000	0.0000	[−0.0000 0.0001]	Alt^**3**^	−0.0016	0.0007	[−0.0030 −0.0002]	Alt^3**^	−0.0000	0.0000	[−0.0000 −0.0000]	Alt^3*^	−0.0000	0.0000	[−0.0000 −0.0000]	Alt^3*^	0.0000	0.0000	[0.0000 0.0000]

x-axis, altitude (0–1,400 m); y axis, effect size (SMD); dashed line: baseline with no effect; I${}_{\text{T}}^{2}$, total heterogeneity; I${}_{\text{R}}^{2}$, regression heterogeneity; R^2^, amount of heterogeneity accounted for; β regression weight of altitude per 10 m, Q_M_ test statics of test of moderator; Q_M_ test statics of test of residual heterogeneity; significance codes: 0 “^***^”; 0.001 “^**^”; 0.01 “^*^”; 0.05 “•”; 0.10 “ ”.

We identified the influence of altitude on the effect size of studies captured by the QM index. Regarding emotional restoration, a significant quadratic association between altitude and alleviation of negative emotion was observed. Effect size (SMD) for anxiety (*R*^2^ = 31.41%, Q_M_ = 6.851, *p* = 0.033), depression (*R*^2^ = 39.41%, Q_M_ = 9.732, *p* = 0.008), and confusion (*R*^2^ = 31.10%, Q_M_ = 4.925, *p* = 0.085) had a significant positive quadratic association with altitude. The model estimated the regression weights of quadratic terms to be 0.0004, 0.0004, and 0.0003 for anxiety, depression, and confusion, respectively, which are highly significant (*p* < 0.001). Moreover, estimates of regression weight for linear terms of altitude were significant [*p* < 0.01 (Table 7)]. According to the equation model and its graphical depiction, the amount of alleviation of negative emotions generally increased with altitude, till an altitude of ~600–900 m. After this point, altitude increase predicted a decrease in alleviation efficacy. In the quadratic equation, altitude clarifies ~31–39% of the variance in effect size. The effect size of ROS– an indicator of restorative experience–revealed a significant positive linear association (*R*^2^ = 31.10%, Q_M_ = 4.925, *p* = 0.085), although with a limited altitude ranging from 11 to 223 m. Regarding physiological relaxation, a cubic association was found to be significant in diastolic blood pressure (*R*^2^ = 48.90%, Q_M_ = 12.250, *p* = 0.007) and heart rate (*R*^2^ = 23.58%, Q_M_ = 9.715, *p* = 0.021).

Alongside altitude, other study-level factors were dummy-coded, and an independent meta-ANOVA or meta-regression was performed. Accordingly, seasonal covariates were identified and included as control variables (Table 8). Subsequently, a substantial quadratic association was found between altitude and alleviating effect on anxiety (adjusted *R*^2^ = 96.79%, QM = 70.414, *p* < 0.000), depression (adjusted *R*^2^ = 98.78%, QM = 95.348, *p* < 0.000), fatigue (adjusted *R*^2^ = 64.74%, QM = 29.251, *p* < 0.000). The quadratic terms were found to be significant predictors of effect size on alleviation of anxiety (β = 0.0004, *p* = 0.001), depression (β = 0.0006, *p* < 0.000), fatigue (β = 0.0009, *p* = 0.014). Additionally, the linear quadratic terms were found to be significant predictors of effect size on alleviation of anxiety (β = −0.0589, *p* < 0.000), depression (β = −0.0980, *p* < 0.000), and fatigue (β = −0.1682, *p* = 0.007). The linear association between altitude and ROS was more substantial after inclusion of seasonal covariates (adjusted *R*^2^ = 70.67%, QM = 37.416, *p* < 0.000) and the linear term of altitude with a regression weight 0.0106 (*p* = 0.013). Additionally, diastolic blood pressure demonstrated significant quadratic association with altitude after the inclusion of seasonal covariates (adjusted *R*^2^ = 32.83%, QM = 15.245, *p* = 0.009), and regression weights on quadratic (β = −0.0003, *p* = 0.016) and linear (β = 0.0432, *p* = 0.0012) terms were significant.

**Table 8:** Meta-regression results for binary covariates.

		**Anxiety**		**Depression**		**Confusion**		**Fatigue**		**Hostility**		**Vitality**		**ROS**		**DBP**		**SBP**		**HR**	
		**Coef.**	**Q_M_**	**Coef.**	**Q_M_**	**Coef.**	**Q_M_**	**Coef.**	**Q_M_**	**Coef.**	**Q_M_**	**Coef.**	**Q_M_**	**Coef.**	**Q_M_**	**Coef.**	**Q_M_**	**Coef.**	**Q_M_**	**Coef.**	**Q_M_**
Season	January–March	−1.385***	33.428***(df = 3)	−1.512***	8.070^**•**^(df = 3)	−0.665^**•**^	34.459***(df = 3)	−0.210	8.993^**•**^(df = 3)	−0.253^**•**^	15.419*(df = 3)	1.170^**•**^	21.214***(df = 3)	1.603***	119.336***(df = 3)	−0.281	7.373 (df = 3)	−0.388	4.824 (df = 3)	−0.542	0.339 (df = 3)
	April–June	−0.458*		0.273		−0.892*		−0.961^**•**^		−0.727***		0.715		0.740^**•**^		−0.283		−0.498		−0.388	
	July–September	0.675**		1.870^**•**^		−0.174		−0.825		−0.244		−0.328		−0.755^**•**^		0.344		0.250		−0.219	
	October–December	0.922***		1.249*		0.325		0.310		−0.445^**•**^		−1.333^**•**^		−1.468.		−0.333		−0.147		−0.298	
	Health status	0.217	0.349	−0.700*	3.850^**•**^	0.299	0.746	−0.160	0.138	0.102	0.049	0.567	0.538	N.A.	N.A.	0.214	0.637	0.070	0.048	−0.233	0.508
	Activity intensity	0.610^**•**^	3.065	0.054	0.018	0.312	0.882	0.323	0.537	0.323	1.285	−0.302	0.268	−0.061	0.451	−0.106	0.151	−0.019	0.003	−0.170	0.362
	Recurrent visit	0.208	0.225	−0.442	1.556	0.466	1.025	0.915*	5.274*	0.313	0.658	−1.086	1.064	N.A.	N.A.	−0.481	1.656	−0.357	0.683	0.358	0.747

Coef estimated coefficients; QM test statistic of test of moderators; significance codes: 0 “^***^”; 0.001 “^**^”; 0.01 “^*^”; 0.05 “•”; 0.10 “ ”.

Furthermore, we conducted an additional analysis for studies reporting physical variables possibly related to the altitudinal condition. Table 9 reveals that the thermal index (THI) and illuminance (lx) levels were significantly associated with the effect size of psychological restoration, suggesting that heat and light conditions are potential effect modifiers. Additionally, we found that the intensity of physical activity was significantly associated with the effect size of anxiety relief.

**Table 9:** Meta-regression results for physical variable.

	**Anxiety**		**Depression**		**Confusion**		**Fatigue**		**Hostility**		**Vitality**		**ROS**		**DBP**		**SBP**		**HR**	
	**Coef.**	**Q_M_**	**Coef.**	**Q_M_**	**Coef.**	**Q_M_**	**Coef.**	**Q_M_**	**Coef.**	**Q_M_**	**Coef.**	**Q_M_**	**Coef.**	**Q_M_**	**Coef.**	**Q_M_**	**Coef.**	**Q_M_**	**Coef.**	**Q_M_**
Physical variables																				
THI	−0.0360^**•**^	2.987	−0.0392**	7.7163^**•**^	−0.0498***	11.9127**	−0.0397^**•**^	2.9627	−0.0026	0.0471	0.0847*	6.1846*	0.0674*	6.1517*	0.0123	0.2793	−0.0097	0.1220	−0.0072	0.0582
Illuminance (lx)	−0.0003***	16.839***	−0.0002***	14.6406***	−0.0002***	18.3716***	−0.0003***	20.6610***	−0.0001	1.5975	0.0004**	10.1143**	0.0005***	29.6787***	0.0000	0.0295	−0.0000	0.2537	−0.0000	1.7867

Coef estimated coefficients; QM test statistic of test of moderators; significance codes: 0 “^***^”; 0.001 “^**^”; 0.01 “^*^”; 0.05 “•”; 0.10 “ ”. THI, Temperature-humidity index, an indicator of bioclimatic conditions reflecting heat and cold stress (90). Statistically significant results are in bold.

#### Sensitivity analysis and publication bias assessment

We investigated the influence of individual observations on effect estimation using rstudent, diffits, Cook's D, covratio, τ2, qresid, hat, and dfbetas values. Accordingly, except for hostility and heart rate, for which influential observations were found, most psycho-physiological effect domains were considered to not include influential observations that significantly altered the effect estimate (Supplemental Figures 11–20). Funnel plots indicated the risk of publication bias for the studies investigating depression, hostility, vitality, restorative experience, systolic blood pressure, and heart rate, with significant results of Egger's test (Supplemental Figure 21). Publication bias for studies examining anxiety (*t* = −1.54, *p* = 0.145) and fatigue (*t* = −0.34, *p* = 0.736) were almost negligible. Moderate publication bias was identified for studies on confusion (*t* = 2.05, *p* = 0.063) and diastolic blood pressure (*t* = −2.11, *p* = 0.078). The magnitude of Egger's test was consistent or declined after excluding studies at “high” risk of bias from the analysis of anxiety, fatigue, and diastolic and systolic blood pressure. For depression and heart rate, the publication bias persisted even after the elimination of studies with a “high” risk of bias (Supplemental Figure 22).

### Certainty of evidence

The result of the GRADE assessment and summary of the findings are presented in Table 10. Overall, when all studies were considered, the evidence for an association between altitude and anxiety relief was at moderate-certainty, whereas the evidence for depression, fatigue, and diastolic blood pressure was at low-certainty. After excluding studies at “high” risk of bias, we found evidence of moderate- to high-certainty suggesting altitudinal influence on alleviation of anxiety, fatigue, and diastolic blood pressure. We assessed the certainty of evidence by considering five criteria: risk of bias, inconsistency, indirectness, imprecision, and publication bias. The risk of bias was of concern in analyses of all studies for anxiety, depression, fatigue, and diastolic blood pressure owing to studies rated at high risk of bias. Inconsistency was of concern for anxiety, depression, and fatigue owing to large heterogeneity across studies. However, concerns of inconsistency decreased for fatigue after the elimination of studies with a “high” risk of bias. There were no major issues due to indirectness, except for diastolic blood pressure. Since samples in the high-altitude settings were typically obtained from populations with cardiovascular issues, the physiological impacts may have been overestimated. Therefore, we assessed the serious indirectness for diastolic blood pressure, which may not accurately reflect the physiological outcomes of the general population at high altitude settings. Imprecision was assessed as of concern in analyses of all studies for anxiety, depression, fatigue, and diastolic blood pressure owing to few cases or small sample sizes. In all scenarios, we upgraded the evidence because of the presence of altitude-effect size association. Similarly, we upgraded the evidence level in case of large effects.

**Table 10:** Summary of findings table.

**Certaintyassessment**								**Summary of findings**					
**Outcome (cases)**	**Risk of bias**	**Inconsistency**	**Indirectness**	**Imprecision**	**Publication bias**	**Otherconsideration**	**No. of participants**	**Effect size**	**Altitude range with large effect size (SMD** **≤** −**0.80)**				**Certainty**
								**SMD (95%CI)**	**January–March**	**April– June**	**July– September**	**October–December**	
All studies													
Anxiety (17 cases)	Serious	Serious	No indirectness	Serious	Not serious	Altitude-Effect size association	823	−1.20 (−1.50 to −0.89)	140–1,330 m	0–1,500 m	390–1,090 m	150–1,330 m	Moderate^a^, ^b^, ^d^, ^f^, ^g^ ⊕⊕⊕⊖
Depression (17 cases)	Serious	Serious	No indirectness	Serious	Serious	Altitude-Effect size association	811	−1.01 (−1.34 to −0.67)	210–1,420 m	140–1,490 m	440–1,190 m	170–460 m	Very low^a^, ^b^, ^d^, ^e^, ^f^ ⊕⊖⊖⊖
Fatigue5 (16 cases)	Serious	Serious	No indirectness	Serious	Not serious	Altitude-Effect size association	254	−0.77 (−1.15 to −0.40)	150–1,720 m	170–1,700 m	530–1,340 m	170–1,700 m	Low^a^, ^b^, ^d^, ^f^ ⊕⊕⊖⊖
DBP (21 cases)	Serious	Not serious	Serious indirectness	Serious	Not serious	Altitude-Effect size association	405	−0.32 (−0.55 to −0.10)	Higher than 1,230 m	Higher than 1,030 m	Higher than 1,360 m	Higher than 1,100 m	Low^a^, ^c^, ^d^, ^f^ ⊕⊕⊖⊖
**Excluding studies at high risk of bias**													
Anxiety (10 cases)	Not serious	Serious	No indirectness	Serious	Not serious	Altitude-Effect size association	656	−1.32 (−1.70 to −0.94)	60–1,750 m	560–1,760 m	430–1,380 m	150–1,660 m	High^b^, ^d^, ^f^, ^g^ ⊕⊕⊕⊕
Depression (10 cases)	Not serious	Serious	No indirectness	Serious	Serious	Altitude-Effect size association	586	−0.93 (−1.28 to −0.59)	120–1,730 m	170–1,690 m	490–1,370 m	170–1,690 m	Low ^b^, ^d^, ^e^, ^f^ ⊕⊕⊖⊖
Fatigue (11 cases)	Not serious	Not serious	No indirectness	Serious	Not serious	Altitude-Effect size association	105	−1.16 (−1.49 to −0.83)	130–1,590 m	170–1,540 m	480–1,240 m	130–1,590 m	Moderate ^d^, ^f^, ^g^ ⊕⊕⊕⊖
DBP (18 cases)	Not serious	Not serious	Serious indirectness	Serious	Not serious	Altitude-Effect size association	330	−0.28 (−0.52 to −0.03)	Higher than 1,210 m	Higher than 1,050 m	Higher than 1,340 m	Higher than 1,060 m	Moderate ^c^, ^d^, ^f^ ⊕⊕⊕⊖

Research Question: “In general populations, what is the effect of altitude of forest-based interventions on psycho-physiological effect—emotional restoration, cognitive restoration, stress reduction, physiological relaxation—from interventional studies?” Assessments in all subjects, divided by outcome domains that showed significant associations with altitude in meta-regression (season controlled).

a Serious due to studies rated at high risk of bias;

b Serious due to large heterogeneity across studies (I^2^ > 75%);

c Serious indirectness since samples from high-altitude settings generally had cardiovascular issues;

d Serious due to few cases or small sample sizes;

e Serious due to the risk of publication bias;

f Increased level of certainty due to altitude-effect size association that accounts for heterogeneity across studies;

g Increased level of certainty due to large effect size (upper bound of the 95% CI < −0.80).

## Discussion

Recently, forest-based interventions are recognized as an alternative therapy for disease prevention and public health improvement in several countries. It is critical to identify and describe the potentially effective candidate environments for improving health outcomes for forest-based interventions to be a reliable upstream healthcare approach. Therefore, our review began with the aforementioned PICO question. Accordingly, we aimed to provide an up-to-date summary of evidence that would benefit forest managers, practitioners, and planners who wish to choose suitable forest environments with the appropriate conditions to promote visitor health.

Overall, we found that altitude was significantly associated with alleviation of negative emotions and increase in physiological relaxation. Regarding negative emotions, anxiety, depression, confusion, and fatigue had a significant positive quadratic association with altitude, which implied that the alleviation of negative emotion concurrently increased with altitude up to a certain point, and subsequently, the efficacy declined as the altitude increased thereafter. After summing up a series of meta-regression results, we found that the peak of the regression curve was generally between 600 and 900 m. Conversely, regarding physiological relaxation, diastolic blood pressure demonstrated a significant negative non-linear association with altitude, which suggested that blood pressure-lowering effects tend to become apparent when the altitude of forest settings is high. Moreover, these associations were significant even after controlling for seasonal covariates and became apparent after excluding studies of low methodological quality. Consequently, we calculated the altitude range for large effect sizes based on the evidence in this review with moderate to high certainty. The effect size of anxiety relief and fatigue relief was estimated to be large enough between 560–1,380 and 490–1,240 m, respectively, for all seasons. Additionally, the diastolic blood pressure-lowering effect was predicted to be large enough when the forest-based intervention took place at an altitude of at least 1,050 m.

One intriguing aspect of our findings was that different association patterns emerged between psychological and physiological outcomes. Psychological benefits in environments with natural factors are widely reported from lowland greenspaces to highlands (131–135), and several studies have suggested a altitudinal effect on mood, emotion, cognitive function and behavior (136–140). Spending time at high altitudes reportedly has physiological benefits (141–150) and previous studies adopted outdoor settings >1,500 m to observe apparent changes in pulmonary, cardiac, circulatory, metabolic, and inflammatory outcomes (141, 145, 151, 152). There are multiple potential mechanisms linking altitude with psychophysiological responses, and the observed difference is possibly consequence of the varied altitude-related mechanisms involved in psychological and physiological restoration. Increases in altitude are associated with lower atmospheric pressure, oxygen partial pressure, humidity, and temperature, which could alter metabolic and neuronal activity as a compensatory response of body (153–156). Regarding psychological responses, high altitude exposure is associated with hypobaric hypoxia which alters neurotransmitter function (157, 158), modifies brain bioenergetics (156, 159–161), and changes efficiency of serotonin production (139, 162), all of which have an impact on mood (140, 163–165) and other psychiatric problems (137–139, 166). Regarding the physiological responses, high altitude exposure is also associated with distinctive mountain climate that induces adaptive changes in an individual's metabolic processes (167–173). Short-term intermittent exposure to high altitude has been reported to lower the risk of cardiovascular disease (167, 174), hypertension (168, 174, 175), and metabolic syndrome (155, 175, 176). Relatively extensive studies have been conducted on the molecular mechanisms underlying cellular and organ responses to high-altitude environments. Individual-level psychophysiological reactions, on the other hand, are more complicated and involve interactions between divergent pathways. Therefore, further investigation is needed on the psycho-physiological mechanisms of individuals in natural settings at various altitudes.

Moreover, several recent studies suggest a synergistic interaction between physical activity and the environment at various altitudes (166, 177–179). Physical activity is well-recognized as an effective health-promoting tool, and several studies have shown that physical activity in a natural environment provides a more consistent and powerful effect in improving mood and alleviating psychological stress (22, 177, 180–183). Recently, research on the optimal dose for the intensity and duration of physical activity (184), environmental factors (177, 185), and the optimal combination of physical activity and altitude (166, 178, 179) has been conducted.

Another remarkable finding was the seasonal variation in the altitude range, which is expected to have large effect sizes. Previous studies have outlined the meteorological changes in physiological altitude, and physiological responses at various altitudes are often simulated by adjusting atmospheric pressure and oxygen partial pressure (163, 168, 171–174, 178, 186). According to Millet and Devec (187), physiological altitude varies by up to 250 m per day and up to 500 m per year due to potential changes in barometric pressure even at the same point. In the future, formulating more comprehensive guidelines that account for seasonal variations by repeatedly evaluating the efficacy of forest-based interventions for different seasons may be possible. Recently, a investigations along similar lines have already been initiated (105, 188–190).

Notably, the minimum altitude for large effects tended to be higher from July to September compared with the other months. This may be because the areas where the studies were conducted typically experience summer between July and September. Based on the Köppen-Geiger climate classification for each study site (191), all regions have a distinct four-season pattern with the highest temperatures between June and September. In this context, meteorological factors (186, 192) possibly have contributed to the higher elevation range from July to September. It can also be influenced by other potential variables in the ambient environment.

Several published studies have demonstrated that physiological, biochemical, and perceptual changes with altitude were mainly due to the ambient environment (122, 144, 148, 193–195). There have been suggestions on altitude-related elements and their physiological effects; namely, atmospheric pressure (143, 144, 147, 149, 152, 195, 196), air oxygen concentration (143, 146, 149, 194), negative ions (122, 143, 144, 147, 149, 152, 193, 194, 196–198), absence of pollutants or allergens (144, 149, 152), solar radiation and UVB intensity (143, 144, 149, 199), temperature (143, 144, 149, 196), and relative humidity (143, 149, 196). Several factors, which may vary with altitude, have been reported as mediators of psychological restoration; namely, visually perceivable natural components (2, 43, 54, 55, 58, 59, 68, 71, 133), forest structure and understory vegetation (2, 45, 55, 68–71, 75), microclimate and thermal comforts (55, 79, 83, 131, 132, 135, 200), in-forest light conditions (54, 201, 202), airborne substances (28, 203–205), and pollutant concentrations (131, 132, 135). Thus, future studies should perform multi-faceted environmental measurements in conjunction with forest-based interventions to elucidate the underlying mechanisms or causal variables of the altitudinal effects. This will provide robust and credible evidence of the ideal delivery mechanisms to decision-makers in charge of forest-based initiatives.

To the best of our knowledge, the effects of altitude and physical factors remain largely unexplored in the literature on nature-based interventions. In this review, we have primarily focused on altitude because it is a simple and accessible indicator of the in-forest ambient environment. We have assumed altitude to be an effect modifier of the psychophysiological effects of forest-based intervention and summarized data on the magnitude and shape of the association. Our findings are significant because they clarify the link between altitude and health benefits of forest exposure. The identified associations may be considered to maximize the health advantages of forest-based interventions.

Our study has some limitations. First, our findings are observational. We have provided a quantitative summary of all available evidence *via* meta-analysis; however, we have not provided additional data to test the capability of the regression models in predicting unseen data. Nevertheless, our findings can identify potentially significant predictors and be used to generate hypotheses for future verification studies. Second, there is a risk of overestimating physiological effects in high-altitude conditions. Regarding interventions which investigated physiological effects, most interventions in low-altitude settings have carried out in urban park or urban forest. Moreover, most interventions in high-altitude settings have involved participants with cardiovascular disease or symptoms. Previous studies have noted that these participants frequently exhibited a greater physiological reaction to a given intervention (206, 207). Further investigations of other representative populations in high-altitude environments to derive accurate estimates are needed. Third, stress-related results and other biomarkers could not be analyzed owing to the limited number of studies. Therefore, further studies examining the stress-reducing effects of forest-based interventions, along with descriptions of the forest environment are necessary. Fourth, most of the included studies were rated as “moderate” to “high” risk of bias owing to the absence of concealment of random allocation and lack of participant blinding. Forest-based interventions are may inevitably have a higher risk of bias regarding random assignment and outcome measurement. These essentially require participants to visit a forest environment or participate in a directed program; thus, assignment concealment and participant blinding are rendered more difficult and increase the risk of bias during randomization. Regarding self-reported measurements, lack of participant blinding may be a major contributor to a higher risk of bias in outcome measures. To manage this, Bratman et al. (106) assigned an unrelated task (i.e., taking pictures) to disguise the purpose of the intervention, and Bielinis et al. (105) explained the study intent after the experiment. Additionally, more than half of the included studies did not provide a detailed randomization process and pre-registered analysis plan. Thus, future investigations should utilize trial registry platforms and sophisticated randomization methods to improve the quality of evidence (15, 208).

## Conclusion

This review and meta-analysis explored the effect of altitude on the health benefits of forest exposures. Overall, we found significant non-linear associations between altitude and the magnitude of health effects. Based on the meta-regression results, we have approximated altitude ranges for psychological and physiological restoration with large effect sizes. We observed the different association patterns between altitude and psychological and physiological effects. We also identified the seasonal variation in altitude range for large effect sizes. We discussed the potential mechanisms involves in altitudinal effects shown in our findings. Recent research in nature-based interventions and preventive medicine has taken a more systematic approach, including examining environmental and activity conditions to estimate the optimal dose of nature to maximize therapeutic effectiveness. Despite some limitations, these findings supplement the available evidence on selecting nature environment for health improvement initiatives. Further investigative studies examining the multi-faceted aspects of environmental factors are needed to advance and implement forest-based interventions beyond research contexts.

## Data availability statement

The original contributions presented in the study are included in the article/Supplementary material, further inquiries can be directed to the corresponding author.

## Author contributions

EK: conceptualization, formal analysis, investigation, methodology, project administration, supervision, visualization, writing—original draft, and writing—review and editing. SP: formal analysis, methodology, visualization, writing—original draft, and writing—review and editing. SK: investigation, writing—original draft, and writing—review and editing. YC: investigation, writing—original draft, writing—review and editing, and methodology. JC: conceptualization, project administration, supervision, writing—original draft, writing—review and editing, and methodology. GK: formal analysis, investigation, methodology, writing—original draft, and writing—review and editing. All authors contributed to the article and approved the submitted version.

## Conflict of interest

The authors declare that the research was conducted in the absence of any commercial or financial relationships that could be construed as a potential conflict of interest.

## Publisher's note

All claims expressed in this article are solely those of the authors and do not necessarily represent those of their affiliated organizations, or those of the publisher, the editors and the reviewers. Any product that may be evaluated in this article, or claim that may be made by its manufacturer, is not guaranteed or endorsed by the publisher.
