# Supplementary material for: Is altitude a determinant of the health benefits of nature exposure? A systematic review and meta-analysis
Source: Front Public Health. 2022 Nov 25;10:1021618. doi: 10.3389/fpubh.2022.1021618 (PMC9732270; doi:10.3389/fpubh.2022.1021618)
Supplement: Supplementary file 1 [file Table_1.DOCX]

Supplementary Material

**SUPPLEMENTAL TABLE S1.** PRISMA Report (modified) for Systematic Reviews Submitted to Environment International.

Title of submitted paper and corresponding author: Is altitude a determinant of the health benefits of forest exposure? A systematic review and meta-analysis. Geonwoo Kim

| Section and Topic | Item # | Checklist item | Location where item is reported |
| --- | --- | --- | --- |
| TITLE | | |  |
| Title | 1 | Identify the report as a systematic review. | Page 1 |
| ABSTRACT | | |  |
| Abstract | 2 | See the PRISMA 2020 for Abstracts checklist. | Page 1 |
| INTRODUCTION | | |  |
| Rationale | 3 | Describe the rationale for the review in the context of existing knowledge. | Page 2-3 |
| Objectives | 4 | Provide an explicit statement of the objective(s) or question(s) the review addresses. | Page 3 |
| METHODS | | |  |
| Eligibility criteria | 5 | Specify the inclusion and exclusion criteria for the review and how studies were grouped for the syntheses. | Page 4-5  Table 1 |
| Information sources | 6 | Specify all databases, registers, websites, organisations, reference lists and other sources searched or consulted to identify studies. Specify the date when each source was last searched or consulted. | Page 4 |
| Search strategy | 7 | Present the full search strategies for all databases, registers and websites, including any filters and limits used. | Table S2 |
| Selection process | 8 | Specify the methods used to decide whether a study met the inclusion criteria of the review, including how many reviewers screened each record and each report retrieved, whether they worked independently, and if applicable, details of automation tools used in the process. | Page 4 |
| Data collection process | 9 | Specify the methods used to collect data from reports, including how many reviewers collected data from each report, whether they worked independently, any processes for obtaining or confirming data from study investigators, and if applicable, details of automation tools used in the process. | Page 4 |
| Data items | 10a | List and define all outcomes for which data were sought. Specify whether all results that were compatible with each outcome domain in each study were sought (e.g. for all measures, time points, analyses), and if not, the methods used to decide which results to collect. | Page 4 |
|  | 10b | List and define all other variables for which data were sought (e.g. participant and intervention characteristics, funding sources). Describe any assumptions made about any missing or unclear information. | Page 4 |
| Study risk of bias assessment | 11 | Specify the methods used to assess risk of bias in the included studies, including details of the tool(s) used, how many reviewers assessed each study and whether they worked independently, and if applicable, details of automation tools used in the process. | Page 5 |
| Effect measures | 12 | Specify for each outcome the effect measure(s) (e.g. risk ratio, mean difference) used in the synthesis or presentation of results. | Page 5 |
| Synthesis methods | 13a | Describe the processes used to decide which studies were eligible for each synthesis (e.g. tabulating the study intervention characteristics and comparing against the planned groups for each synthesis (item #5)). | Page 5-6 |
|  | 13b | Describe any methods required to prepare the data for presentation or synthesis, such as handling of missing summary statistics, or data conversions. | Page 5-6 |
|  | 13c | Describe any methods used to tabulate or visually display results of individual studies and syntheses. | Page 5-6 |
|  | 13d | Describe any methods used to synthesize results and provide a rationale for the choice(s). If meta-analysis was performed, describe the model(s), method(s) to identify the presence and extent of statistical heterogeneity, and software package(s) used. | Page 5-6 |
|  | 13e | Describe any methods used to explore possible causes of heterogeneity among study results (e.g. subgroup analysis, meta-regression). | Page 5-6 |
|  | 13f | Describe any sensitivity analyses conducted to assess robustness of the synthesized results. | Page 6 |
| Reporting bias assessment | 14 | Describe any methods used to assess risk of bias due to missing results in a synthesis (arising from reporting biases). | Page 6 |
| Certainty assessment | 15 | Describe any methods used to assess certainty (or confidence) in the body of evidence for an outcome. | Page 6 |
| RESULTS | | |  |
| Study selection | 16a | Describe the results of the search and selection process, from the number of records identified in the search to the number of studies included in the review, ideally using a flow diagram. | Page 7-8  Figure 1 |
|  | 16b | Cite studies that might appear to meet the inclusion criteria, but which were excluded, and explain why they were excluded. | Page 7-8 |
| Study characteristics | 17 | Cite each included study and present its characteristics. | Page 7-8  Table 2 |
| Risk of bias in studies | 18 | Present assessments of risk of bias for each included study. | Page 8  Table 3 and 4 |
| Results of individual studies | 19 | For all outcomes, present, for each study: (a) summary statistics for each group (where appropriate) and (b) an effect estimate and its precision (e.g. confidence/credible interval), ideally using structured tables or plots. | Page 9  Table 5  Figure S1-S10 |
| Results of syntheses | 20a | For each synthesis, briefly summarize the characteristics and risk of bias among contributing studies. | Page 10  Figure S11-20 |
|  | 20b | Present results of all statistical syntheses conducted. If meta-analysis was done, present for each the summary estimate and its precision (e.g. confidence/credible interval) and measures of statistical heterogeneity. If comparing groups, describe the direction of the effect. | Page 9-10  Table 5-7  Figure S1-S10 |
|  | 20c | Present results of all investigations of possible causes of heterogeneity among study results. | Page 9-10  Table 8-9 |
|  | 20d | Present results of all sensitivity analyses conducted to assess the robustness of the synthesized results. | Page 10  Figure S11-20 |
| Reporting biases | 21 | Present assessments of risk of bias due to missing results (arising from reporting biases) for each synthesis assessed. | Page 10  Figure S21-S22 |
| Certainty of evidence | 22 | Present assessments of certainty (or confidence) in the body of evidence for each outcome assessed. | Page 11  Table 10 |
| DISCUSSION | | |  |
| Discussion | 23a | Provide a general interpretation of the results in the context of other evidence. | Page 12-13 |
|  | 23b | Discuss any limitations of the evidence included in the review. | Page 13 |
|  | 23c | Discuss any limitations of the review processes used. | Page 13 |
|  | 23d | Discuss implications of the results for practice, policy, and future research. | Page 13-14 |
| OTHER INFORMATION | | |  |
| Registration and protocol | 24a | Provide registration information for the review, including register name and registration number, or state that the review was not registered. | Page 4 |
|  | 24b | Indicate where the review protocol can be accessed, or state that a protocol was not prepared. | Page 4 |
|  | 24c | Describe and explain any amendments to information provided at registration or in the protocol. | No amendments in the protocol |
| Support | 25 | Describe sources of financial or non-financial support for the review, and the role of the funders or sponsors in the review. | Page 14  No funding |
| Competing interests | 26 | Declare any competing interests of review authors. | Page 14  No conflicting interests |
| Availability of data, code and other materials | 27 | Report which of the following are publicly available and where they can be found: template data collection forms; data extracted from included studies; data used for all analyses; analytic code; any other materials used in the review. | Authors do not have permission to share data |

*From:*  Page MJ, McKenzie JE, Bossuyt PM, Boutron I, Hoffmann TC, Mulrow CD, et al. The PRISMA 2020 statement: an updated guideline for reporting systematic reviews. BMJ 2021;372:n71. doi: 10.1136/bmj.n71

**SUPPLEMENTAL TABLE S2.** Details of literature search on online databases.

| Databases | Strategy |
| --- | --- |
| PubMed  Embase  PsycINFO  Web of Science  Scopus  Filters:  English, Human  Date (publication):  ~ Feb 2020 | #1 Environmental settings  "Nature" OR "natural environment" OR "natural setting" OR "nature-based" OR "forest" OR "forest garden" OR "forest park" OR "urban forest" OR "forested area" OR "woodland" OR "green" OR ”landscape” OR "park" OR "urban wild" OR "trees" OR "nature reserve" OR "national park" OR "public park" OR "urban park" OR "open space" OR "municipal park"  #2 Interventions  "experience" OR "recreation" OR “exposure” OR “contact” OR “connect” OR "viewing" OR “looking” OR “watching” OR “appreciation” OR "walking" OR “walk” OR "staying" OR “stay” OR “sitting” OR "exercise" OR “athletic” OR ”tracking” OR "hiking" OR "meditation" OR “meditative” OR "mindfulness" OR "immersive" OR "rehabilitation" OR "activity" OR "program" OR "session" OR "tour" OR "trip" OR "travel" OR “visit” OR "therapy" OR “intervention” OR “therapeutic” OR "healing" OR "shinrin-yoku" OR "bathing" OR "basking" OR “spending” OR "time" OR "minute" OR "hour" OR "Nordic walk" OR "Terrainkur" OR "Kurort" OR "friluftslive" OR "skogsbad" OR "MEIJÄN POLKU " OR "healthy park healthy people"  #3 Region, location, and altitude  “Europe” OR “Nordic” OR “Sweden” OR “Swedish” OR “Norway” OR “Norwegian” OR “Denmark” OR “Danish” OR “Finland” OR “Finnish” OR “Iceland” OR “Icelandic” OR “Alpine” OR “Poland” OR “Polish” OR “Germany” OR “German” OR “Deutschland” OR “Switzerland” OR “Swiss” OR “Austria” OR “Austrian” OR “France” OR “French” OR “Slovenian” OR “Liechtenstein” OR “Italy” OR “Italian” OR “Greece” OR “Greek” OR “Spain” OR “Spanish” OR “Portugal” OR “Portuguese” OR “Slovenian” OR “Bulgaria” OR “Bulgarian” OR “Korea” OR “Korean” OR “Japan” OR “Japanese” OR “China” OR “Chinese” OR “Taiwan” OR “Taiwanese” OR “Philippines” OR “Filipino” OR “Singapore” OR “Singaporean” OR “Vietnam” OR “Vietnamese” OR “Myanmar” OR “Burmewe” OR “Malaysia” OR “Malaysian” OR “USA” OR “America” OR “American” OR “Canada” OR “Canadian” OR “Australia” OR “Australian” OR “New Zealand” OR “altitude” OR “elevation” OR “location” OR “latitude” OR “longitude” OR “environmental factor” OR “microclimate” OR “physical environment”  #4 health outcomes  “health” OR “well-being” OR "mental" OR "physical" OR "therapeutic" OR “psychological” OR “psychiatric” OR “physiological” OR “recovery” OR “restorative” OR “relief” OR “restoration” OR “mood” OR “affective state” OR “POMS” OR “PANAS” OR “anxiety” OR “STAI” OR “self-reporting anxiety scale” OR “depression” OR “BDI” OR “self-reporting” OR “vitality” OR “SVS” OR “cognitive” OR “mental fatigue” OR “PRS” OR “ROS” OR “chronic” OR “acute” OR "stress reduction” OR “stress recovery” OR “stress coping” OR “stress-related” OR “distress” OR “stress marker” OR “biomarker” OR “saliva” OR “cortisol” OR “amylase” OR “adrenaline” OR” noradrenaline” OR “epinephrine” OR “norepinephrine” OR “serotonin” OR “melatonin” OR “relaxation” OR “lowering effect” OR “blood pressure” OR “SBP” OR “systolic blood pressure” OR “DBP” OR “diastolic blood pressure” OR “pulse rate” OR “heart rate” OR “HRV” OR “heart rate variability” OR “SDNN” OR “RMSSD” OR “LF” OR “HF” OR “LF/HF” OR “pNN50” OR “skin conductance” OR “SCR” OR “brain wave” OR “prefrontal activity” OR “SpO2” OR “EEG”  #5 study design  "intervention stud" OR "RCT" OR "randomized controlled" OR "randomly" OR "randomised" OR "trial" OR "quasi-experiment" OR "filed experiment" OR “field study” OR "pilot study" OR "pretest" OR "posttest" OR "pre intervention" OR "post intervention" OR "cross over study" OR "comparative study"  Search keywords  #1 AND #2 AND #3 AND #4 AND #5 |

**SUPPLEMENTAL TABLE S3.** Environmental characteristics of the included studies ordered by first author’s name and publication year.

| Reference | Altitude | Location | Time of  measure | Environmental variables |
| --- | --- | --- | --- | --- |
| Bielinis et al., 2019 | 130m | forest trail in Olsztyn named Las Gronicki , Poland (53°45’24”N 20°26’23”E) | March 2018  (10:10~10:25) | Temp: -0.5°C  RH: 100%  wind speed: 2.5 m/s  atm: 995hPa |
| Bratman et al., 2015 | 50m ~ 130m | Park near Stanford University known as “The Dish”, USA | All seasons | N.A. |
| Chun et al., 2017 | 450m~ 600m | Recreational forests in Gyenggi-do, Republic of Korea  (37°46’19”N 127°20’20”E) | N. A. | N.A. |
| de Brito et al., 2020 | 295m~ 315m | Wood Duck Trail of the Minnesota Landscape Arboretum (MLA), USA (44°51’40”N 93°37’15”W) | N. A  . | N. A. |
| Djernis et al., 2021 | 44m | Therapy garden belonging to the University of Copenhagen, Denmark  (55°52’01”N 12°30’28”E) | April and August 2016;  April and August 2017 | N. A. |
| Dolling et al., 2017 | 89m~ 92m | Forest located in the boreal zone near lake Bäcksjön, Sweden  (63°58′ N, 20°21′ E) | Sep-Dec  March-June | N. A. |
| Grazuleviciene  et al., 2015 | 55m~ 69m | Pine tree park located within a 5 min walk of the Cardiology Clinic, accessed through clinic park (54°55’04”N 23°54’54”E) | May–September 2013  (12:00-15:00) | N. A. |
| Han et al., 2016 | 280m~ 400m | Saneum Natural Recreation Forest in Yangpyeong county of Gyeonggi Province, Republic of Korea | June 2014 September 2014; September 2015 (11:00-13:00; 14:00-17:30; 19:00~21:00) | Temp: 20.35°C  RH: 61.10%  wind speed: 1.01m/s |
| Han, 2017 | 110m | Forest park in National Chin-Yi University of Technology, Taiwan (24°08’56”N 120°43’47”E) | Oct 2013 – Feb 2014 (8:00~17:00) | N.A. |
| Harte and Eifert, 1995 | 30m | Outdoor route around James Cook University campus with trees, Australia (19°19’32”S 146°45’22”E) | N.A. | N.A. |
| Janeczko et al., 2020 | 101m | Green suburbs with trees (52°09’44”N 21°02’59”E) | November 2019 | Temp: 8°C  RH: 79%  wind speed: 4.16m/s  atm: 996hPa  illuminance: 1969.33lx |
|  | 100m | Coniferous forest named Kabaty Forest, Poland (52°07’02”N 21°05’10”E) |  | illuminance: 448lx |
|  | 118m | Deciduous forest named Sobieski Forest, Poland (52°14’27”N 21°10’43”E) |  | illuminance: 360lx |
| Lanki et al., 2017 | 11m | Urban park named Alppipuisto, Finland (60°11’25”N 24°56’15”E) | Aug-Sep 2011;  Aug-Sep 2012 | Temp: 22.3°C(sedentary) 19.9°C(walking) |
|  |  | Urban forest named Keskuspuisto, Finland (60°13’27”N 24°55’06”E) |  | Temp: 24.7°C(sedentary) 23.5°C(walking) |
| Lee and Lee, 2014 | 150m | Chamaecyparis obtuse forest in Janghung, Republic of Korea | November 2012 | Temp: 16.7°C  RH: 52.3%  wind speed: m/s  illuminance: lx |
| Li et al., 2016 | 1130m~ 1170m | forest park named Akasawa Shizen Kyuyourin in Agematsu, Nagano Prefecture, Japan (35°43’39”N 137°37’23”E) | August 2015 (11:00-12:20) (13:40-15:00) | Temp: 19.1°C  RH: 94.3% |
| Liu et al., 2021 | 171m | Mixed forest, Changping, China (40°15’09”N 116°16’37”E) | June 2021 | Temp: 27.44°C  RH: 44.33%  wind speed: 0.23m/s  illuminance: 4944.44lx |
|  | 203m | Deciduous forest, Changping, China (40°15’23”N 116°16’39”E) |  | Temp: 27.78°C  RH: 43.44%  wind speed: 0.23m/s  illuminance: 3424.44lx |
|  | 223m | Coniferous forest, Changping, China (40°15’35”N 116°16’43”E) |  | Temp: 26.33°C  RH: 39.89%  wind speed: 0.64m/s  illuminance: 5994.44lx |
| Mao et al., 2012a | 1324m | broad-leaved evergreen forest named Zhejiang Baimashan Forest Park in Suichang County, China (28°37’09”N 119°08’52”E) | July 2011 (8:00-9:30, 16:00~17:30) | Temp: 25.5°C  RH: 81.8% |
| Mao Gen et al., 2012b | 392m | Wuchao Mountain Forest in Hangzhou, China (30°11’16”N 120°00’45”E) | September (9:00-11:00 14:30-16:30) | N.A. |
| Mao et al., 2016 | 522m | Forest Park named Huangtan located in Pan’an County, Zhejiang Province, China (28°59’45”N 120°26’44”E) | August 2015 (8:00-9:30 16:00-17:30) | Temp: 25.2°C  RH: 77.3% |
| Meyer et al., 2016 | 431m | forest trail located in Göttingen, Germany (51°32’05”N 10°03’09”E) | July 2015 | Temp: 22.5°C |
| Morita et al., 2007 | 90m~ 220m | University of Tokyo Chiba Forest, Japan | N.A. | N.A. |
| Song et al., 2014 | 20~ 30m | Urban park named Kashiwa-no-ha Park, Chiba Prefecture, Japan (start point: 35°53’34”N 139°56’34”E  end point: 35°53’44”N 139°56’25”E) | May 2013 (10:00-12:00 13:00-15:00) | Temp: 24.7°C  RH: 39.2% |
| Song et al., 2015a | 20~ 30m | Urban park named Kashiwa-no-ha Park, Chiba Prefecture, Japan (start point: 35°53’34”N 139°56’34”E  end point: 35°53’44”N 139°56’25”E) | October 2014 (9:00-11:00, 13:00-15:00) | Temp: 18°C  RH: 71.5% |
| Song et al., 2015b | 1120m~ 1168m | Akasawa natural recreation forest located in Agematsu town of Nagano Prefecture, Japan  (35°43’39”N 137°37’23”E) | N.A. | Temp: 21.4°C  RH: 82.3% |
| Sung et al., 2012 | 280m~ 400m; 850m~ 1000m | Two recreation forest sites, Hoengseong and Saneum, in Kangwon-do, Republic of Korea | April 2009 | N.A. |
| Tyrväinen et al., 2014 | 11m | Urban park named Alppipuisto, Finland (60°11’25”N 24°56’15”E) | Aug-Sep 2011;  Aug-Sep 2012;  May-June, 2012 | Temp: 22.4°C |
|  | 14m~ 49m | Urban forest named Keskuspuisto, Finland (60°13’32”N 24°55’00”E) |  |  |
| Wang et al., 2021 | 15m | Crescent Lake Park, Qinhuai District, Nanjing, China (32° 02′ 01.3′′N, 118° 49′ 40.5′′E) | Aug-Dec 2019 | N.A. |
| Zeng et al., 2020 | 634m | Bamboo forest located near the city of Ya’an (28°28’22”N, 105°0’19”E) | September 2017 (9:30-09:45 15:30-15:45) | Temp: 26.9°C  RH: 79%  wind speed: 0.1m/s  illuminance: 877.1lx |
|  | 754m | Bamboo forest located near the city of Dujiangyan (31°44’54”N, 103°25’42”E) |  | Temp: 18.7°C  RH: 87.5%  wind speed: 0.4m/s  illuminance: 815.2lx |
|  | 892m | Bamboo forest located near the city of Yibin (28°28’22”N, 105°0’19”E) |  | Temp: 23.2°C  RH: 74.5%  wind speed: 0.2m/s  illuminance: 3774.7lx |

atm atmospheric pressure: RH relative humidity; temp temperature.

**SUPPLEMENTAL FIGURE S1.** Forest plot for alleviation of anxiety (arranged in ascending order by altitude).


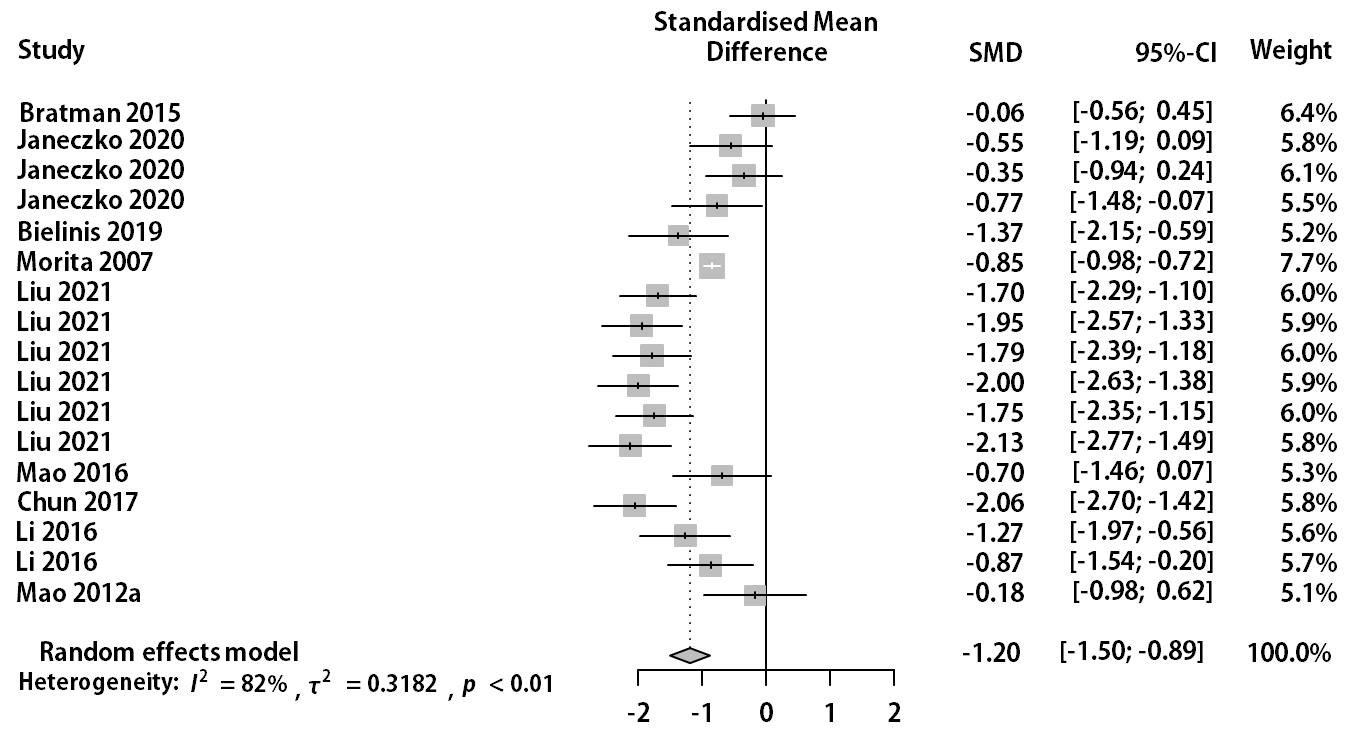


**SUPPLEMENTAL FIGURE S2.** Forest plot for alleviation of depression (arranged in ascending order by altitude).


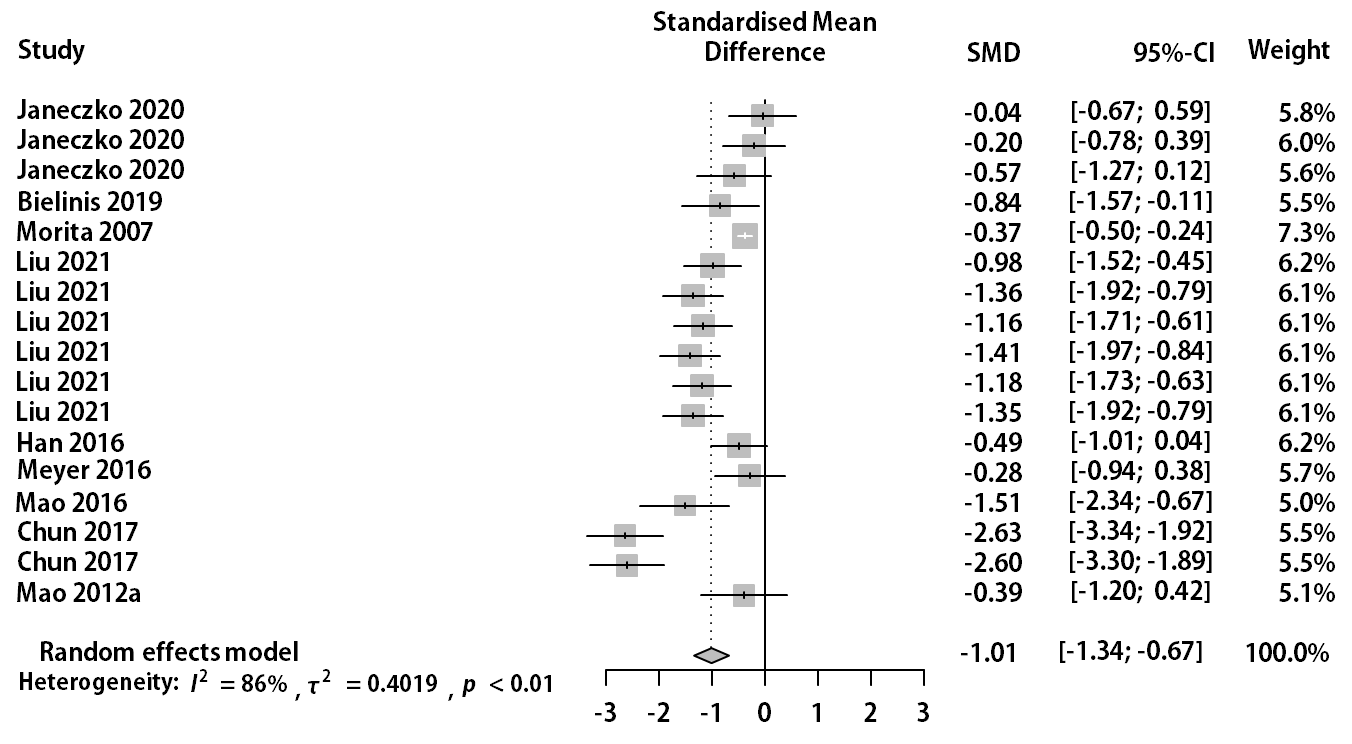


**SUPPLEMENTAL FIGURE S3.** Forest plot for alleviation of confusion (arranged in ascending order by altitude).


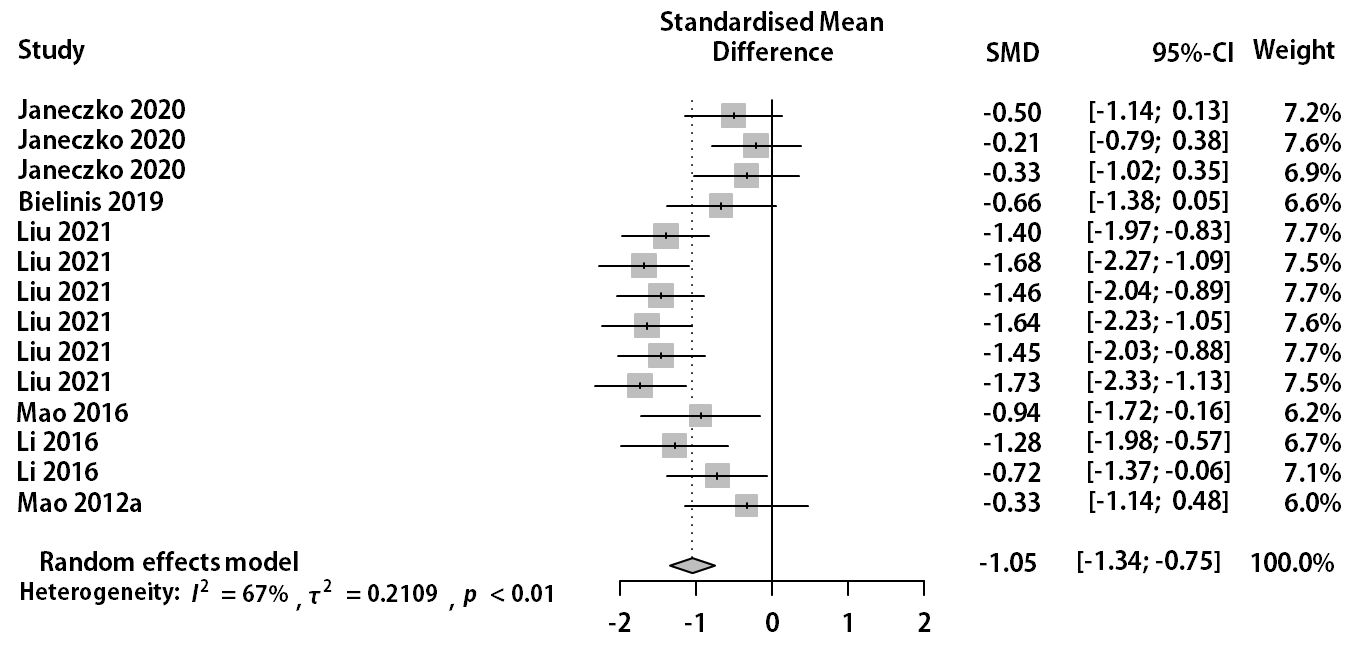


**SUPPLEMENTAL FIGURE S4.** Forest plot for alleviation of fatigue (arranged in ascending order by altitude).


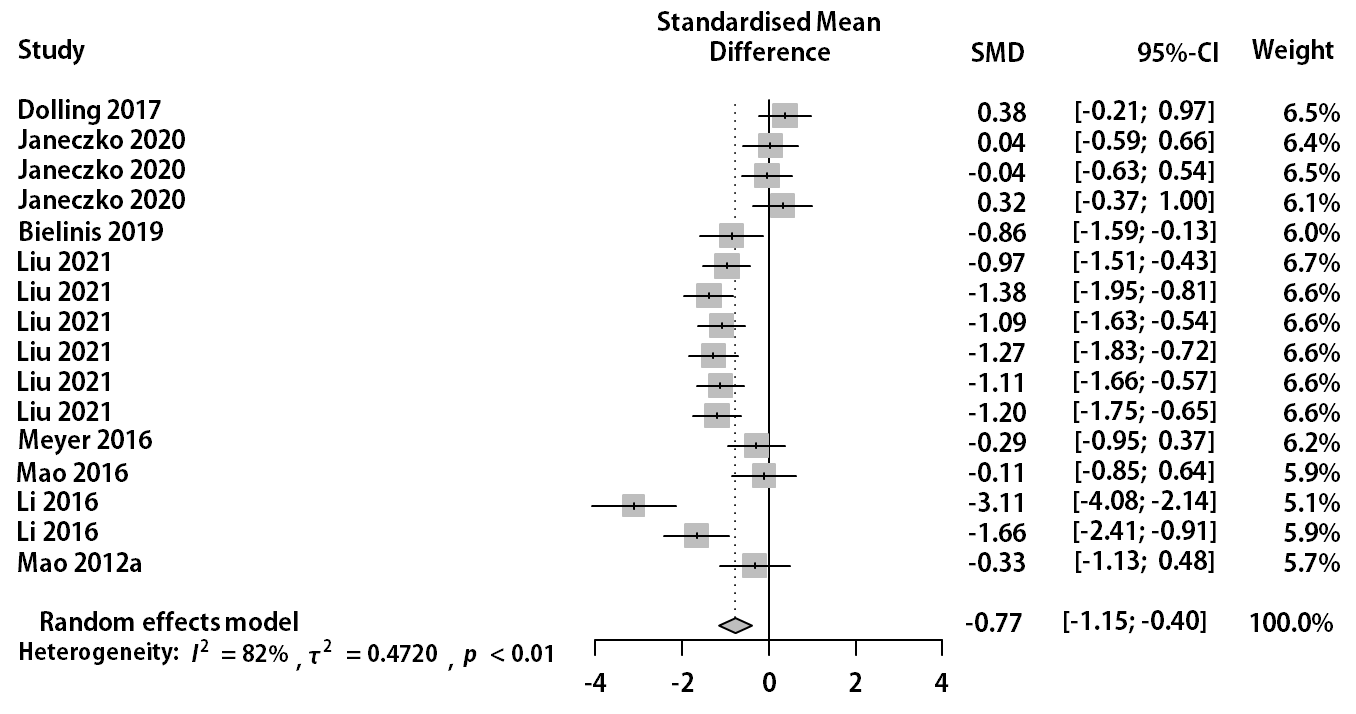


**SUPPLEMENTAL FIGURE S5.** Forest plot for alleviation of hostility (arranged in ascending order by altitude).


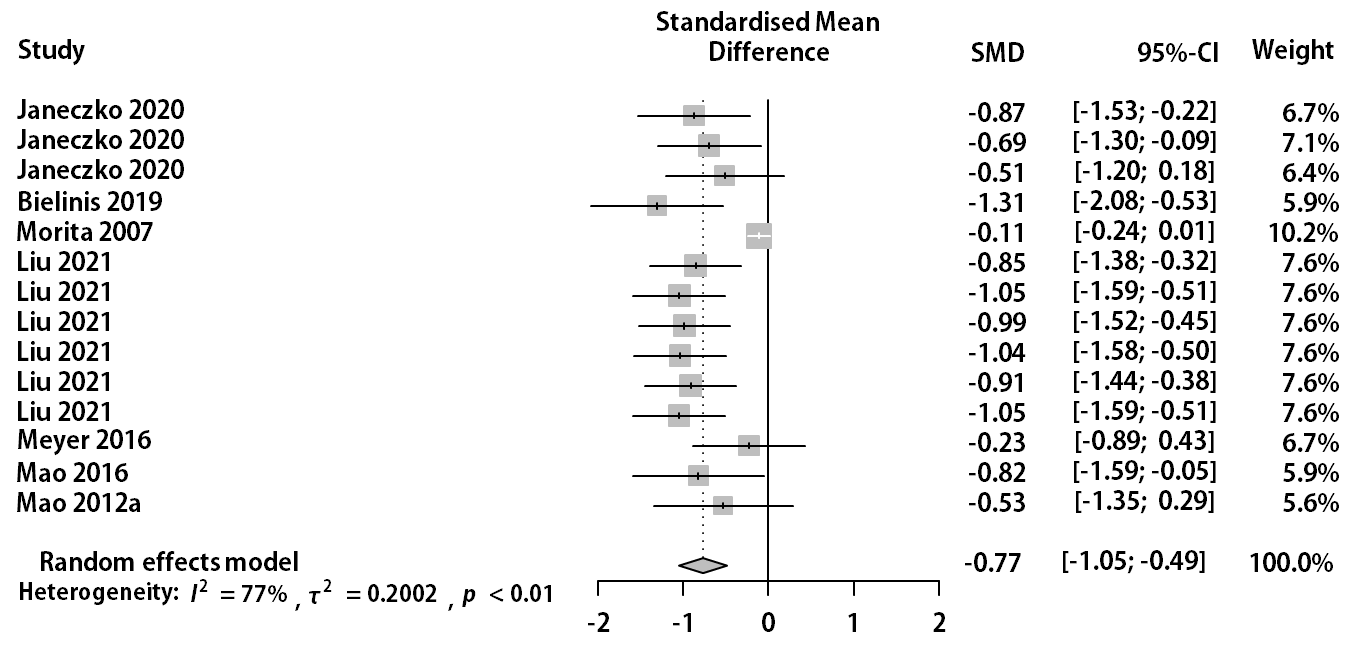


**SUPPLEMENTAL FIGURE S6.** Forest plot for improvement in vitality (arranged in ascending order by altitude).


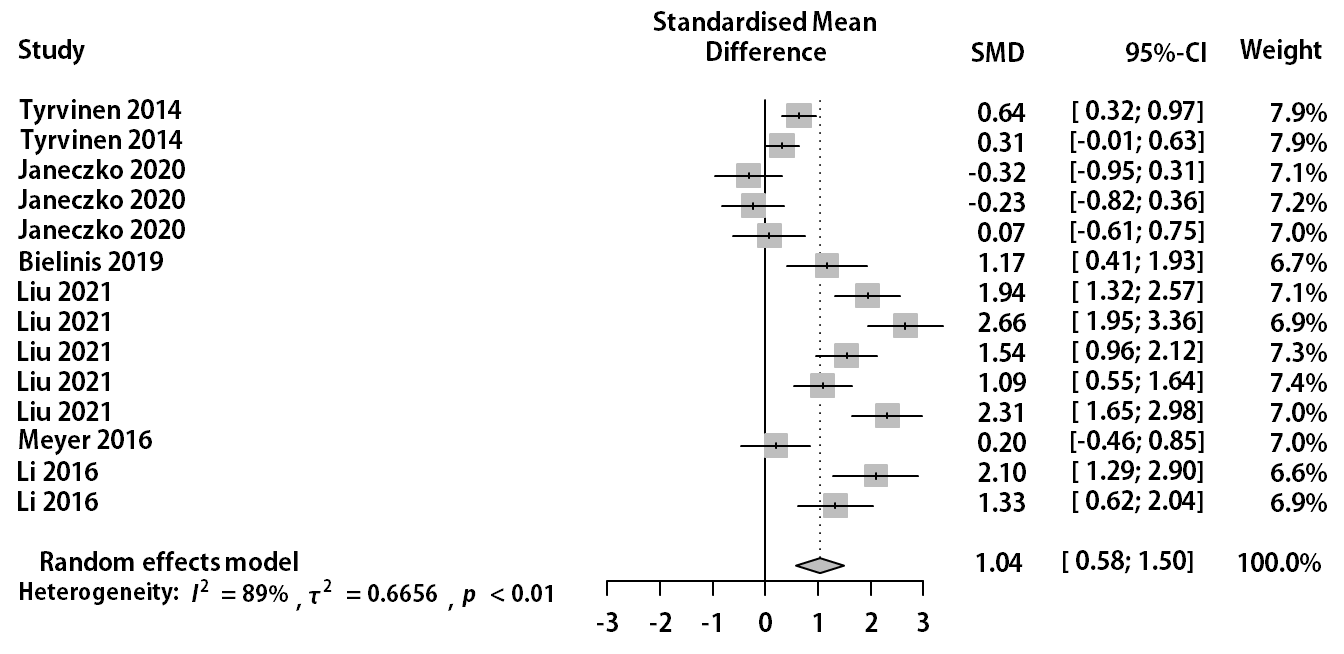


**SUPPLEMENTAL FIGURE S7.** Forest plot for improvement in restorative experience (arranged in ascending order by altitude).


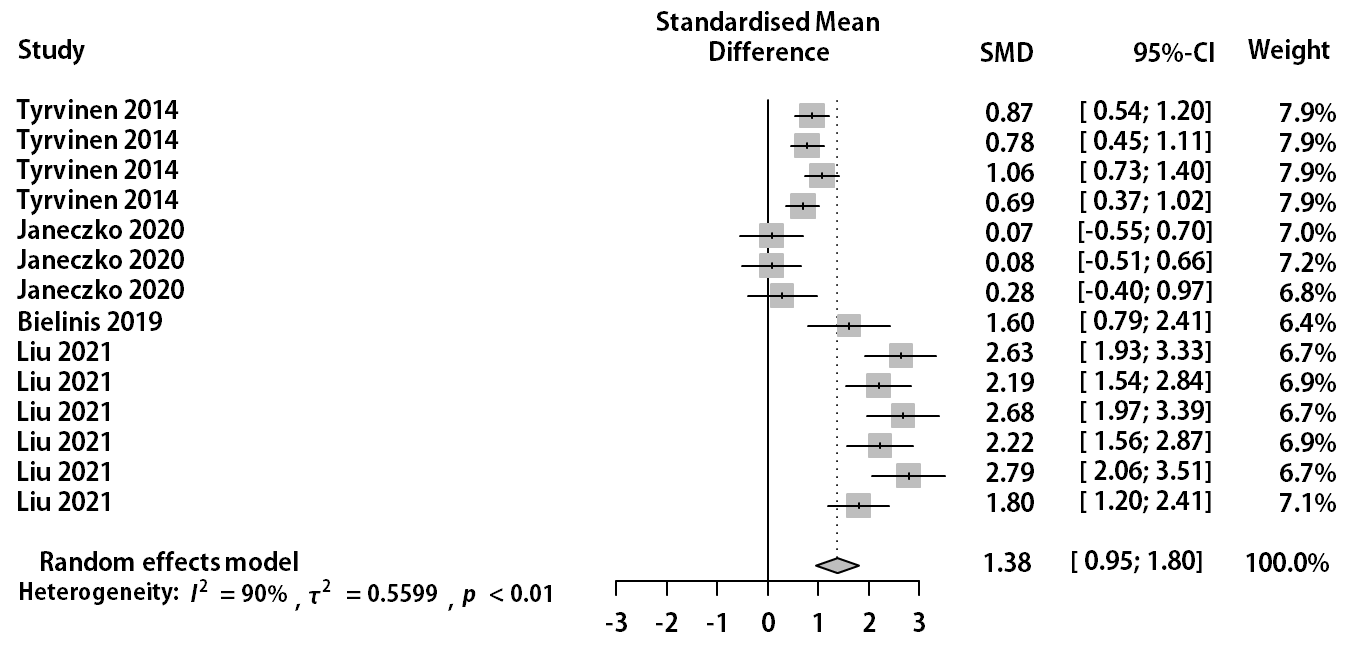


**SUPPLEMENTAL FIGURE S8.** Forest plot for physiological relaxation using diastolic blood pressure (arranged in ascending order by altitude).


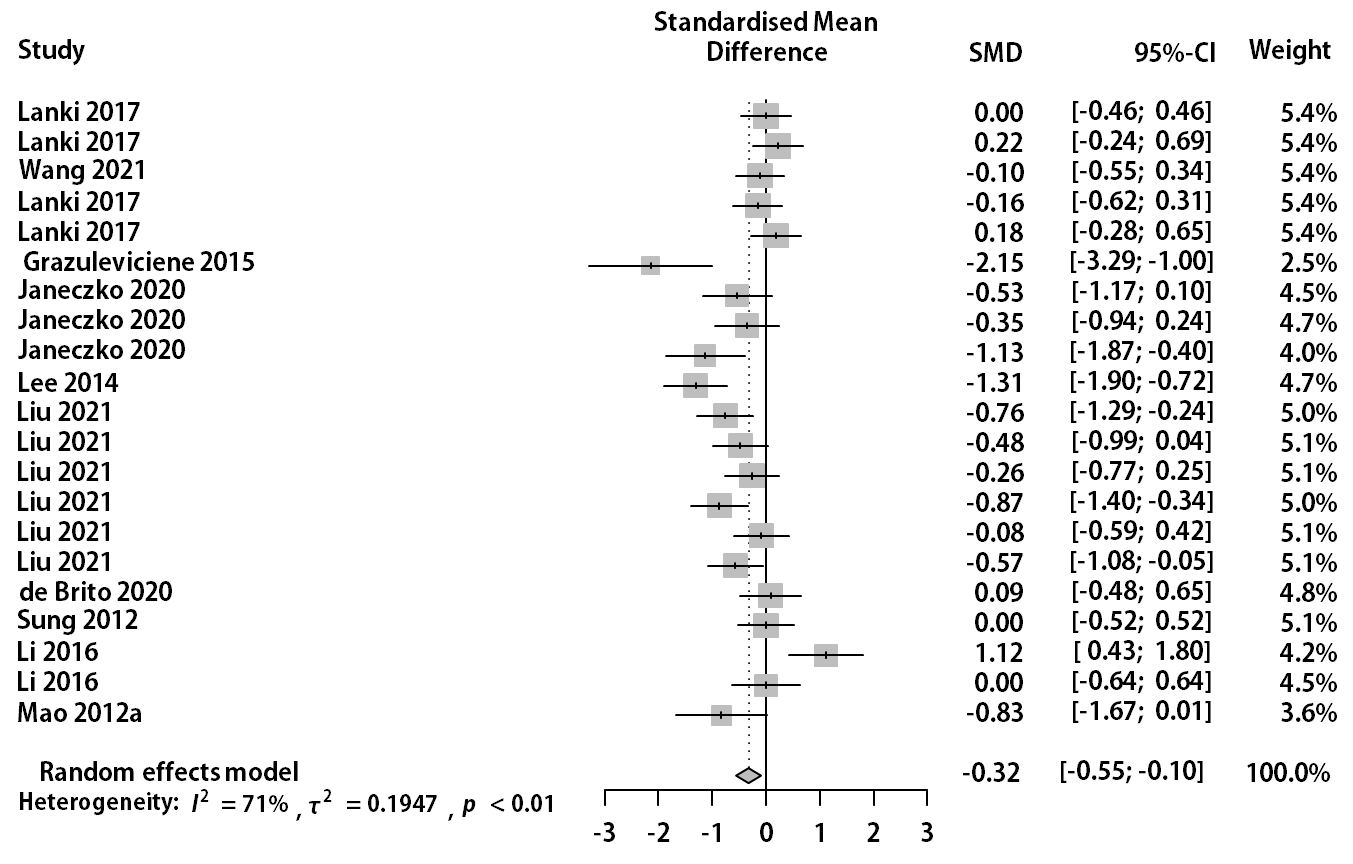


**SUPPLEMENTAL FIGURE S9.** Forest plot for physiological relaxation using systolic blood pressure (arranged in ascending order by altitude).


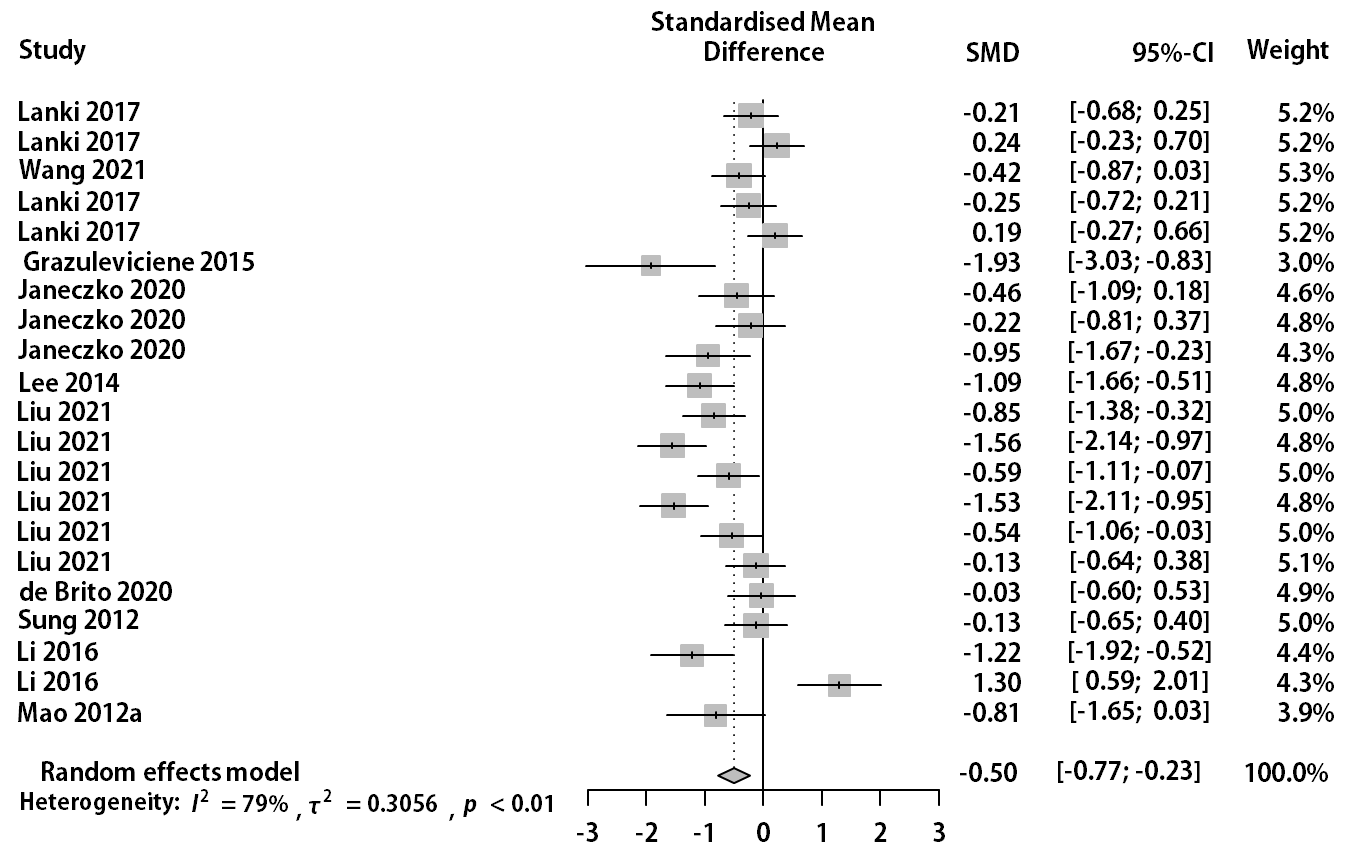


**SUPPLEMENTAL FIGURE S10.** Forest plot for physiological relaxation using heart rate or pulse rate (arranged in ascending order by altitude).

**
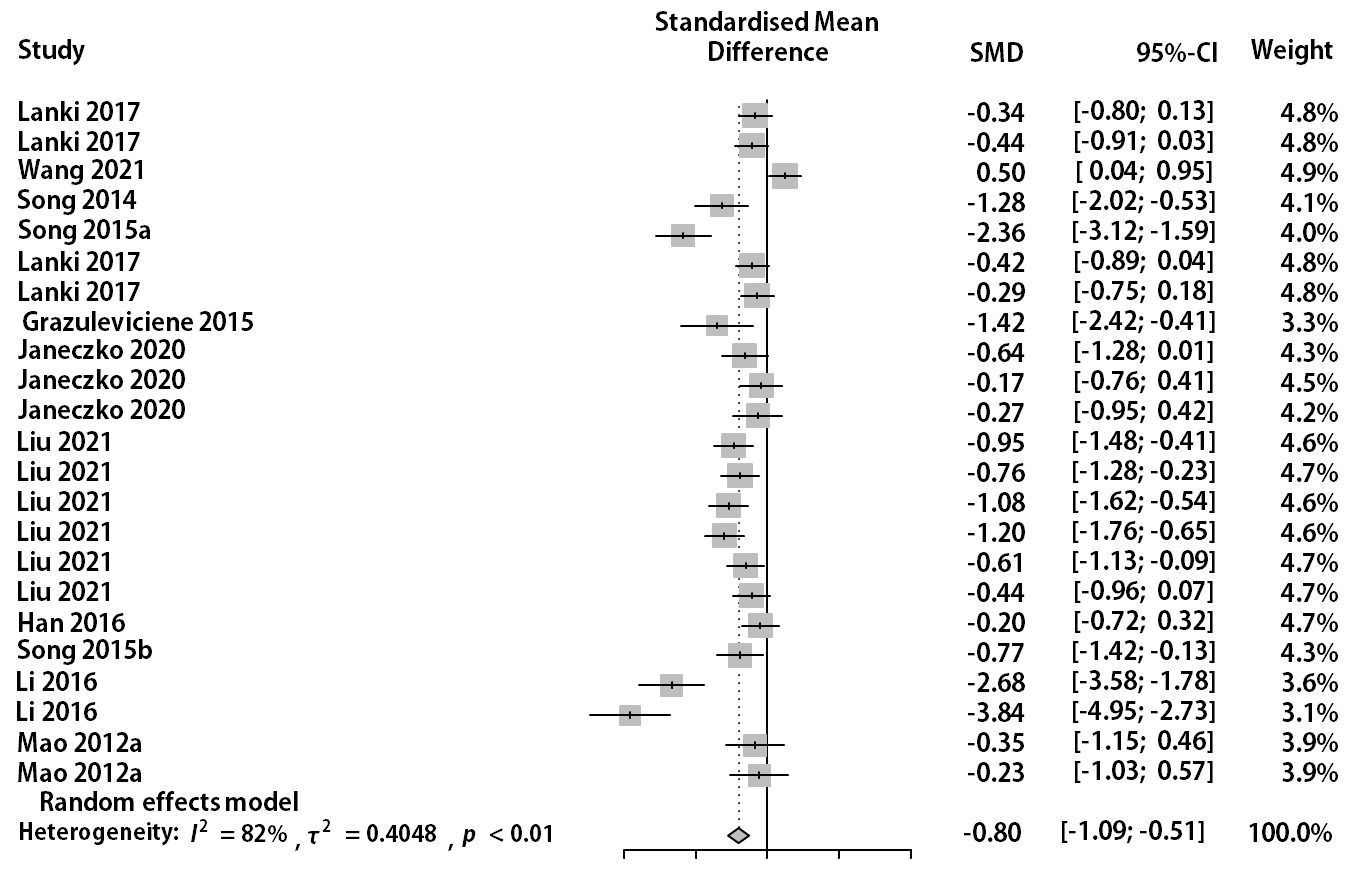
**

**SUPPLEMENTAL FIGURE S11.** Influential diagnostics and sensitivity analysis for the studies examining anxiety.

**
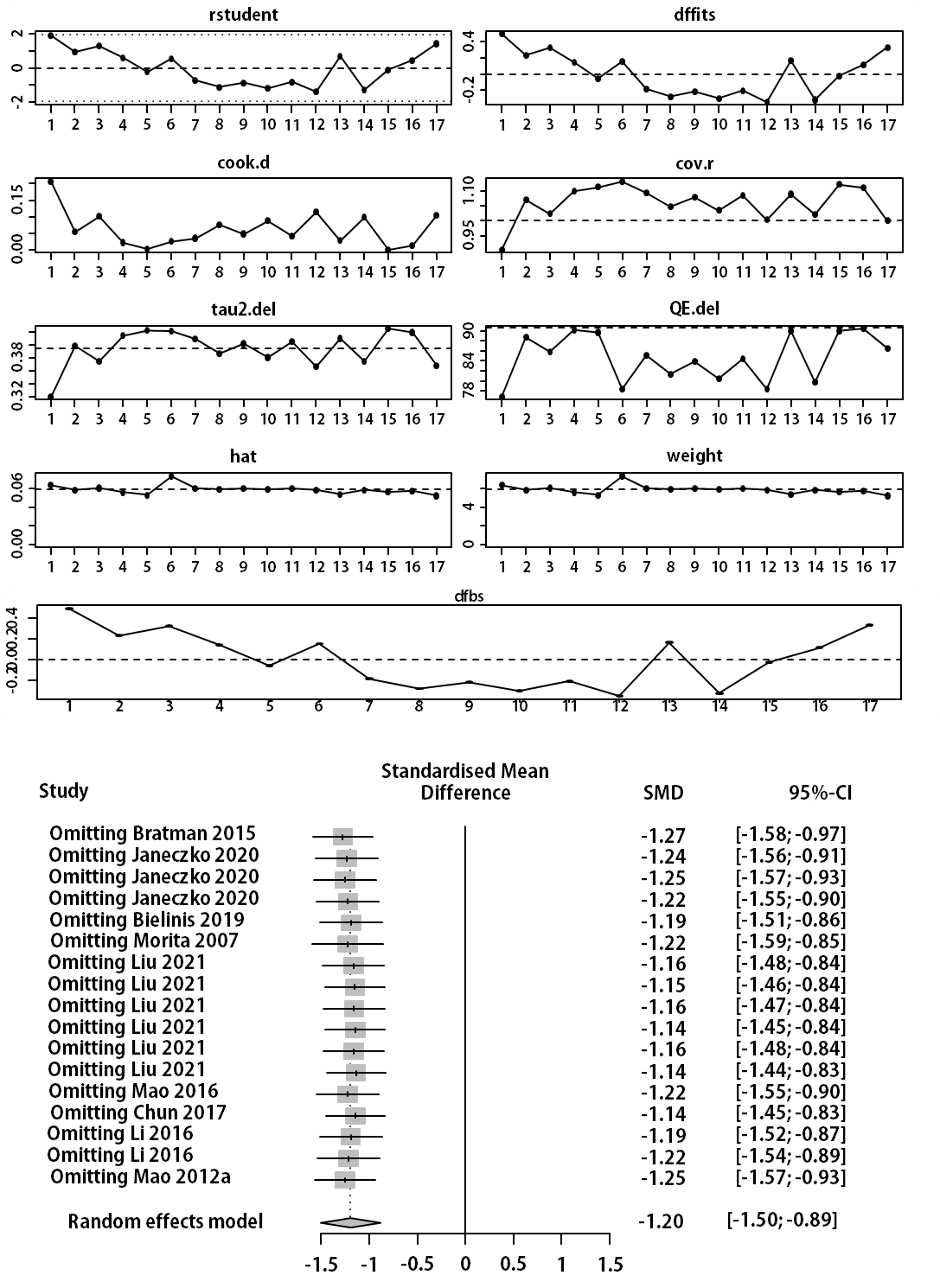
**

**SUPPLEMENTAL FIGURE S12.** Influential diagnostics and sensitivity analysis for the studies examining depression.


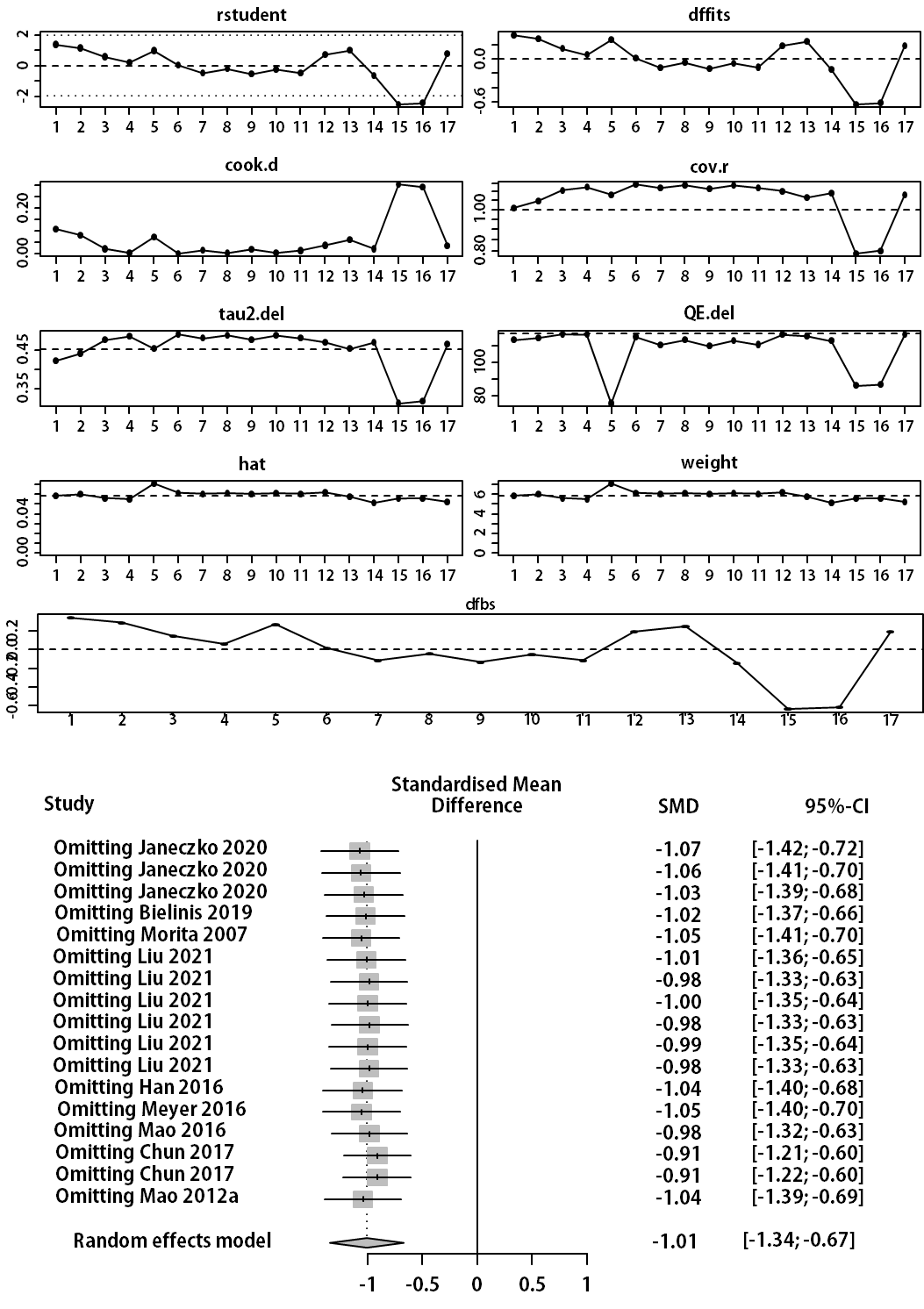


**SUPPLEMENTAL FIGURE S13.** Influential diagnostics and sensitivity analysis for the studies examining confusion.


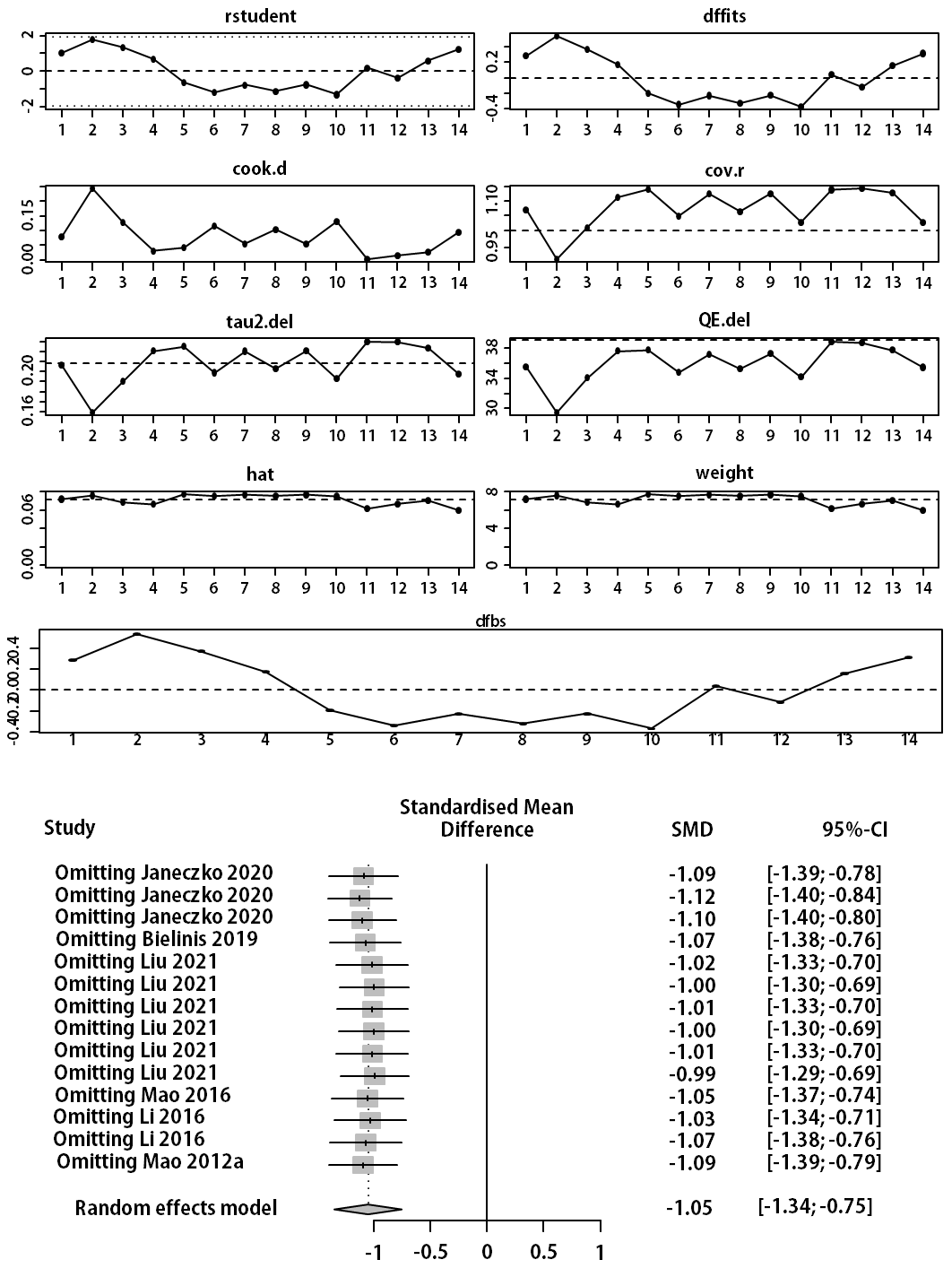


**SUPPLEMENTAL FIGURE S14.** Influential diagnostics and sensitivity analysis for the studies examining fatigue.


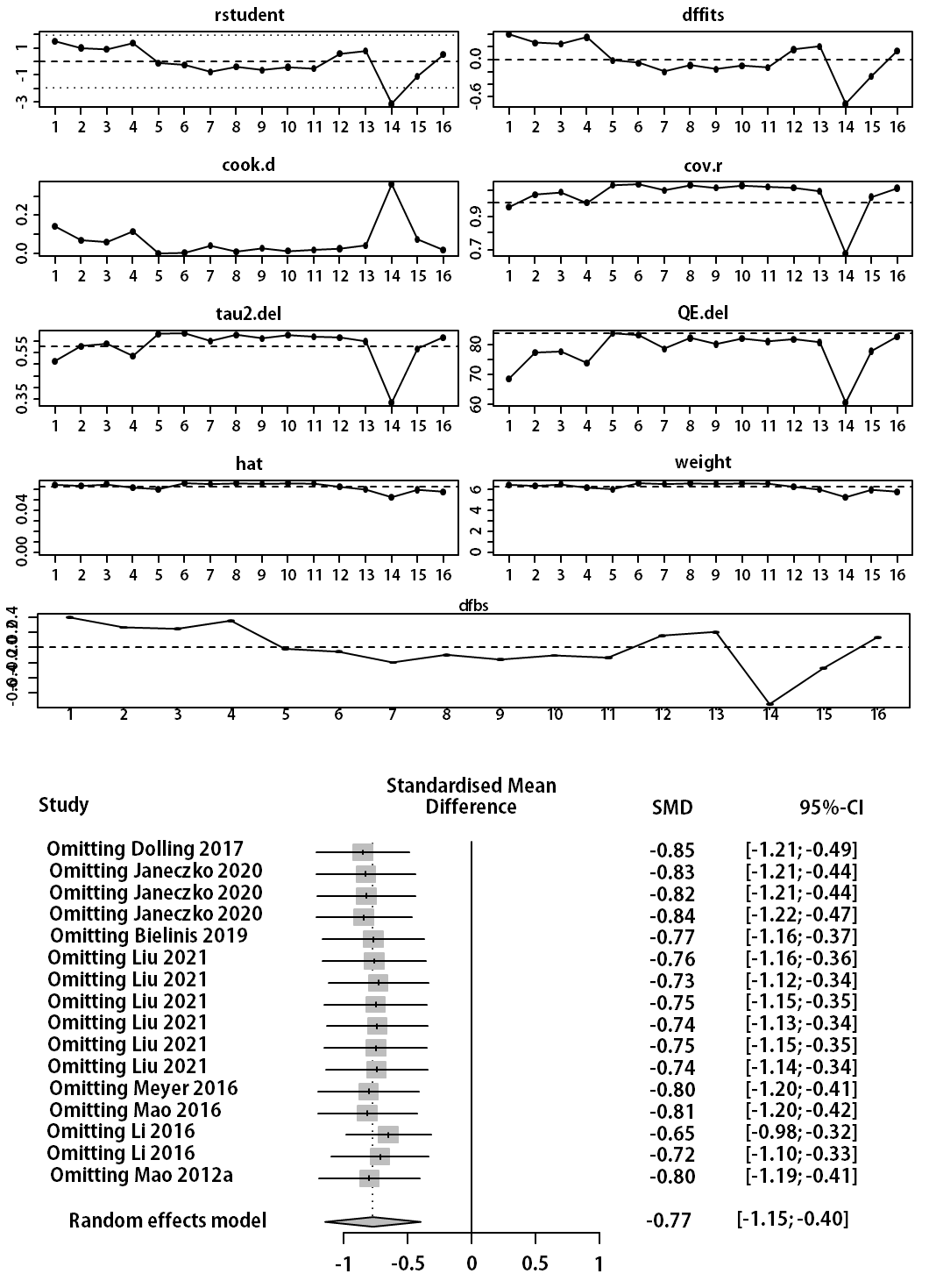


**SUPPLEMENTAL FIGURE S15.** Influential diagnostics and sensitivity analysis for the studies examining hostility.


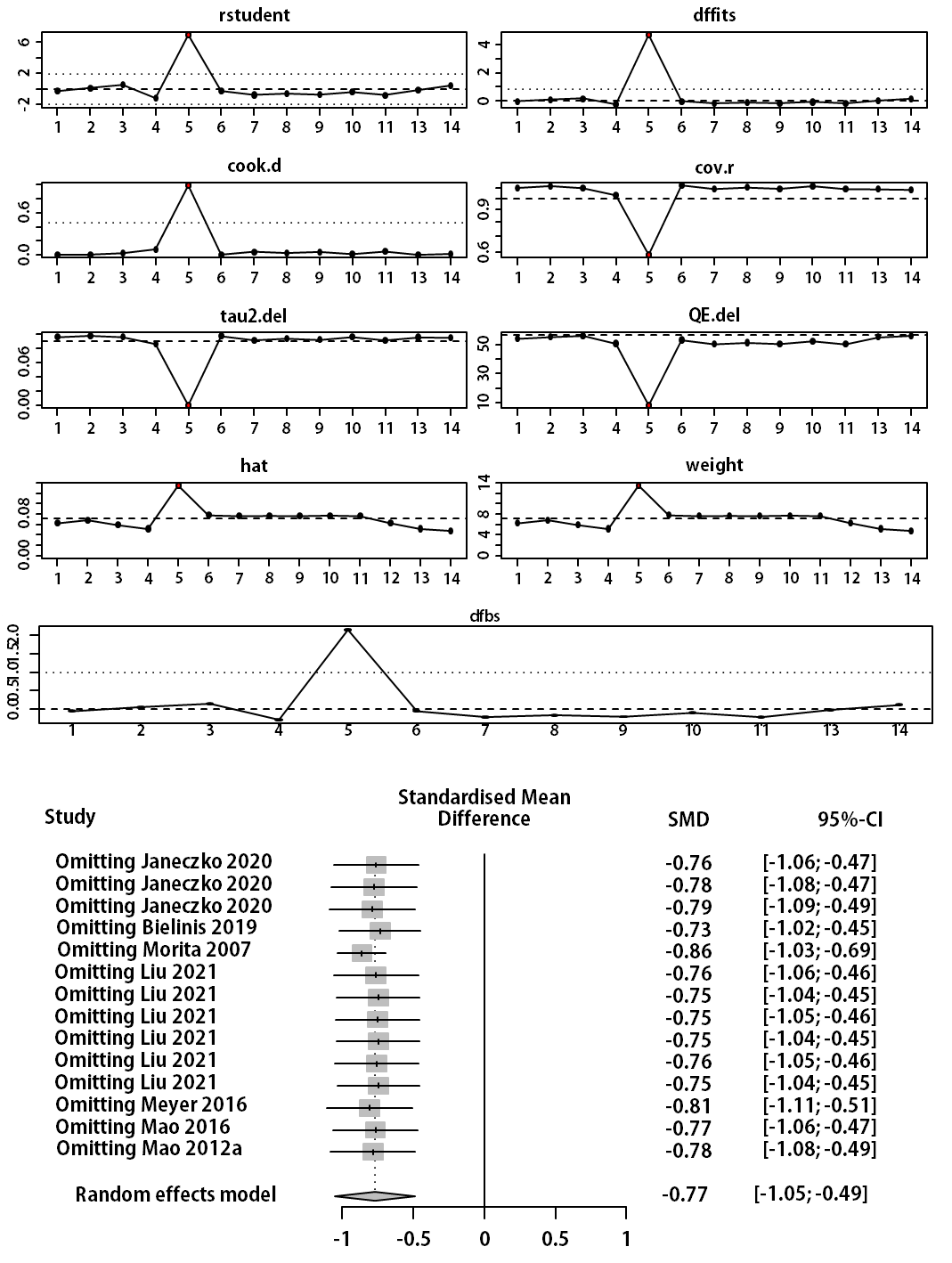


**SUPPLEMENTAL FIGURE S16.** Influential diagnostics and sensitivity analysis for the studies examining vitality.


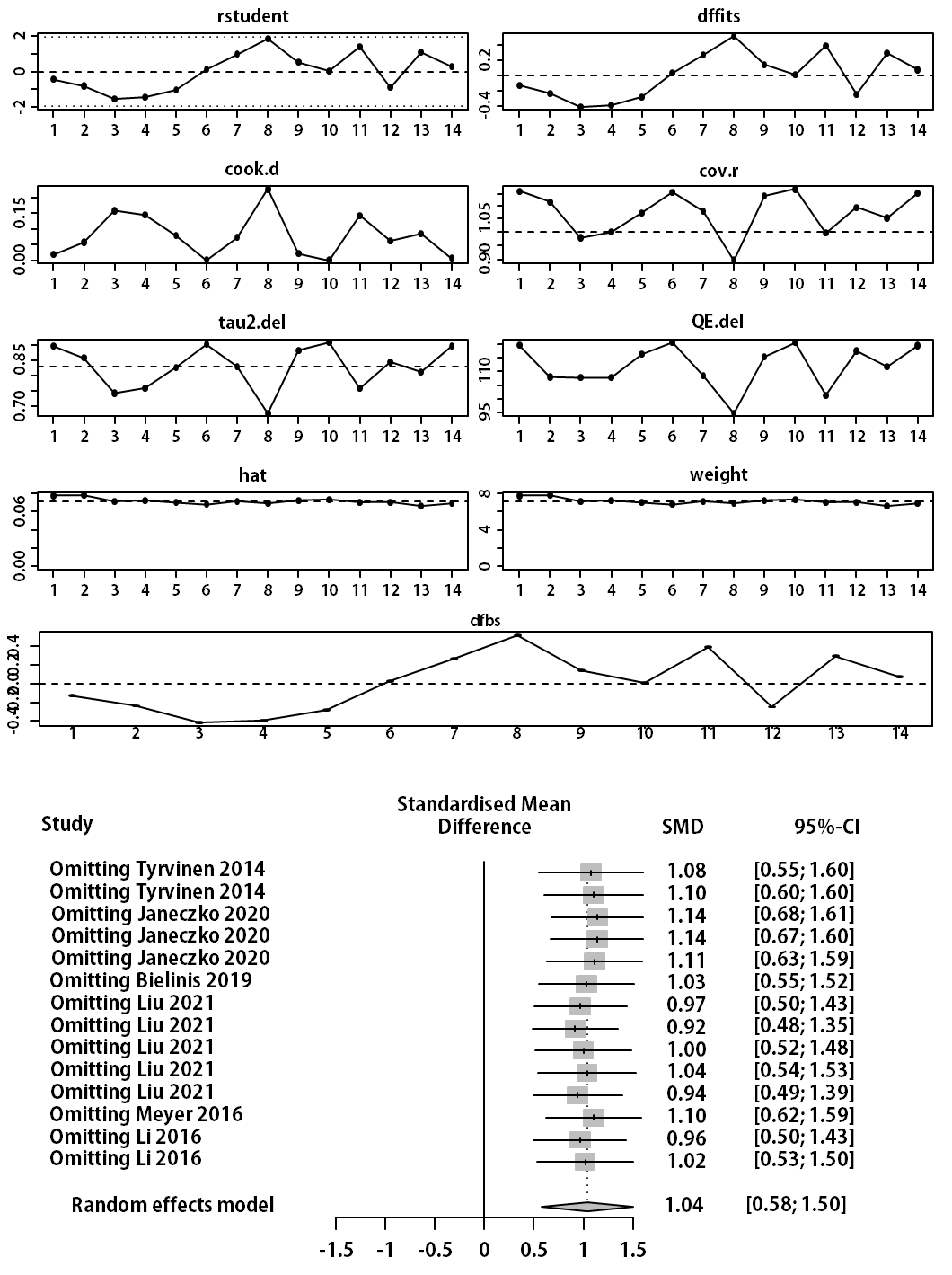


**SUPPLEMENTAL FIGURE S17.** Influential diagnostics and sensitivity analysis for the studies examining restorative experience(ROS).


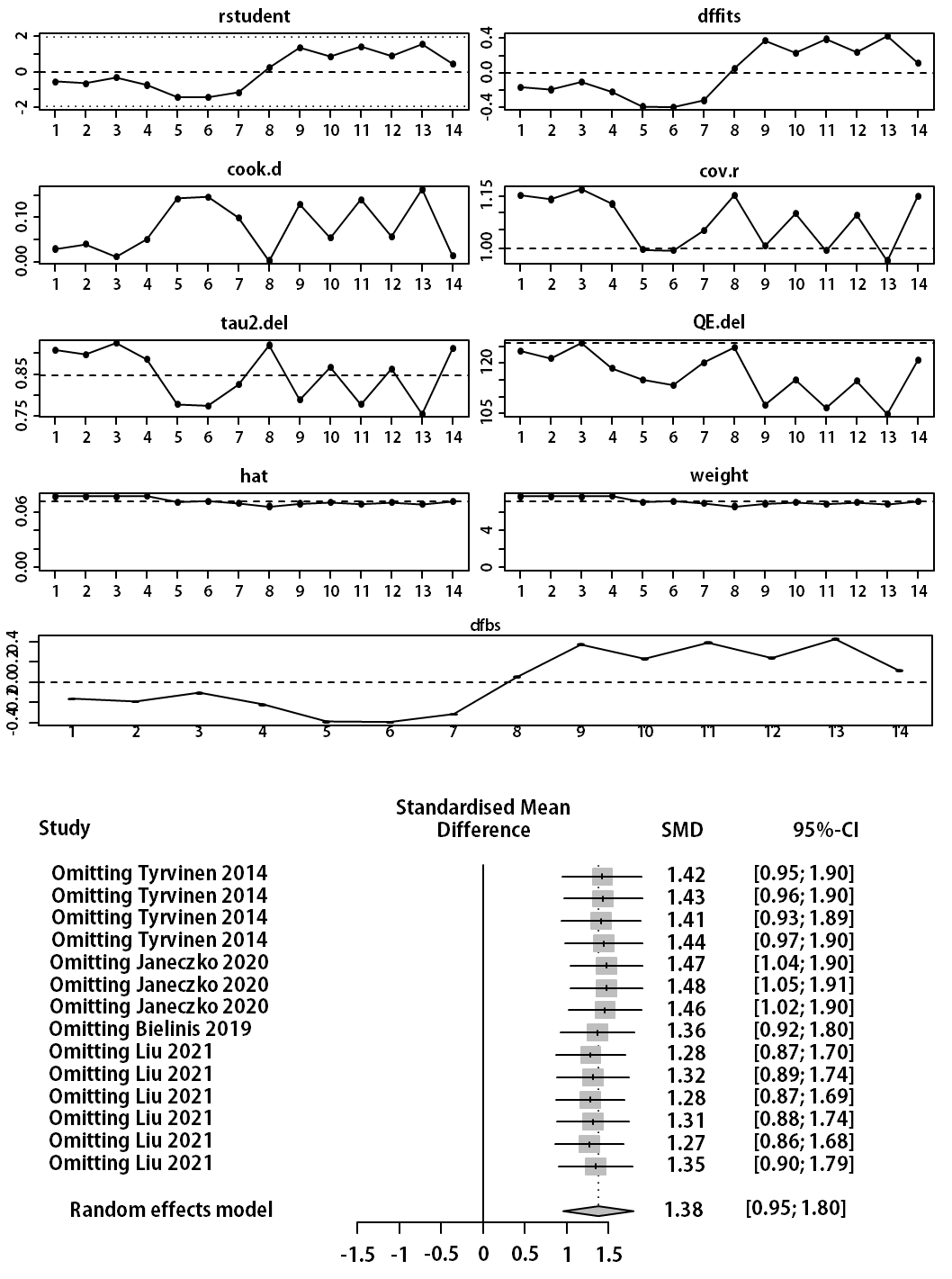


**SUPPLEMENTAL FIGURE S18.** Influential diagnostics and sensitivity analysis for the studies examining diastolic blood pressure.

**
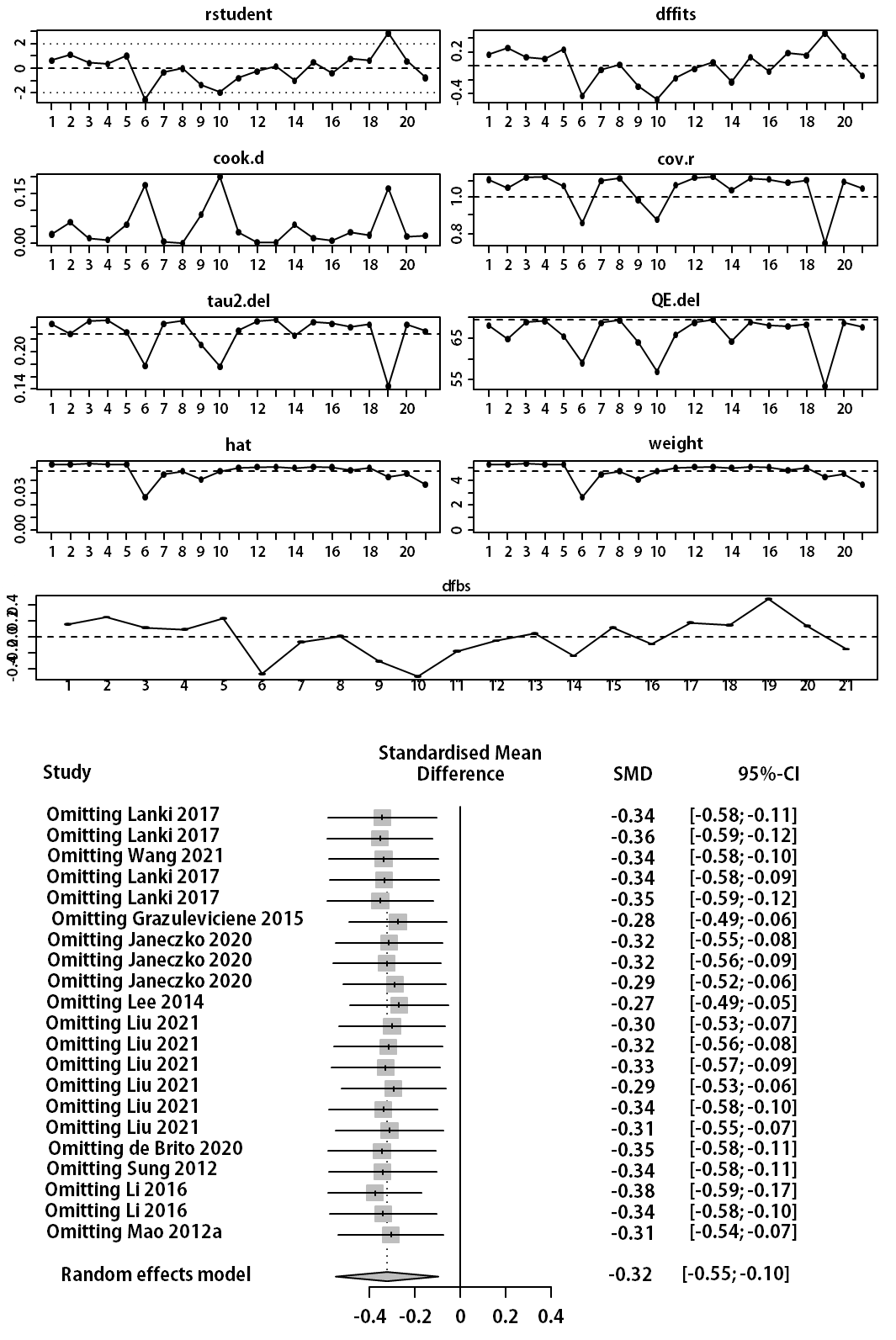
**

**SUPPLEMENTAL FIGURE S19.** Influential diagnostics and sensitivity analysis for the studies examining systolic blood pressure.

**
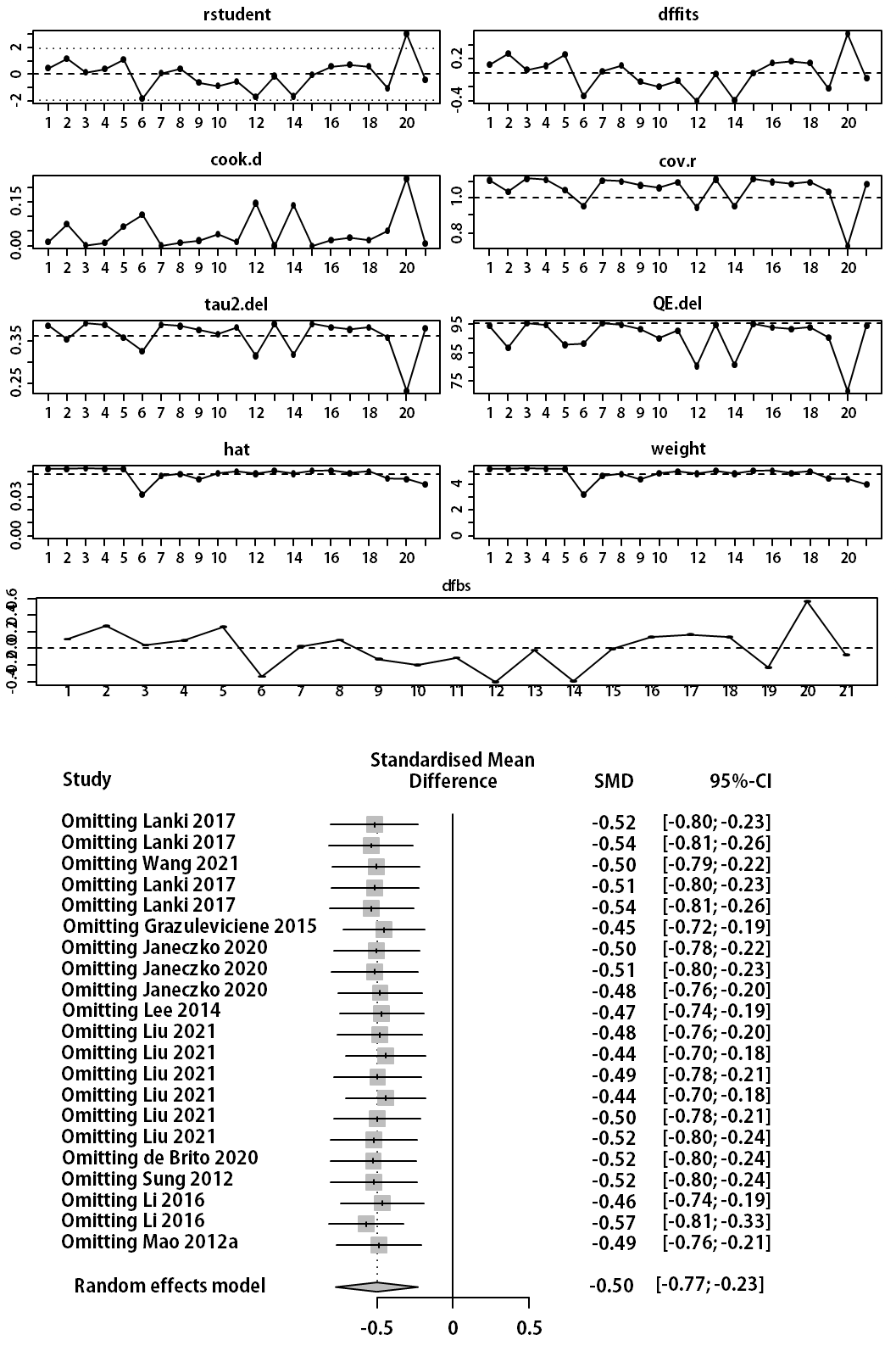
**

**SUPPLEMENTAL FIGURE S20.** Influential diagnostics and sensitivity analysis for the studies examining heart rate or pulse rate.


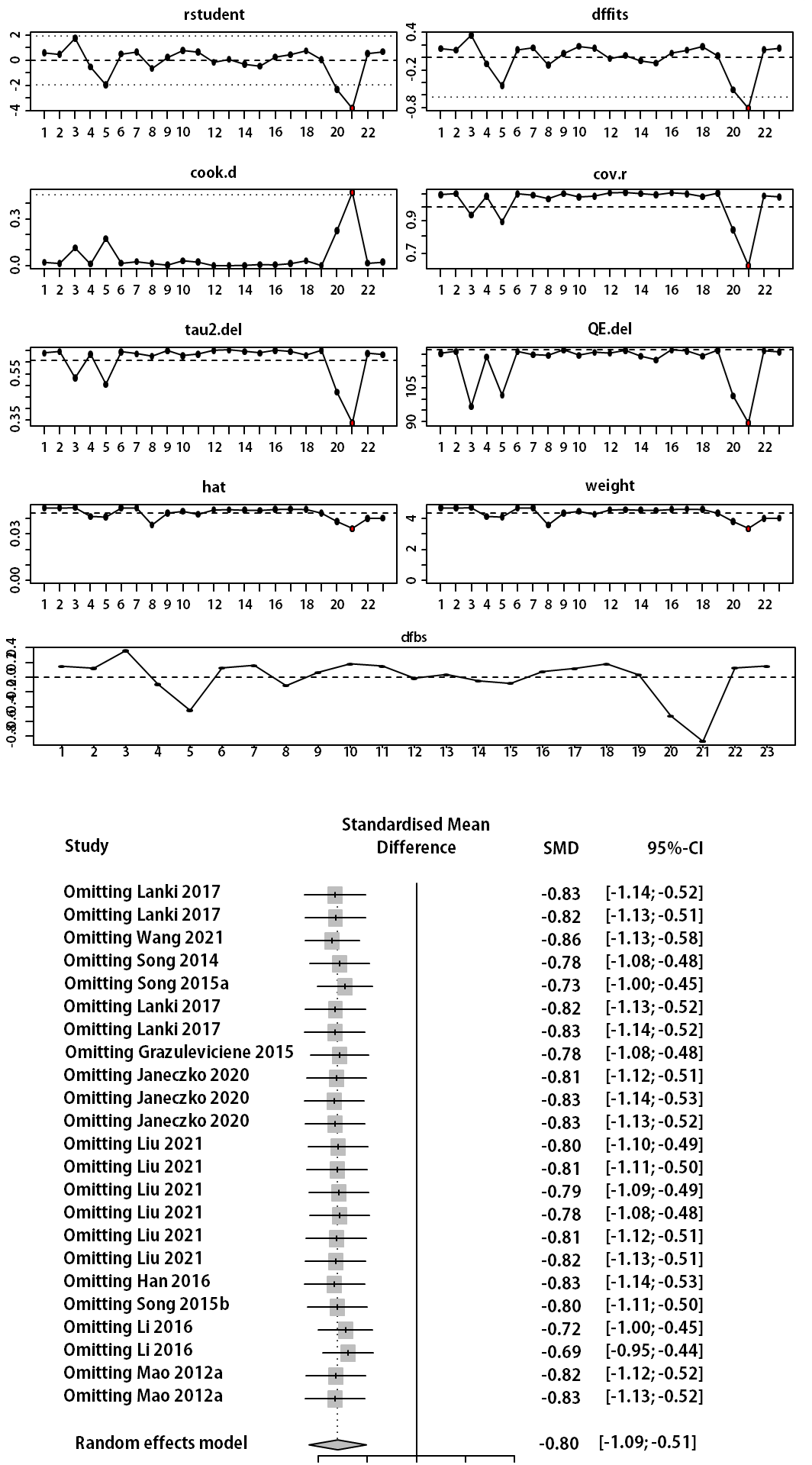


**SUPPLEMENTAL FIGURE S21.** Funnel plots including all studies (random effects model).

| **Anxiety** | **Depression** | **Confusion** | **Fatigue** |
| --- | --- | --- | --- |
| 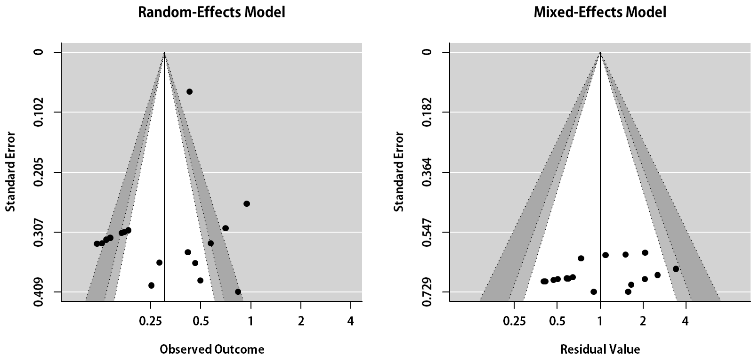  Egger’s regression test of funnel plot asymmetry:  t =-1.54, df =15, p-value = 0.1449  Sample estimates:  bias=-1.4081; SE=0.9156; intercept= 0.1952 | 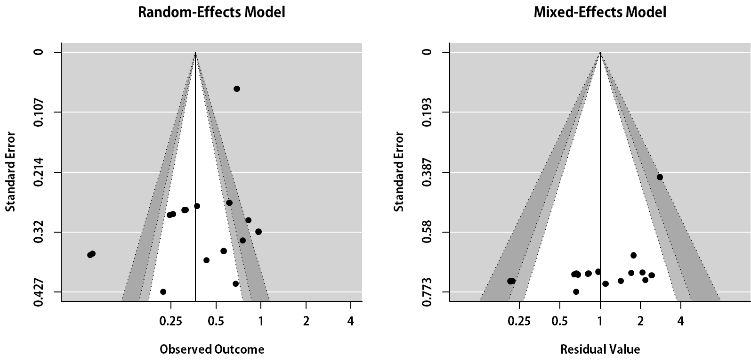  Egger’s regression test of funnel plot asymmetry:  **t = -2.97, df = 15, p-value = 0.0096**  Sample estimates:  bias= -2.6472; SE=0.8921; intercept= -0.2013 | 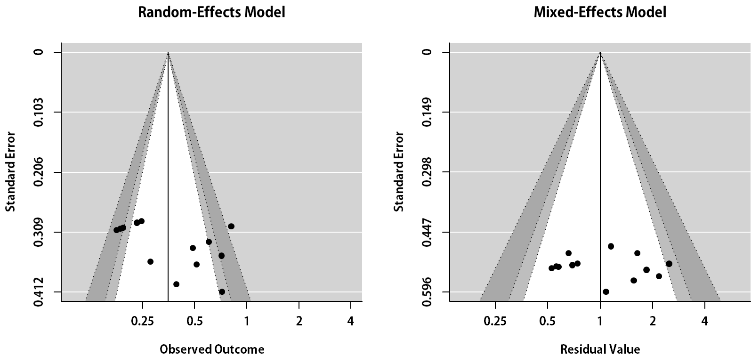  Egger’s regression test of funnel plot asymmetry:  t = 2.05, df = 12, p-value = 0.0631  Sample estimates:  bias= 7.7927; SE=3.8044; intercept= 1.2342 | 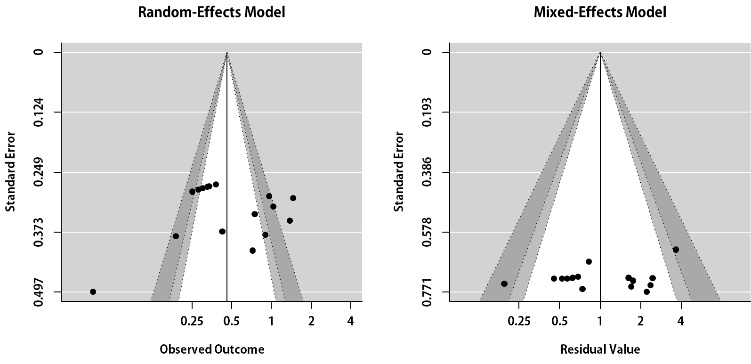  Egger’s regression test of funnel plot asymmetry:  t = -0.34, df = 14, p-value = 0.7356  Sample estimates:  bias= -1.3605; SE= 3.9498; intercept= 1.2648 |
| **Hostility** | **Vitality** | **Restorative experience (ROS)** |  |
| 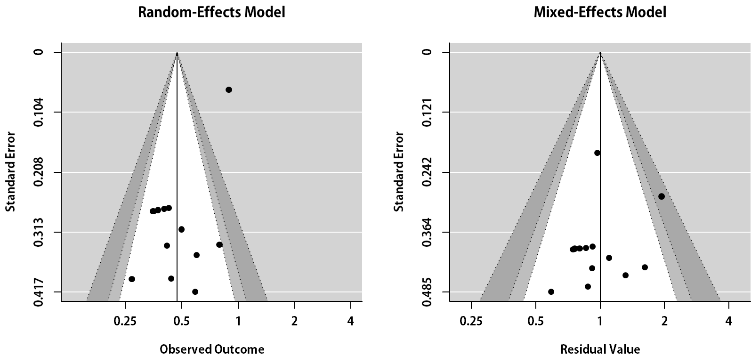  Egger’s regression test of funnel plot asymmetry:  **t =- 6.00, df = 12, p-value < .000**  Sample estimates:  bias=- 2.8181; SE= 0.4697; intercept= 0.0458 | 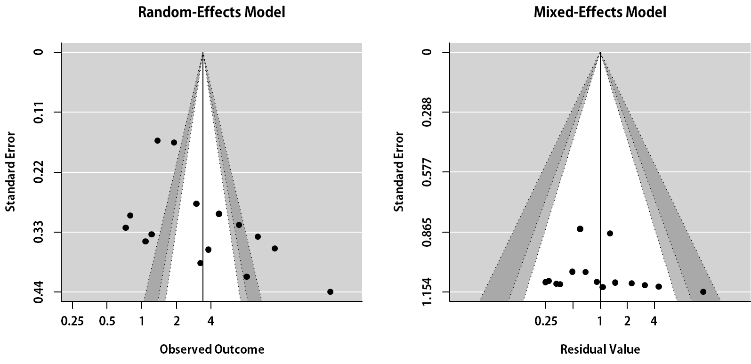  Egger’s regression test of funnel plot asymmetry:  **t = 2.22, df = 13, p-value = 0.0446**  Sample estimates:  Bias= 5.4574; SE= 2.4557; intercept= -0.5388 | 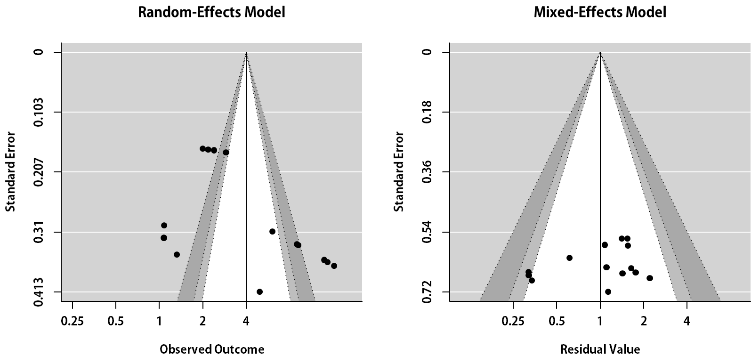  Egger’s regression test of funnel plot asymmetry:  **t = 2.22, df = 12, p-value = 0.0464**  Sample estimates:  bias= 4.7492; SE= 2.1388; intercept= 0.0050 |  |
| **DBP** | **SBP** | **HR/PR** |  |
| 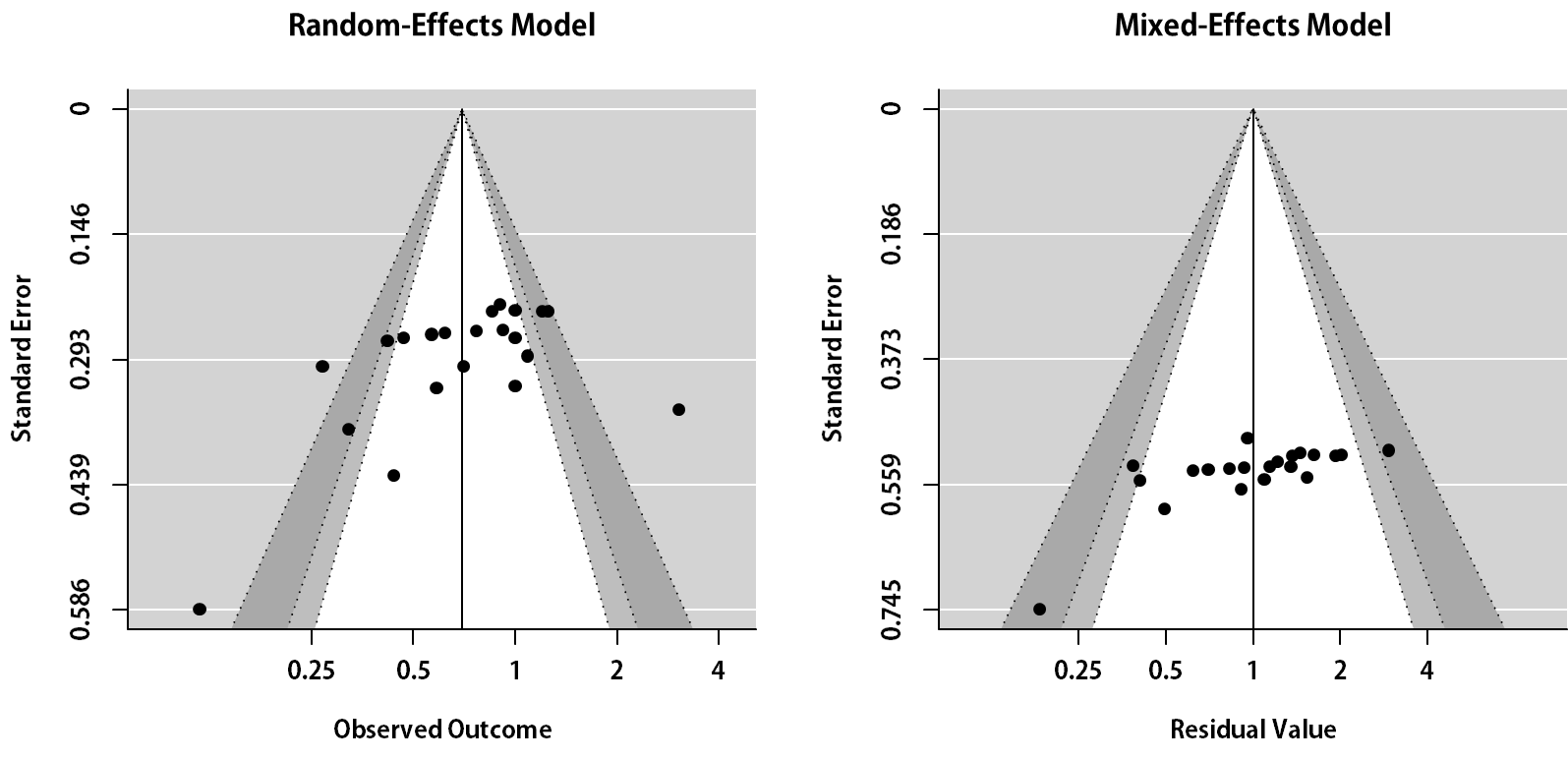  Egger’s regression test of funnel plot asymmetry:  t = -2.11, df = 19, p-value = 0.0779  Sample estimates:  bias= -4.1766; SE= 1.9755; intercept= 0.8783 | 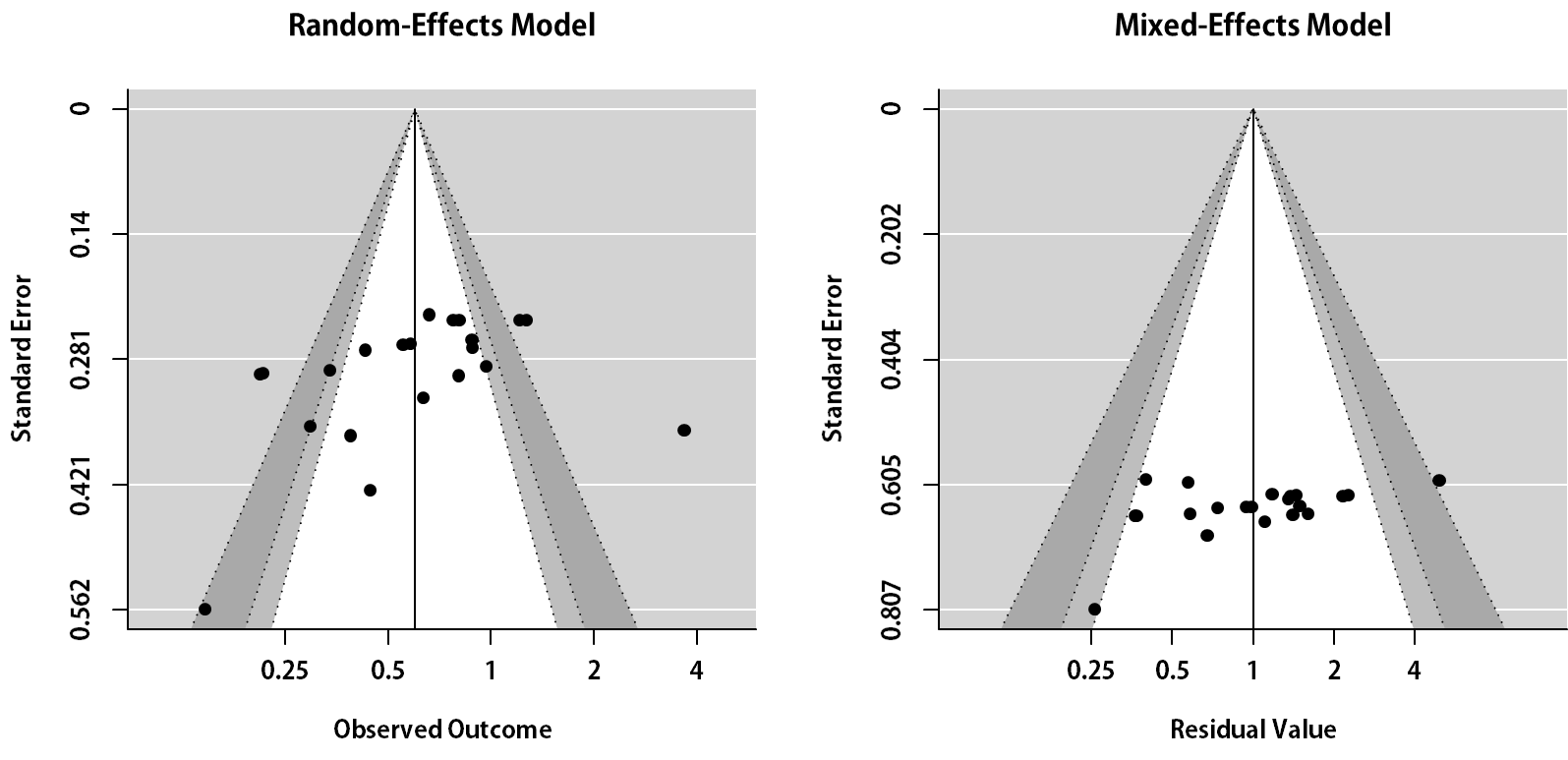  Egger’s regression test of funnel plot asymmetry:  t = -1.76, df = 19, p-value = 0.00940  Sample estimates:  bias= -4.2156; SE= 2.13912; intercept= 0.7443 | 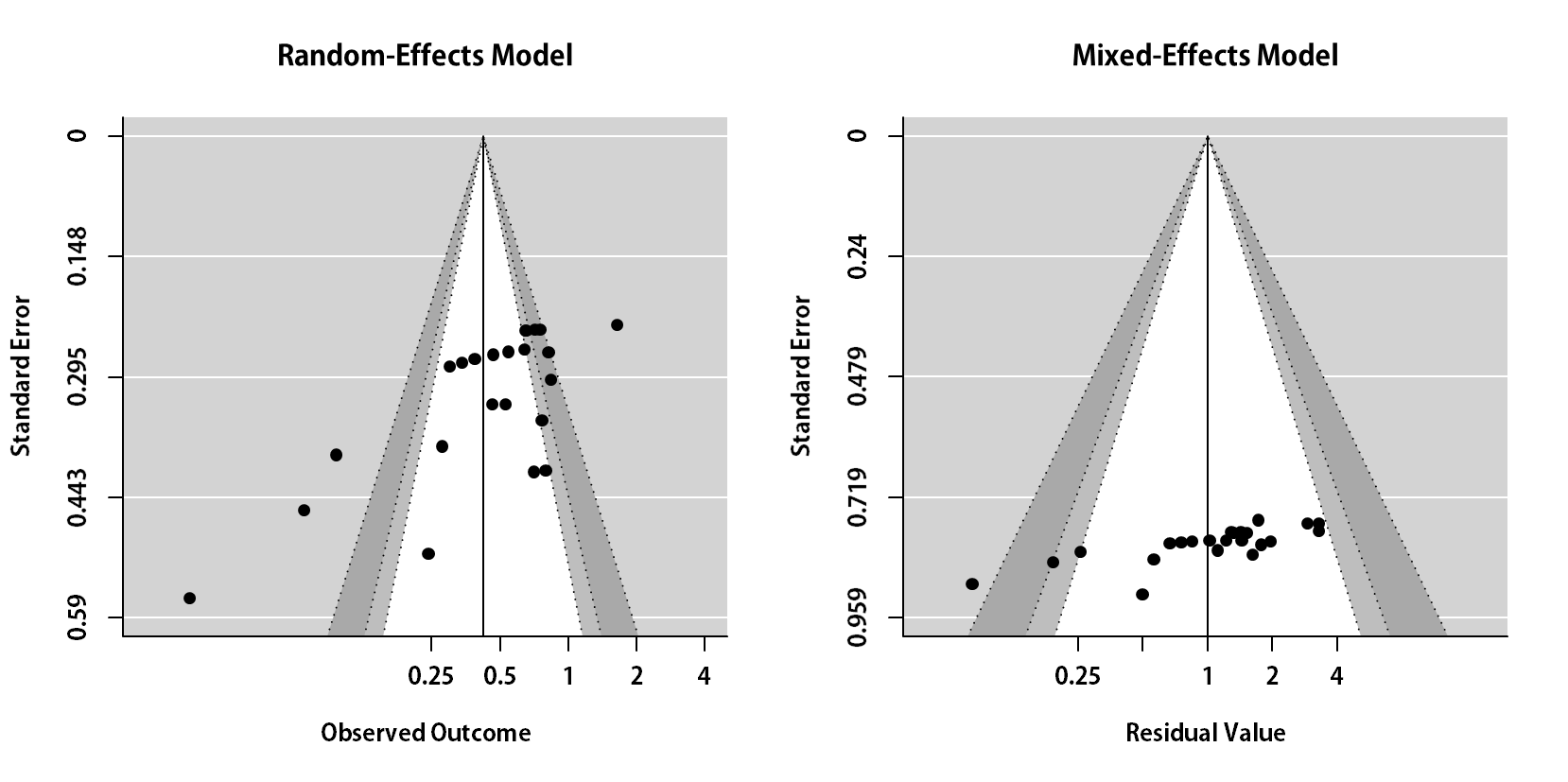  Egger’s regression test of funnel plot asymmetry:  **t = -4.13, df = 21, p-value = 0.0005**  Sample estimates:  bias= -6.7071; SE= 1.6235; intercept= 1.3173 |  |

**SUPPLEMENTAL FIGURE S22.** Funnel plots after the exclusion of studies with high risk of bias (random effects model).

| **Anxiety** | **Depression** | **Confusion** | **Fatigue** |
| --- | --- | --- | --- |
| 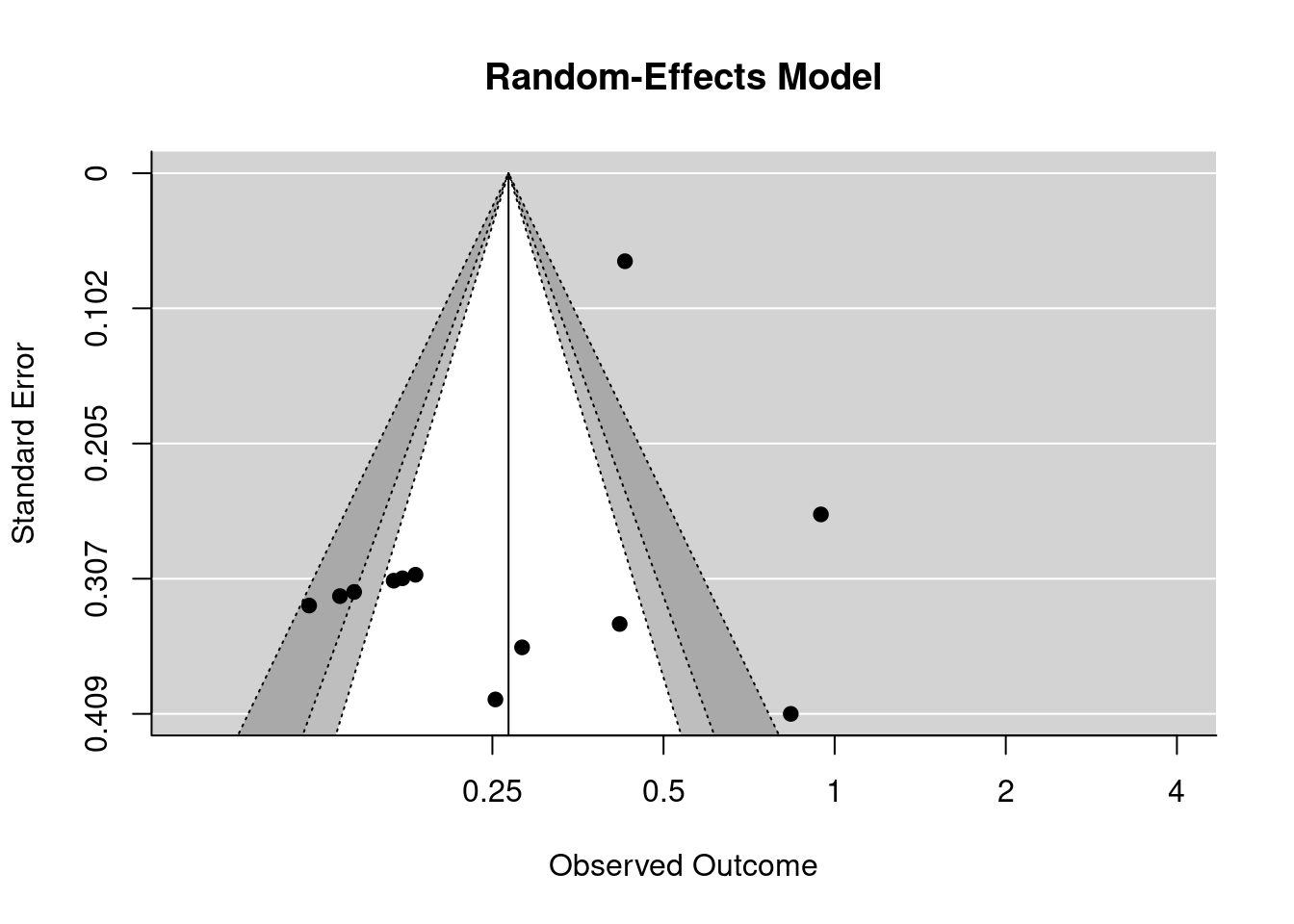  Egger’s regression test of funnel plot asymmetry:  t = -1.91, df =10, p-value = 0.0848  Sample estimates:  bias= -2.0077; SE= 1.0496; intercept= 0.7154 | 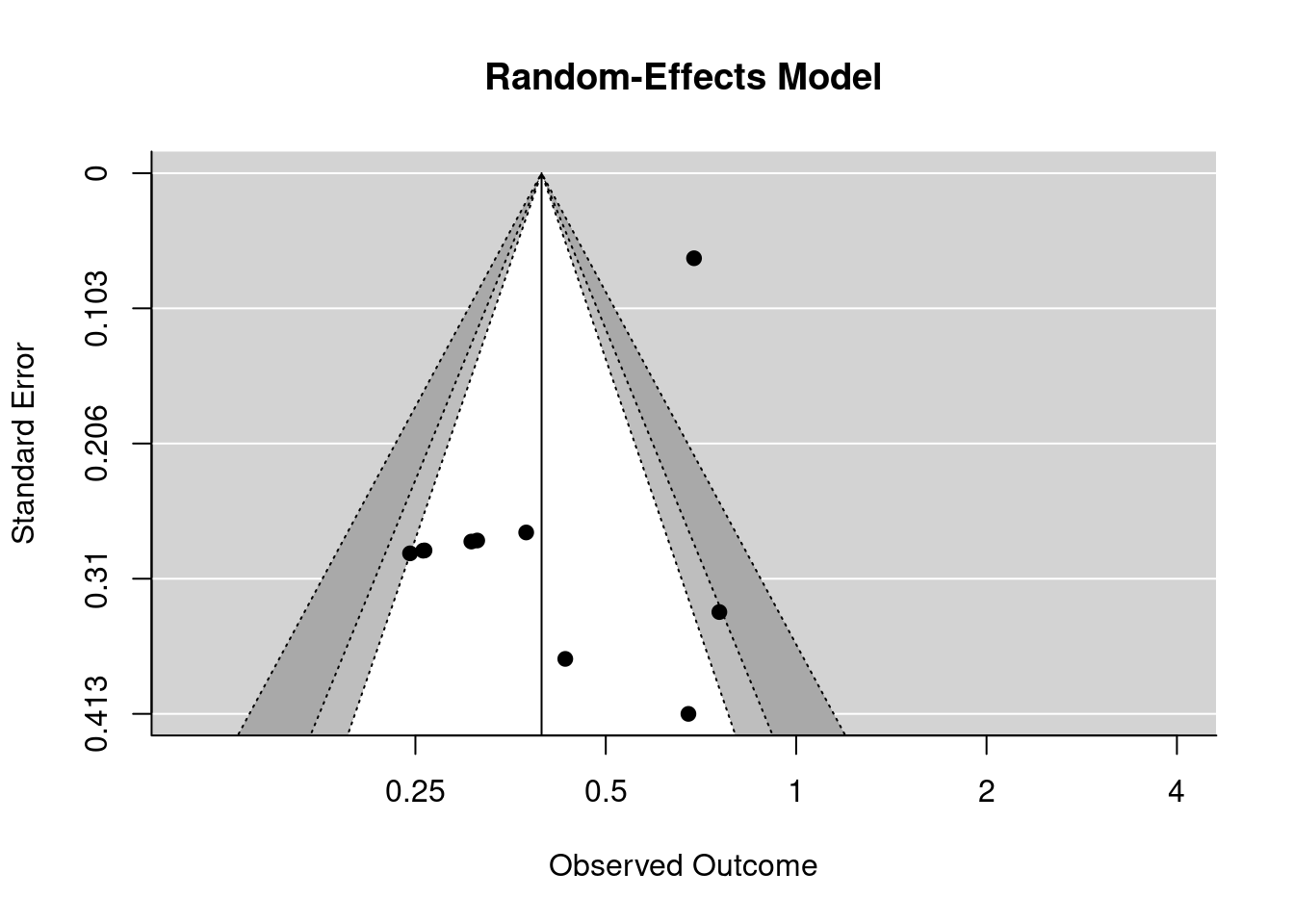  Egger’s regression test of funnel plot asymmetry:  **t = -3.39, df = 8, p-value = 0.0095**  Sample estimates:  bias= -2.5778; SE=0.7606; intercept= -0.2278 | 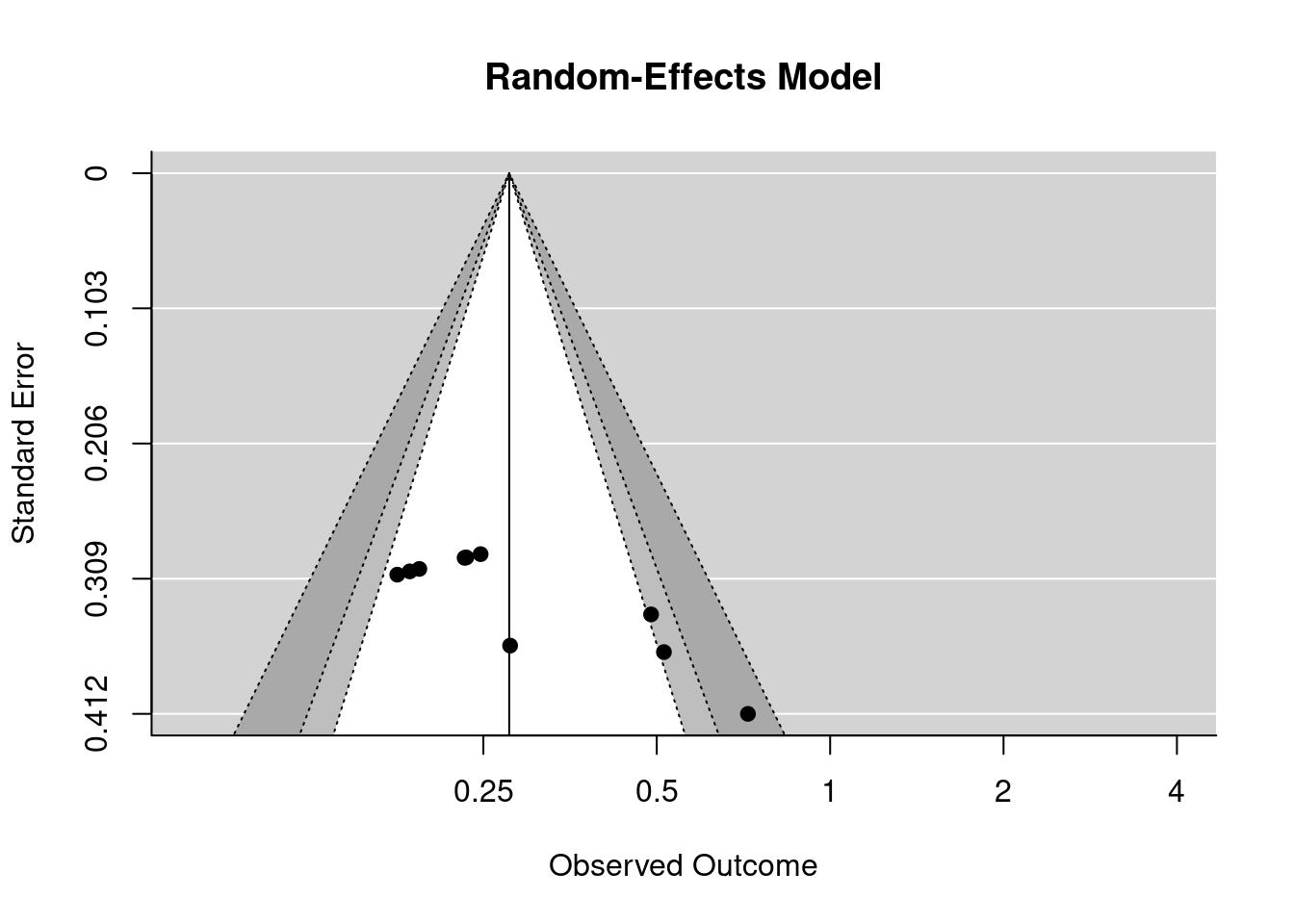  Egger’s regression test of funnel plot asymmetry:  **t = 4.17, df = 8, p-value = 0.0031**  Sample estimates:  bias= 10.0782; SE= 2.4176; intercept= -4.5177 | 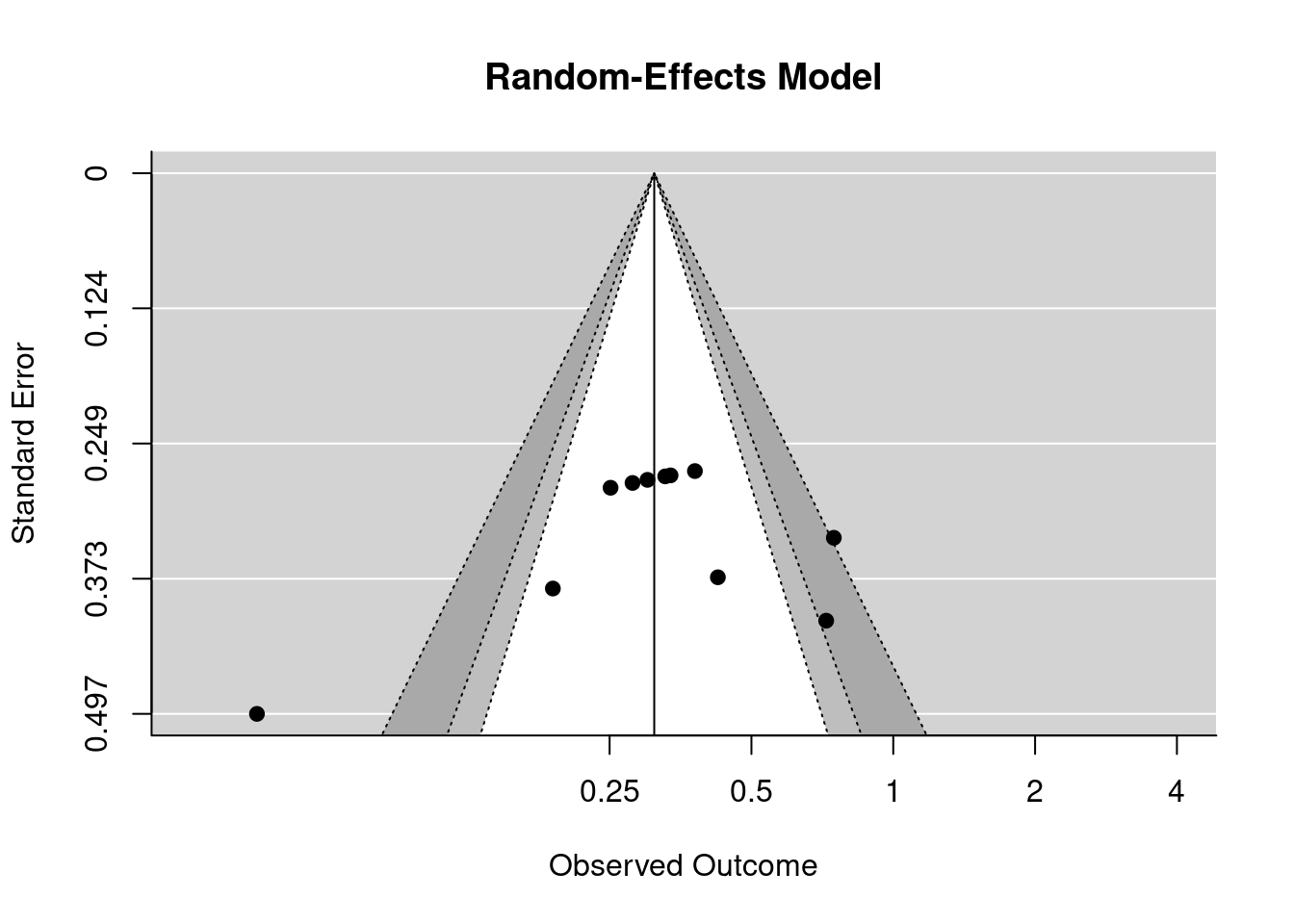  Egger’s regression test of funnel plot asymmetry:  t = -0.93 df = 9, p-value = 0.3764  Sample estimates:  bias= -2.7916; SE=2.9999; intercept= -0.2591 |
| **Hostility** | **Vitality** | **Restorative experience (ROS)** |  |
| 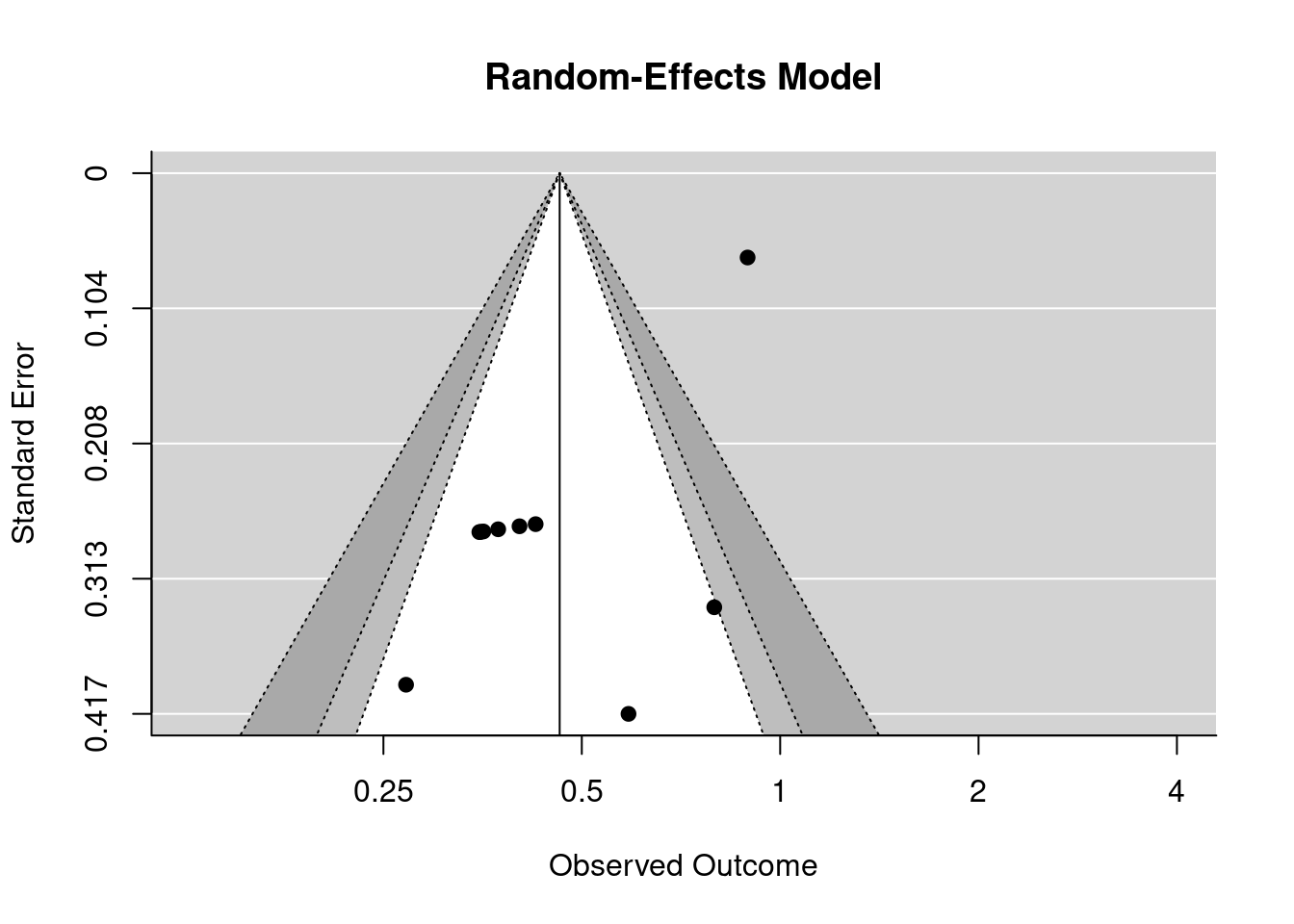  Egger’s regression test of funnel plot asymmetry:  **t = -5.33, df = 8, p-value = 0.0007**  Sample estimates:  bias= -3.1869; SE= 0.5984; intercept= 0.0754 | N.A.  Cases less than 10 | N.A.  Cases less than 10 |  |
| **DBP** | **SBP** | **HR/PR** |  |
| 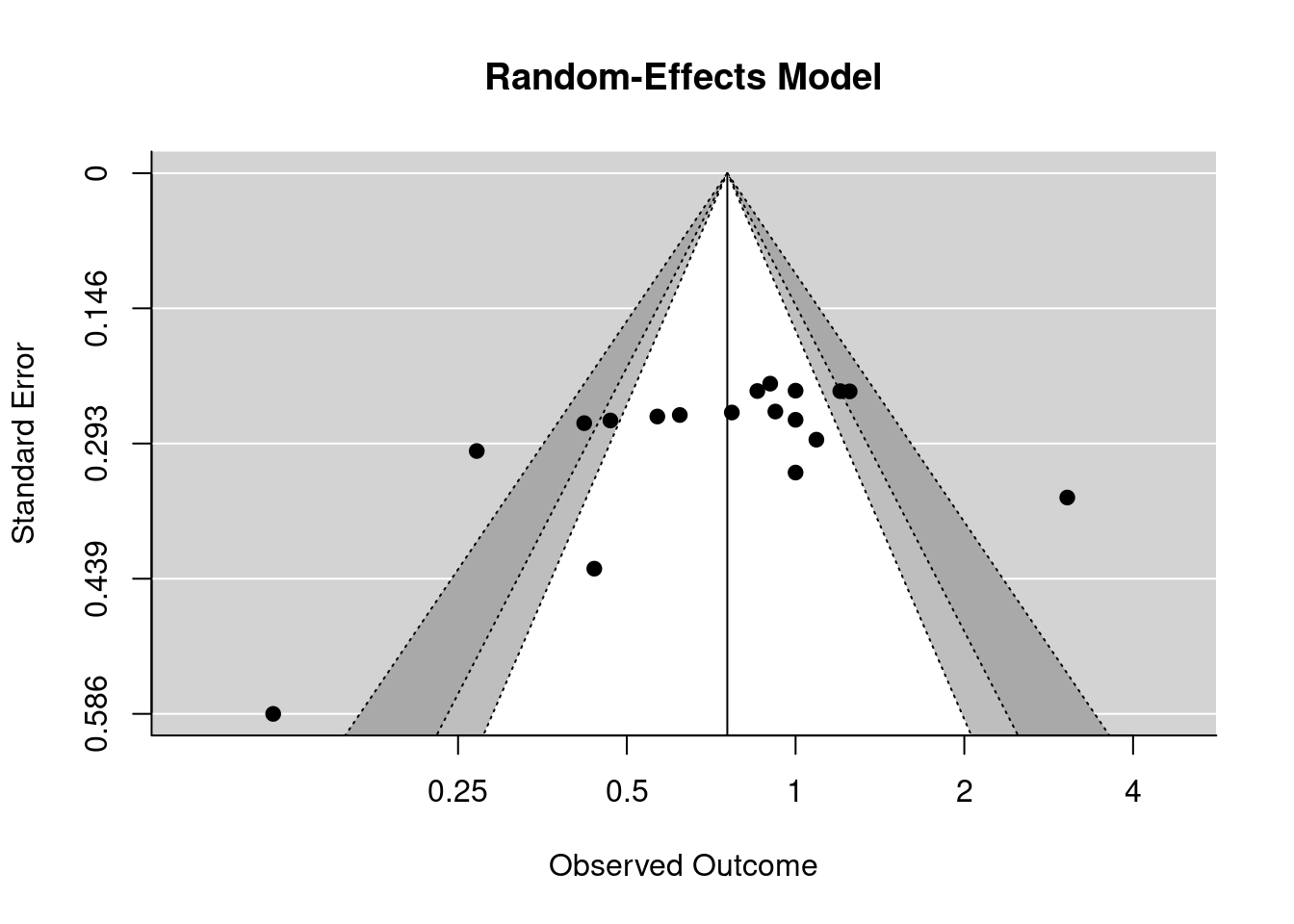  Egger’s regression test of funnel plot asymmetry:  t = -1.58, df = 16, p-value = 0.1336  Sample estimates:  bias= -3.6297; SE= 2.2966; intercept=0.7452 | 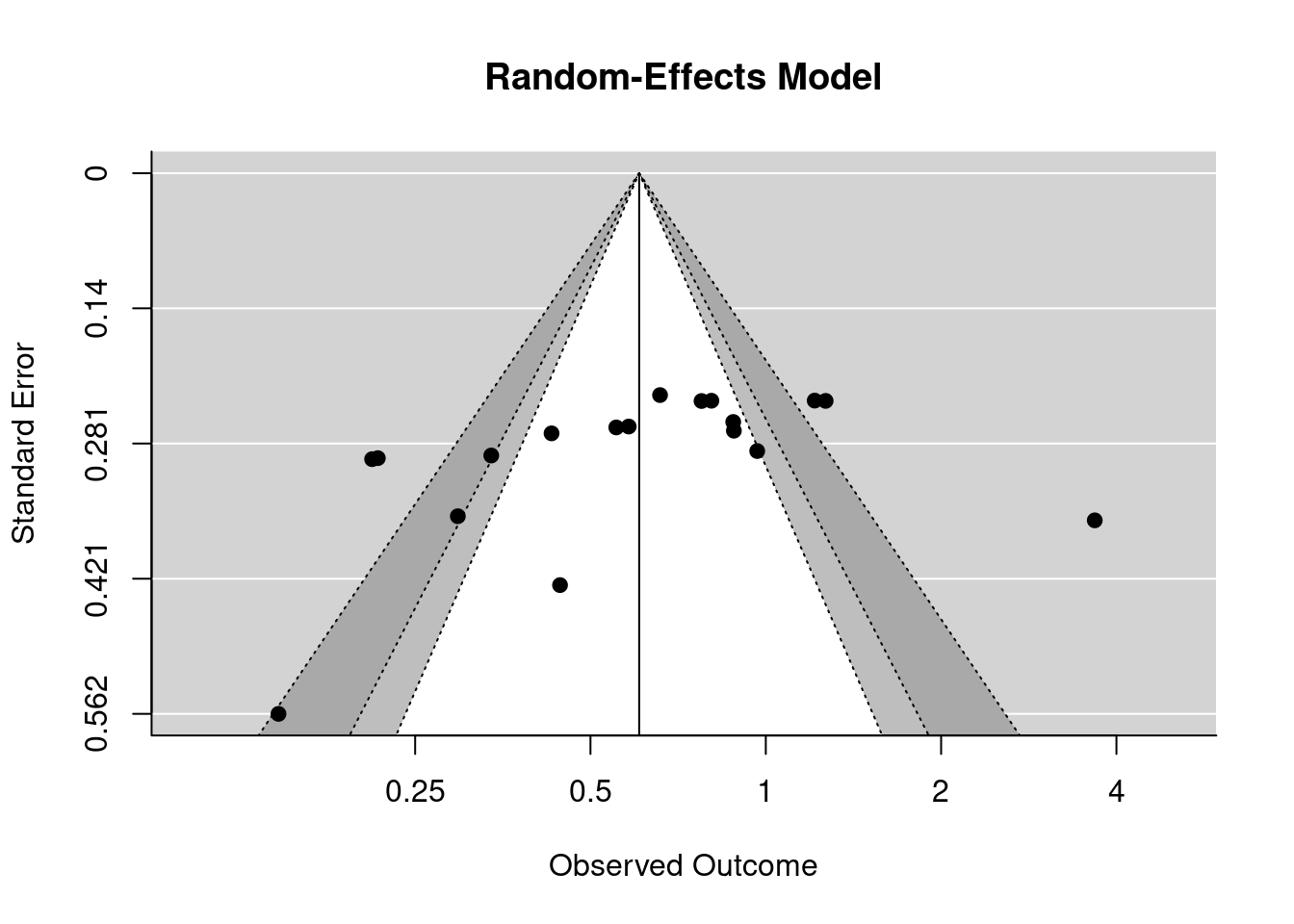  Egger’s regression test of funnel plot asymmetry:  t = -1.57, df = 16, p-value = 0.1363  Sample estimates:  bias= -4.3129; SE= 2.7496; intercept=0.7545 | 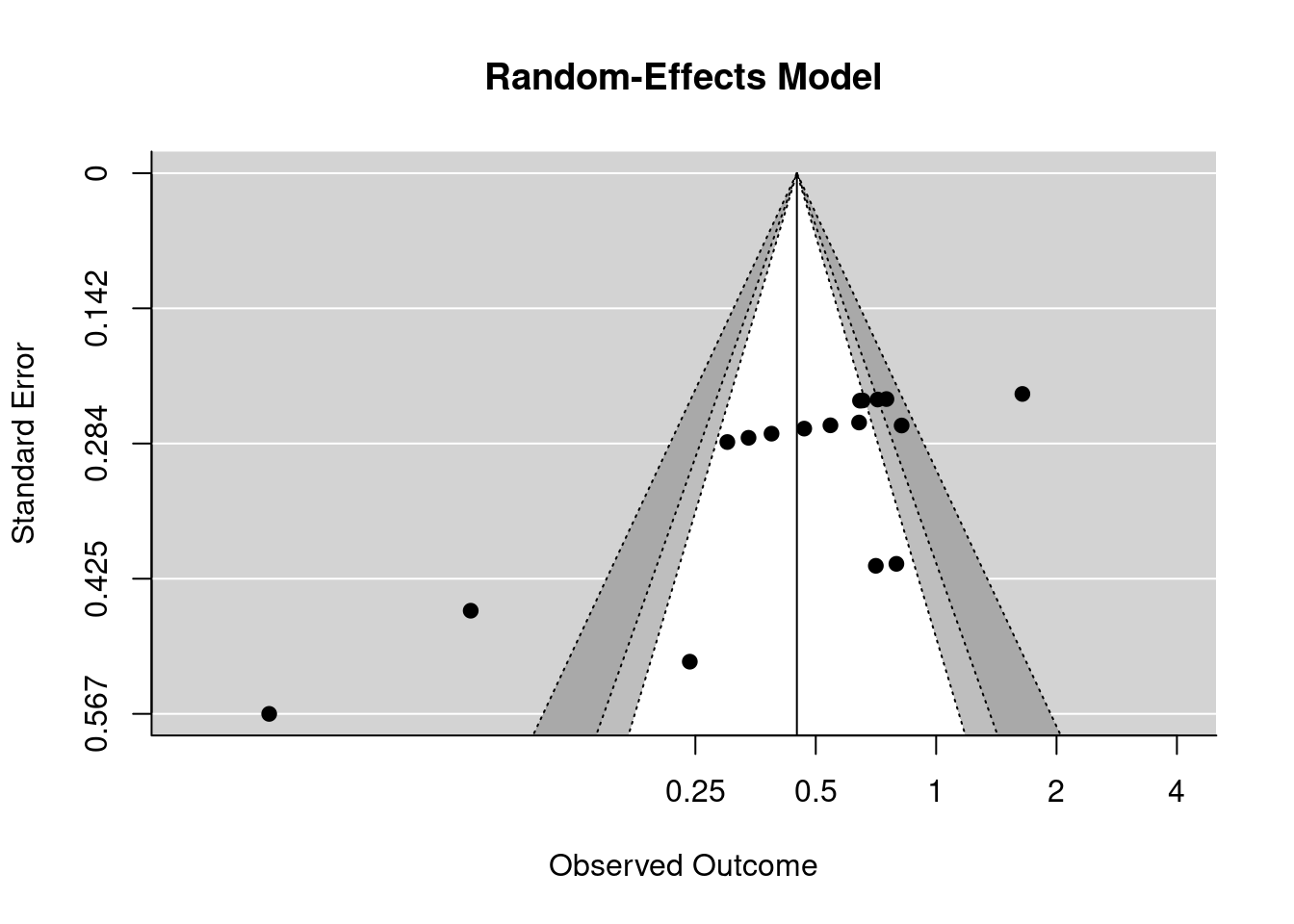  Egger’s regression test of funnel plot asymmetry:  **t = -3.59, df =15, p-value = 0.0027**  Sample estimates:  bias= -6.6412; SE=1.8518; intercept=1.2597 |  |
